# Effectiveness of Digital Serious Games on Knowledge and Attitudes in Public Health Education: Systematic Review and Bayesian Network Meta-Analysis of Randomized Controlled Trials

**DOI:** 10.2196/89281

**Published:** 2026-04-24

**Authors:** Di Huang, Dongjun Wu, Rene Hexel, Christine Brown-Wilson, Jing Zhou, Wendy Moyle

**Affiliations:** 1 School of Nursing and Midwifery, Griffith University, 170 Kessels Road, Brisbane, Queensland, 4111, Australia, 61 0423608125, 61 0423608125; 2 School of Nursing and Midwifery, Griffith University, Gold Coast, Queensland, Australia; 3 School of Information and Communication Technology, Griffith University, Brisbane, Queensland, Australia; 4 School of Nursing and Midwifery, Queen's University Belfast, Belfast, Northern Ireland, United Kingdom; 5 Department of Nursing, The Affiliated Hospital of Zunyi Medical University, Zunyi, Guizhou, China

**Keywords:** digital serious games, public health education, knowledge, attitudes, Bayesian network meta-analysis, randomized controlled trials

## Abstract

**Background:**

Inadequate health literacy and low engagement challenge public health education. Digital serious games show potential to enhance health knowledge and attitudes. However, the comparative effectiveness of different game formats is unclear.

**Objective:**

This study aimed to evaluate and compare the effectiveness of different digital serious game formats in improving public health knowledge and attitudes.

**Methods:**

This systematic review and Bayesian network meta-analysis followed PRISMA (Preferred Reporting Items for Systematic Reviews and Meta-Analyses) 2020 guidelines. Seven databases (PubMed, CINAHL, Embase, PsycINFO, Cochrane Library, Scopus, and Web of Science) were searched from January 2000 to October 2025. An updated search in February 2026 identified no additional studies. Eligible studies were randomized controlled trials (RCTs) involving nonprofessional participants comparing digital serious games with traditional or noninteractive education. Standardized mean differences and 95% credible intervals were pooled using Bayesian network models with random effects. Subgroup analyses examined population characteristics, intervention duration, health topic, and delivery format. Risk of bias was assessed using the Cochrane risk-of-bias tool, and evidence certainty was rated using the Grading of Recommendations Assessment, Development and Evaluation.

**Results:**

Forty randomized controlled trials from 19 countries (N=8764 participants) were included. Digital serious games significantly improved knowledge (standardized mean difference 0.66, 95% CI 0.32‐0.99; *I*²=89.1%) and attitudes (standardized mean difference 0.50, 95% CI 0.27‐0.76; *I*²=80.7%) compared with traditional education. Multisession interventions showed larger effects than single-session interventions for knowledge (0.76 vs 0.43) and attitudes (0.53 vs 0.30), with greater improvements among adolescents, nonpatient populations, and Asian studies. Network meta-analysis showed low heterogeneity (*I*²=8% for knowledge; 3% for attitudes). Mobile app–based, computer-offline, and web-based games ranked highest for knowledge; computer-offline, web-based, and virtual reality games ranked highest for attitudes. Evidence certainty was moderate for knowledge and low-to-moderate for attitudes.

**Conclusions:**

Digital serious games improve public health knowledge and attitudes across diverse contexts. Using a Bayesian network meta-analysis of randomized controlled trials, this review compares the relative effectiveness of different game formats. Mobile app–based, computer-offline, and web-based games most improved knowledge; computer-offline, web-based, and virtual reality formats most improved attitudes. Multisession interventions were more effective than single-session ones, particularly for adolescents and nonpatient populations. These findings guide scalable digital health education strategies. Future research requires adequately powered trials, longer follow-up, and standardized frameworks.

## Introduction

Inadequate health knowledge and attitudes remain a significant barrier to achieving global health targets [1], despite more than a century of organized public health education through mass media campaigns, school-based curricula, and community programs [2,3]. Health literacy gaps are evident across settings and income levels. In the European Union, 27% to 48% of adults have inadequate health literacy [4]. In China, only 31.87% of residents were health literate in 2024 [5], with substantial disparities by residence and education. In the United States, fewer than one-third of school-aged children met grade-level reading standards, indicating persistent barriers to acquiring and applying health information [6,7]. These gaps undermine vaccination and screening, compromise chronic disease (CD) management and medication adherence [8], and contribute to inequities in health outcomes and avoidable health care costs [9,10]. Addressing them requires strategies that can sustain engagement, broaden reach, and adapt to rapidly changing information environments.

Traditional public health education has improved awareness and behaviors, but its impact is limited by low long-term engagement, unequal access, and weak adaptability to rapidly changing communication environments [11,12]. The COVID-19 pandemic magnified these weaknesses, with school closures disrupting learning for more than 1.6 billion learners worldwide and exposing the fragility of knowledge dissemination systems [13]. This interruption also accelerated the adoption of digital health interventions, which offer scalable, interactive, and adaptable complements to conventional programs, extending reach, promoting equitable access, and strengthening public health knowledge, attitudes, and behaviors [14,15].

Within digital health interventions, digital serious games have emerged as a promising strategy to address persistent gaps in participation and impact [16,17]. By combining education with interactive and immersive play, they can sustain engagement and improve knowledge retention, supporting long-term behavioral change [18,19]. Their formats have progressed from desktop programs to mobile apps and online platforms and now increasingly incorporate virtual reality, augmented reality, artificial intelligence, and wearable devices [20-23]. This evolution has enabled broad application in public education, including infectious disease preparedness, CD management, and dementia awareness [24-26]. With their promise of scalability and equitable access [27-29], digital serious games are increasingly regarded as a complement to conventional approaches and digital tools, with potential to support prevention and health promotion at the population level.

Despite this promise, evidence remains insufficient to guide large-scale implementation. A 2020 scoping review mapped digital serious games for health education across health care providers, patients, and the public, showing expansion beyond disease-specific contexts but without assessing relative effectiveness across formats or populations [30]. Other reviews have concentrated on single conditions or target groups, such as diabetes [31], upper limb rehabilitation [32], or vaccination, offering little comparative insight [33]. Existing meta-analyses are similarly constrained, relying on pairwise comparisons that cannot establish the relative effectiveness of multiple intervention types [34,35]. For policymakers and educators, the central question is no longer whether digital serious games can work, but rather which formats are most effective, for which populations, and under what circumstances. No systematic evaluation has yet addressed these comparative questions, leaving a critical gap in the evidence needed to inform equitable and scalable public health education strategies.

To our knowledge, this systematic review and Bayesian network meta-analysis is the first to synthesize and compare the effectiveness of different formats of digital serious games in improving public health knowledge and attitudes and to examine how population characteristics, intervention duration, and contextual factors may moderate their impact.

## Methods

### Information Sources and Search Strategy

For this systematic review and network meta-analysis, we followed PRISMA (Preferred Reporting Items for Systematic Reviews and Meta-Analyses) 2020 and reported the search process in accordance with PRISMA-S (Preferred Reporting Items for Systematic Reviews and Meta-Analyses–Search extension). The completed PRISMA 2020, PRISMA 2020 Expanded, PRISMA-S, and PRISMA for Abstract checklists are provided in Checklist 1. The protocol was registered in PROSPERO (International Prospective Register of Systematic Reviews; CRD420251056704) [36]. All PRISMA 2020 items were reviewed against the manuscript to ensure complete and transparent reporting. We systematically searched 7 electronic databases (PubMed, CINAHL, Embase, PsycINFO, Cochrane Library, Scopus, and Web of Science) for studies published between January 1, 2000, and October 1, 2025, to identify any newly published studies meeting the eligibility criteria. Search terms combined keywords and Medical Subject Headings terms related to serious games, digital games, video games, public health education, knowledge, attitudes, and diseases. The search strategy was refined in accordance with Chapter 4.4 of the *Cochrane Handbook* to maximize sensitivity, including the expansion of controlled vocabulary and additional free-text synonyms. An updated search was conducted in February 2026, and no additional eligible studies were identified. Full search strategies for each database are provided in Multimedia Appendix 1. Reference lists of relevant systematic reviews and meta-analyses were also screened for potentially eligible studies (Multimedia Appendix 1). Gray literature sources and clinical trial registries were not searched separately.

All records were imported into Covidence (Veritas Health Innovation, Melbourne, Australia) for deduplication, screening, and data management. Two reviewers (DH and DW) independently screened titles, abstracts, and full texts, resolving disagreements by consensus or through consultation with a third reviewer (WM). Study authors were contacted when additional clarification was required.

### Eligibility Criteria

Eligibility criteria followed the population, intervention, comparator, outcomes, and study design framework (Multimedia Appendix 2) [37]. We included randomized controlled trials (RCTs), cluster RCTs, and pilot RCTs of digital serious games designed to improve health-related knowledge or attitudes in nonprofessional populations, including children, adolescents, adults, informal caregivers, patients, and the general public. Interventions were required to be delivered via digital platforms, such as web-based applications, mobile apps, computer software, virtual reality, augmented reality, or robot-assisted systems. Comparators included no intervention, conventional education, or digital nongame tools. The primary outcomes were changes in knowledge or attitudes, assessed using validated instruments when reported.

We excluded studies evaluating nondigital games; interventions targeting clinical treatment, rehabilitation, or professional training; and studies that did not report at least 1 primary outcome. Nonrandomized studies, qualitative research, reviews, commentaries, protocols, and conference abstracts were also excluded.

### Data Extraction

A standardized data extraction form was initially developed (DH) and subsequently refined (DH and DW) in accordance with the *Cochrane Handbook for Systematic Reviews of Interventions* [38]. The form was pilot-tested on a subset of studies to ensure reliability and reproducibility before full implementation. For each eligible trial, 2 reviewers (DH and DW) independently extracted data and verified the results with a third reviewer (WM). Extracted variables included study characteristics (identifier, year, country, design, and setting); participant characteristics (population group, mean age, sex distribution, sample size, and patient status); intervention details (type of digital serious game, delivery platform, educational content, duration, and follow-up, if reported); comparator details (type and format); and outcome measures (assessment tools, baseline and postintervention scores, and key findings related to knowledge or attitudes).

### Outcomes

The primary outcomes were changes in health-related knowledge, including understanding of diseases, prevention, and health promotion, as well as changes in health-related attitudes, beliefs, perceptions, and intentions. Both outcomes were assessed using validated instruments when reported. No secondary outcomes were prespecified.

### Bias Risk Assessment

The risk of bias for each included study was assessed using the revised Cochrane risk-of-bias tool for randomized trials tool with the appropriate version applied to individually randomized and cluster-randomized trials [39]. The tool evaluates potential bias in the following domains: the randomization process, deviations from intended interventions, missing outcome data, measurement of the outcomes, and selection of the reported results. Each trial was independently appraised by 2 reviewers (DH and DW) and categorized as low risk, some concerns, or high risk of bias. Any disagreements were resolved by consensus, with persistent discrepancies adjudicated by a third reviewer (WM).

### Statistical Analysis

#### Pairwise Meta-Analysis

Pairwise meta-analyses were first performed to estimate pooled effects. For each study, mean values and SDs of intervention and control groups were extracted. When SDs were not directly reported, they were imputed from SEs, *P* values, *t* values, or 95% CIs. Studies without sufficient information for conversion were excluded from quantitative pooling.

Given the expected heterogeneity across populations, interventions, and outcome measures, pooled standardized mean differences (SMDs) with 95% CIs were calculated using a random-effects model with Hartung-Knapp adjustment to provide more robust variance estimation under conditions of limited study numbers and substantial between-study variability [40].

Between-study heterogeneity was assessed using Cochran *Q* test and quantified using the *I*² statistic [41]. Robustness of pooled estimates in the pairwise meta-analyses was evaluated through sensitivity analyses, including sequential exclusion of individual studies, application of fixed-effect models, and removal of trials at high risk of bias. Prespecified subgroup analyses explored potential sources of heterogeneity, stratified by population group, patient status, health topic, duration, publication decade, geographical region, and sex distribution (Multimedia Appendix 3).

#### Bayesian Network Meta-Analysis

Bayesian network meta-analysis was conducted using random-effects models implemented via Markov chain Monte Carlo simulation [42]. Although the included studies differed in populations, health topics, and intervention formats, all interventions were digital serious games targeting health knowledge or attitudes, supporting the conceptual comparability required for network meta-analysis. The analysis followed a predefined 8-step network meta-analysis workflow, including network construction, Bayesian model estimation, convergence diagnostics, inconsistency assessment, treatment ranking, estimation of relative treatment effects, calculation of prediction intervals, and robustness analysis. Analyses were performed in R software (version 4.5.2; R Foundation for Statistical Computing) using the *gemtc* and *netmeta* packages. Four Markov chains were run in parallel with different initial values, each with 5000 burn-in iterations followed by 20,000 sampling iterations. Convergence was assessed using trace plots and the Gelman-Rubin diagnostic, with a potential scale reduction factor below 1.05 indicating adequate convergence. Noninformative priors were specified for treatment effects, and vague priors were applied to the between-study heterogeneity parameter to minimize prior influence on the model estimates. Model fit was evaluated using the deviance information criterion [43].

Pooled SMDs with corresponding 95% credible intervals (CrIs) were generated for all intervention comparisons. Prediction intervals were additionally calculated to reflect the expected range of effects in future studies. Local inconsistency was assessed using the node-splitting method [44], and global inconsistency was evaluated by comparing the deviance information criterion between the consistency model and the unrelated mean effects model. Between-study heterogeneity was accommodated using the random-effects model and quantified using the between-study variance parameter and overall network *I*². Relative treatment rankings were estimated using the surface under the cumulative ranking curve (SUCRA), mean ranks, and rank probabilities. Sensitivity analyses were conducted by applying alternative prior distributions for the heterogeneity parameter to assess the robustness of the model estimates. Complete R scripts for both pairwise and network meta-analyses are provided in Multimedia Appendix 4.

### Evaluation of Publication Bias

Publication bias and small-study effects were assessed using the Egger regression test (*P*<.10) and comparison-adjusted funnel plots within the network meta-analysis framework. Interpretation of funnel plot asymmetry was undertaken cautiously, as between-study variability and model complexity may contribute to apparent asymmetry, independent of publication bias.

### Certainty of Evidence

The certainty of the evidence was appraised using the GRADE (Grading of Recommendations, Assessment, Development, and Evaluation) framework, following the GRADE Working Group’s guidance. Certainty was evaluated across the domains of study design, risk of bias, inconsistency, indirectness, imprecision, and potential publication bias [45]. All included randomized trials, including pilot and cluster trials, were initially rated as high-certainty evidence. Downgrading was applied when serious limitations were identified, including high risk of bias, substantial unexplained heterogeneity, indirectness of the evidence in relation to the review question, imprecision of effect estimates, or potential publication bias, in accordance with GRADE guidance [46]. Potential publication bias was also assessed. Final ratings were categorized as high, moderate, low, or very low certainty.

## Results

### Study Selection

**Figure 1.**
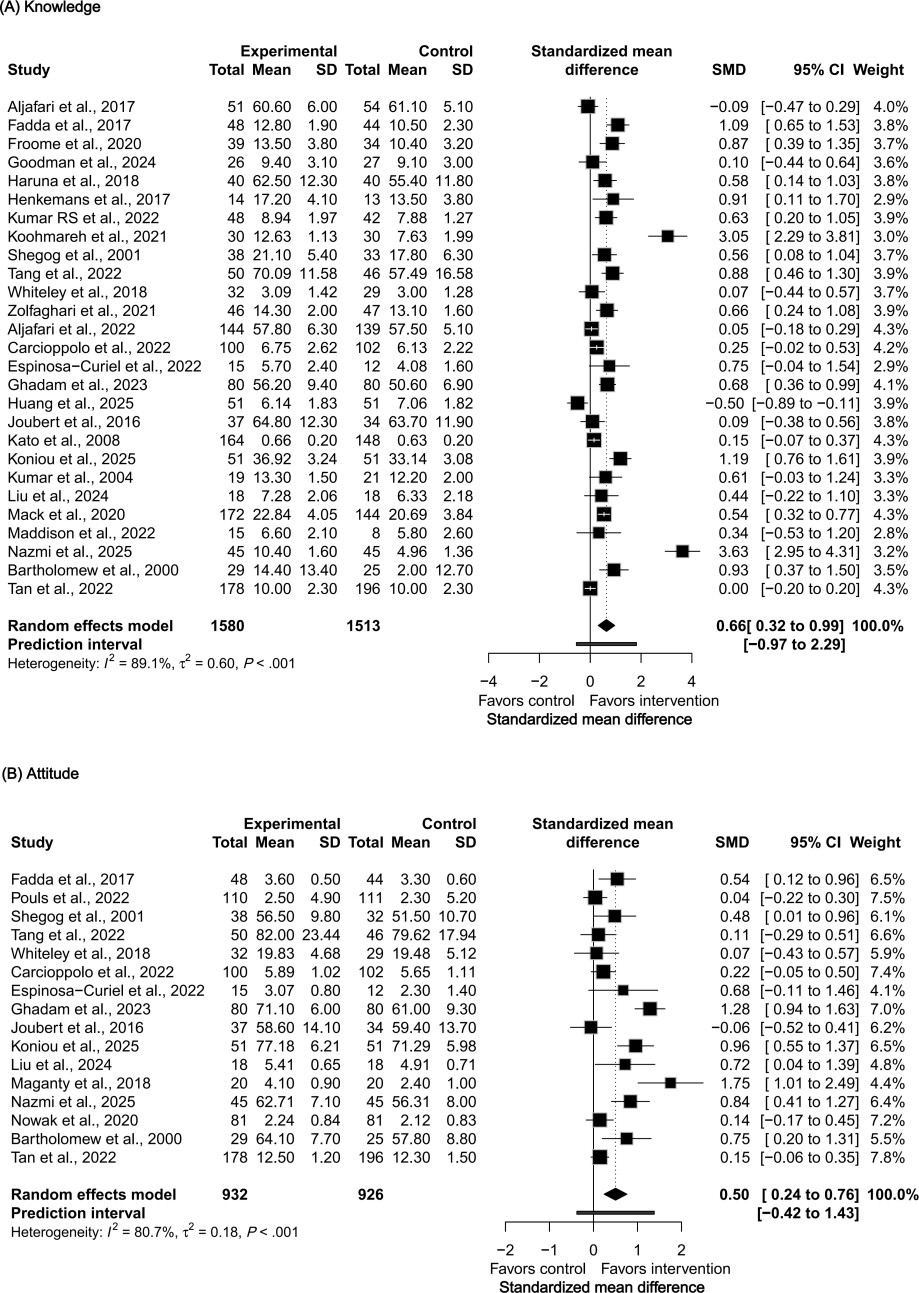
Forest plots showing pooled standardized mean differences (SMDs) for the effects of digital serious games on public health knowledge and attitudes in randomized controlled trials. (A) Knowledge outcomes (27 trials; random-effects model with Knapp-Hartung adjustment: SMD 0.66, 95% CI 0.32‐0.99; prediction interval −0.97 to 2.29; *I*²=89.1%). (B) Attitude outcomes (16 trials; random-effects model with Knapp-Hartung adjustment: SMD 0.50, 95% CI 0.27‐0.76; prediction interval −0.42 to 1.43; *I*²=80.7%). Squares represent individual study effect sizes (size proportional to study weight); horizontal lines represent 95% CIs; diamonds indicate pooled summary estimates [47-75].

**Figure 2.**
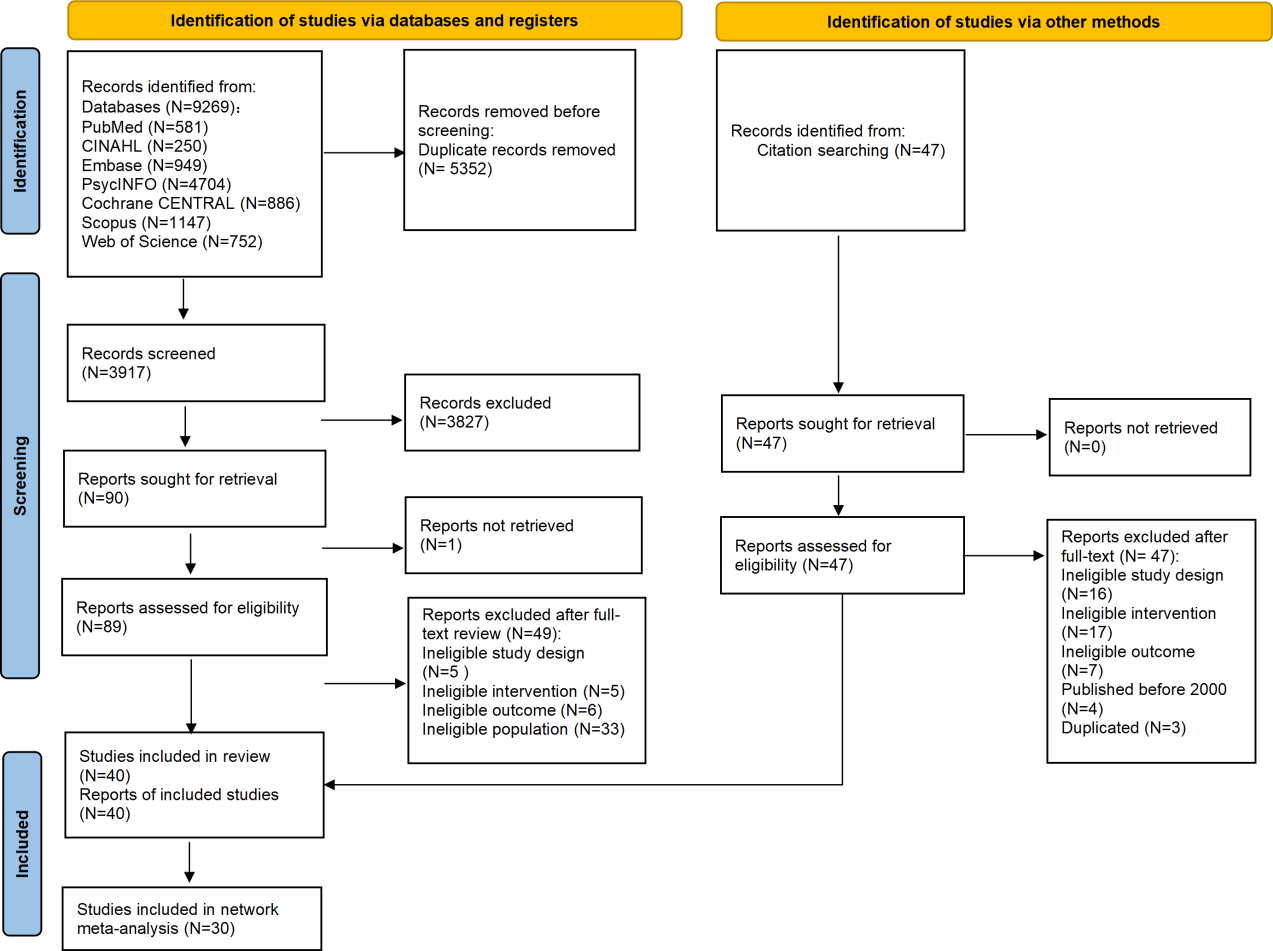
PRISMA flow diagram of study identification, screening, and selection for randomized controlled trials evaluating digital serious games in public health education. PRISMA: Preferred Reporting Items for Systematic Reviews and Meta-Analyses.

The database search yielded 9269 records, and a further 47 records were identified through reference searches of relevant systematic reviews and meta-analyses. After the removal of 5352 duplicates, 3917 records were screened by title and abstract (Figure 1). Of these, 3816 records were excluded, and 1 report could not be retrieved because the full text was unavailable. A total of 88 full-text articles were assessed for eligibility, of which 48 were excluded for reasons summarized in Multimedia Appendix 5. In total, 40 studies were included in the systematic review and 30 in the Bayesian network meta-analysis (Figure 2).

### Study Characteristics

A total of 40 RCTs published between 2000 and 2025 were included (Table 1). Research output increased markedly after 2020, with 5 (12.5%) studies published before 2010 [47-50,76], 10 (22.5%) between 2010 and 2019 [51-57,77-79], and 25 (62.5%) since 2020 [58-75,80-86]. Studies were conducted across 19 countries, most frequently in the United States (n=13, 32.5%) [47-50,54,56,60,65,74,76,78,79,83], followed by China (n=4, 10%) [64,68,70,85], Iran (n=4, 10%) [58,62,67,73], and the Netherlands (n=3, 7.5%) [53,63,77]. All studies adopted a randomized controlled design, including 10 (25%) pilot trials and 5 (12.5%) cluster trials.

The design and characteristics of digital serious games are summarized in Multimedia Appendix 6. Interventions evolved with technological development. Early studies used computer-offline serious games (n=7, 17.5%) [47,49-52,80,85] and video-based serious games (n=6, 15%) [48,61,66,76,78,79]. Later studies adopted computer or web-based online games (n=8, 20%) [55,56,65,71,73,75,77,83] and mobile-app games (n=13, 32.5%) [54,57-59,62-64,67,68,72,81,82,84]. Since 2020, immersive formats such as virtual-reality serious games (n=3, 7.5%) [69,74,86] and augmented-reality serious games (n=2, 5%) [60,71] have become more common, and robot-assisted serious games (n=1, 2.5%) [53] were included from 2017. Three games, *Watch, Discover, Think, and Act* [47,50], *Re-Mission* [76,78], and *Dental Detective* [51,80], were each evaluated in 2 trials due to updated versions or applications in different populations. All iterations were therefore included in the synthesis.

A total of 8764 participants were included (intervention =4374; control =4390). Twelve studies (30%) targeted children, 11 (27.5%) adolescents, 11 (27.5%) adults, and 6 (15%) mixed populations (eg, children and adolescents or adolescents and adults). Thirteen studies (32.5%) included more women than men, 9 (n=9, 22.5%) were gender-balanced, and 18 (45%) included a higher proportion of men.

The most frequent educational topics were CD education (n=8, 20%), cancer education (n=6, 15%), sexual and reproductive health education (n=6, 15%), and nutrition and healthy lifestyle education (n=6, 15%). Other topics included vaccination and infectious-disease prevention (n=4, 10%), oral health education (n=4, 10%), medication and antimicrobial-resistance education (n=3, 7.5%), and psychological and developmental health education (n=3, 7.5%).

Among the 40 studies, 35 (87.5%) reported knowledge outcomes, of which 28 (80%) showed significant improvement and 7 (20%) found no between-group difference [51,54,60,72,75,77,80]. A total of 22 (55%) studies assessed attitude outcomes, with 15 (68.2%) showing positive changes and 7 (31.8%) showing no improvement [51,54,55,57,63,75,80]. A total of 30 (75%) studies were included in the Bayesian network meta-analysis [47-75,80], including those reporting knowledge outcomes (n=27, 67.5%) [47-55,57-62,64-73,75,80] and attitude outcomes (n=16, 40%) [47,50,54-57,63-67,69,70,73-75].

**Table 1.** Characteristics of included randomized controlled trials evaluating digital serious games for public health education.

Study and outcome	Study design	Country	Population	Age (y), mean (SD)	Female, %	Patient status	Intervention	Comparator	Sample size (IG^a^/CG^b^)	Health topic	Duration	Baseline	Key findings
Aljafari et al [51], 2017													
Knowledge	RCT^c^	United Kingdom	Children	6.5 (1.55)	45	Patient	CF^d^	FE^e^	55/54	OH^f^	Single session	IG 56.0 (9.6); CG 53.4 (10.6)	Knowledge increased in both groups, with no significant differences observed between them
Attitude	NA^g^	NA	NA	NA	NA	NA	NA	NA	NA	NA	NA	NA	No significant changes were observed in perceived susceptibility or perceived importance
Aljafari et al [80], 2022													
Knowledge	RCT	Jordan	Children	6.5 (0.5)	48	Nonpatient	CF	NI^h^	144/139	OH	Single session	IG 56.5 (5.9); CG 57.2 (4.9)	Knowledge improved in the intervention group, but no significant difference was found between groups at follow-up
Attitude	NA	NA	NA	NA	NA	NA	NA	NA	NA	NA	NA	NA	Attitudes, intentions, and self-efficacy improved in the IG and were sustained for 6 mo, with no significant change in HIV testing uptake
Bartholomew et al [50], 2000													
Knowledge	Pilot RCT	United States	Children	IG 9.8 (2.1); CG 9.5 (1.9)	35	Patient	CF	NI	29/25	CD^i^	Multiple sessions	IG 76.1 (12.8); CG 78.4 (14.5)	Knowledge and self-management behaviors significantly improved in the IG compared with controls, with greater gains observed in younger children
Attitude	NA	NA	NA	NA	NA	NA	NA	NA	NA	NA	NA	NA	The intervention reduced emergency visits and hospitalizations among participants with moderate-to-severe asthma
Beale et al [76], 2007													
Knowledge	RCT	United States	Adolescents	IG 15.79 (2.62); CG 16.0 (2.89)	32	Patient	VI^j^	AC^k^	191/169	Cancer	Multiple sessions	IG 59.31 (16.9); CG 59.73 (15.6)	Knowledge increased in both groups, with the Re-Mission game showing greater gains than the CG
Beaujean et al [77], 2016													
Knowledge	Cluster RCT	Netherlands	Children	NA	50	Nonpatient	CO^l^	NI	199/372	ID^m^	Single session	NA	Knowledge improved across all groups, with no significant differences between the game, leaflet, and CGs
Bloomfield et al [82], 2025													
Knowledge	Cluster RCT	Australia	Adolescents	NA	50	Nonpatient	AP^n^	FE	442/346	ID	Single session	NA	Knowledge gains in the IG were maintained at 3- and 6-mo follow-up, while the CG remained stable
Boomer et al [83], 2024													
Knowledge	RCT	United States	Adolescents	15.4 (1.2)	48	Nonpatient	CO	OT^o^	145/142	SRH^p^	Multiple sessions	NA	Knowledge increased in the IG compared with controls and was sustained over 12 months
Carcioppolo et al [65], 2022													
Knowledge	RCT	United States	Adults	47.3 (17.6)	53	Nonpatient	CO	NI	100/102	Cancer	Single session	IG 6.75 (2.6); CG 6.13 (2.2)	The game improved melanoma identification compared with the CG
Attitude	NA	NA	NA	NA	NA	NA	NA	NA	NA	NA	NA	IG 5.89 (1.0); CG 5.65 (1.1)	The game enhanced self-efficacy and promoted more positive prevention attitudes
Espinosa-Curiel et al [66], 2022													
Knowledge	Pilot RCT	Mexico	Children	9.9 (0.8)	59	Nonpatient	VI	FE	15/12	NHL^q^	Multiple sessions	IG 5.7 (2.4); CG 4.08 (1.6)	The game improved children’s knowledge of physical activity compared with controls
Attitude	NA	NA	NA	NA	NA	NA	NA	NA	NA	NA	NA	IG 3.07 (0.8); CG 2.3 (1.4)	Attitudes toward sexual health improved, particularly among boys and younger adolescents
Fadda et al [57], 2017													
Knowledge	RCT	Italy	Adults	34.2 (4.7)	94.60	Nonpatient	AP	NI	48/44	Vaccination	Multiple sessions	10.3 (2.1)	Knowledge scores increased significantly in the gamified app group compared with the CG
Attitude	NA	NA	NA	NA	NA	NA	NA	NA	NA	NA	NA	3.4 (0.6)	Vaccination intention and decision confidence improved in the gamified app group, with no significant change in vaccination attitude or recommendation intention
Fiellin et al [79], 2017													
Knowledge	RCT	United States	Adolescent	12.5 (1.1)	50	Nonpatient	VI	AC	166/165	SRH	Multiple sessions	NA	Knowledge improved in the IG, including greater awareness of menstrual hygiene management and contraceptive methods
Froome et al [59], 2020													
Knowledge	Pilot RCT	Canada	Children	9.0 (0.8)	38	Nonpatient	AP	NI	39/34	NHL	Multiple sessions	IG 10.3 (2.9); CG 10.2 (3.1)	Knowledge scores increased significantly in the IG compared with controls, with notable gains in fruits, protein, and whole grains knowledge
Ghadam et al [67], 2023													
Knowledge	RCT	Iran	Adolescents	14.2 (0.7)	100	Nonpatient	AP	TE^r^	80/80	NHL	Multiple sessions	IG 47.9 (9.9); CG 46.6 (7.8)	Knowledge scores increased significantly in the IG compared with controls
Attitude	NA	NA	NA	NA	NA	NA	NA	NA	NA	NA	NA	IG 60.0 (6.7); CG 57.6 (12.9)	Attitude scores were higher in the IG compared with the CG
Goodman et al [60], 2024													
Knowledge	Pilot RCT	United States	Children	10.9 (2.9)	50	Patient	AR^s^	VE^t^	26/27	CD	Single session	IG 9.4 (3.1); CG 9.1 (3.0)	Knowledge increased in both groups, with no significant difference between game and video education
Haruna et al [52], 2018													
Knowledge	Cluster RCT	Malaysia	Children and adolescents	13.6 (1.1)	50	Nonpatient	CF	TE	40/40	SRH	Multiple sessions	IG 62.5 (12.3); CG 55.4 (11.8)	Knowledge scores were significantly higher in the game-based group compared with traditional education
Attitude	NA	NA	NA	NA	NA	NA	NA	NA	NA	NA	NA	NA	Attitude, motivation, and engagement were higher in the game-based learning group than in the CG
Henkemans et al [53], 2017													
Knowledge	Pilot RCT	Netherlands	Children	10.5 (2.0)	50	Patient	RB^u^	TA	14/13	CD	Multiple sessions	IG 17.2 (4.1); CG 13.5 (3.8)	Knowledge scores increased significantly in the robot-assisted game group compared with controls
Attitude	NA	NA	NA	NA	NA	NA	NA	NA	NA	NA	NA	NA	Autonomy, competence, relatedness, motivation, and engagement were higher in the robot-assisted group than in the CG
Huang et al [68], 2025													
Knowledge	RCT	China	Adults	63.5 (11.7)	24.5	Patient	AP	FE	51/51	CD	Multiple sessions	IG 6.14 (1.8); CG 7.06 (1.8)	Knowledge scores improved significantly in the IG compared with the CG at post test
Huang et al [81], 2024													
Knowledge	RCT	Singapore	Adults	36.7 (10.4)	57	Nonpatient	AP	NI	90/90	MAR^v^	Single session	IG 8.5 (1.6); CG 8.3 (1.7)	Knowledge scores were higher in the app-based serious game group compared with the CG
Joubert et al [55], 2016													
Knowledge	Pilot RCT	France	Adolescents	15.5 (1.5)	48	Patient	CO	TA^w^	37/34	CD	Multiple sessions	IG 64.8 (12.3); CG 63.7 (11.9)	Diabetes-related knowledge increased significantly in the IG compared with controls
Attitude	NA	NA	NA	NA	NA	NA	NA	NA	NA	NA	NA	IG 58.6 (14.1); CG 59.4 (13.7)	Positive trends were observed in self-management attitudes and behaviors, though less pronounced than knowledge gains
Kato et al [48], 2008													
Knowledge	RCT	United States	Adolescents and adults	NA	32.3	Patient	VI	NI	164/148	Cancer	Multiple sessions	IG 0.59 (0.2); CG 0.63 (0.2)	Cancer-related knowledge improved significantly in the IG compared with the CG
Khalil et al [78], 2016													
Attitude	RCT	United States	Adolescents	NA	50	Nonpatient	VI	EM^x^	166/50	Cancer	Single session	2.92 (1.2)	Participants in the IG reported higher perceived susceptibility to cancer than controls, and the effect was sustained for 20 d
Koohmareh et al [62], 2021													
Knowledge	RCT	Iran	Adults	IG 52.6 ( 8.4); CG 53.3 (7.9)	56.7	Patient	AP	EM	30/30	NHL	Multiple sessions	IG 7.27 (2.6); CG 7.47 (2.4)	Knowledge increased significantly in the IG compared with the CG
Koniou et al [69], 2025													
Knowledge	RCT	Greece	Adults	20.8 (2.5)	70.8	Nonpatient	VR^y^	NI	51/51	PDH^z^	Single session	IG 36.92 (3.2); CG 33.14 (3.1)	Knowledge scores improved significantly in the VR group compared with the CG
Attitude	NA	NA	NA	NA	NA	NA	NA	NA	NA	NA	NA	IG 77.18 (6.2); CG 71.29 (6.0)	Attitudes toward autism improved significantly in the VR group compared with controls
Kumar et al [49], 2004													
Knowledge	Pilot RCT	United States	Children and adolescents	13.6 (2.5)	45	Patient	CF	NI	19/21	CD	Multiple sessions	NA	Knowledge scores increased significantly in IG compared with CG
Kumar RS et al [61], 2022													
Knowledge	RCT	India	Adolescents	13.62 (1.37)	42	Nonpatient	VI	TE	48/42	OH	Single session	IG 7.29 (1.7); CG 7.30 (1.3)	Knowledge increased significantly in the IG compared with traditional education
Liu et al [70], 2024													
Knowledge	Pilot RCT	China	Children	7.09 (0.9)	38.9	Nonpatient	AR	WN^aa^	18/18	Cancer	Multiple sessions	IG 6.17 (2.3); CG 6.56 (2.1)	Knowledge improved significantly in the IG compared with controls
Attitude	NA	NA	NA	NA	NA	NA	NA	NA	NA	NA	NA	IG 4.98 (0.6); CG 4.95 (0.6)	Attitudes improved significantly in the IG compared with controls
Mack et al [71], 2020													
Knowledge	Cluster RCT	Germany	Children	10.5 (0.5)	50	Nonpatient	CO	TE	172/144	NHL	Multiple sessions	IG 20.77 (4.2); CG 20.28 (3.8)	Knowledge improved significantly in the IG compared with controls after the intervention
Maddison et al [72], 2022													
Knowledge	Pilot RCT	New Zealand	Children and adolescents	11.2 (1.8)	70	Patient	AP	WN	15/8	CD	Multiple sessions	IG 4.6 (6.6); CG 5.8 (2.6)	No significant group differences were found; the intervention was perceived as engaging, but effects were not sustained
Maganty et al [56], 2018													
Attitude	Pilot RCT	United States	Adults	59.1 (15.5)	NA	Nonpatient	CO	NI	20/20	Cancer	Single session	IG 2.3 (1.1); CG 2.2 (1.0)	Confidence in melanoma recognition improved significantly in the game group compared with no intervention
Nazmi et al [73], 2025													
Knowledge	RCT	Iran	Adolescents	12.77 (0.5)	100	Nonpatient	CO	NI	45/45	PDH	Multiple sessions	IG 5.11 (1.3); CG 5.22 (1.0)	Knowledge scores increased significantly in the IG compared with controls
Attitude	NA	NA	NA	NA	NA	NA	NA	NA	NA	NA	NA	IG 61.9 (7.3); CG 57.6 (6.3)	Practice scores improved significantly in the IG compared with the CG
Nowak et al [74], 2020													
Attitude	RCT	United States	Adults	NA	NA	Nonpatient	VR	NI	81/81	Vaccination	Single session	NA	The VR intervention enhanced vaccine confidence, beliefs about community immunity, and vaccination intention compared with the CG
Pouls et al [63], 2022													
Attitude	RCT	Netherlands	Adults	61.2 (11.3)	73	Patient	AP	TA	110/111	MAR	Multiple sessions	IG 5.0 (5.1); CG 5.8 (4.3)	No significant differences were found in medication beliefs or adherence between intervention and CGs
Raj et al [84], 2025													
Knowledge	RCT	India	Adolescents	16.7(0.1)	100	Nonpatient	AP	NI	769/928	SRH	Multiple sessions	NA	Knowledge improved significantly in the IG compared with the CG
Shegog et al [47], 2001													
Knowledge	RCT	United States	Children	10.9 (1.1)	34	Patient	CF	NI	38/33	CD	Single session	IG 18.6 (6.5); CG 15.7 (5.8)	The IG showed greater improvement in asthma self-management knowledge compared with the CG
Attitude	NA	NA	NA	NA	NA	NA	NA	NA	NA	NA	NA	IG 53.4 (9.7); CG 51.6 (9.9)	The IG demonstrated higher self-efficacy and more positive attributions regarding asthma management than the CG
Tan et al [75], 2022													
Knowledge	RCT	Singapore	Adults	35.7 (9.6)	59.6	Nonpatient	CO	WN	178/196	Vaccination	Single session	IG 10.0 (2.3); CG 10.0 (2.3)	Knowledge scores increased in both groups, with no significant difference between them
Attitude	NA	NA	NA	NA	NA	NA	NA	NA	NA	NA	NA	IG 12.5 (1.2); CG 12.3 (1.5)	Attitude scores increased in both groups, with no significant difference between them
Tang et al [64], 2022													
Knowledge	RCT	China	Adolescents	13.5 (0.6)	50	Nonpatient	AP	TE	50/46	SRH	Single session	IG 11.2 (3.4); CG 10.9 (3.2)	Knowledge scores increased significantly in the IG compared with controls
Attitude	NA	NA	NA	NA	NA	NA	NA	NA	NA	NA	NA	NA	The intervention group showed more positive attitudes toward HIV prevention than the CG
Vandeweerdt et al [86], 2022													
Attitude	RCT	Belgium	Adults	NA	NA	Nonpatient	VR	WN	208/208	Vaccination	Single session	NA	Knowledge and perceived health awareness improved significantly in the IG compared with the CG
Wang et al [85], 2025													
Knowledge	Cluster RCT	China	Children	NA	48	Nonpatient	CF	NI	40/39	NHL	Multiple sessions	IG 8.93 (2.4); CG 8.38 (2.1)	The intervention enhanced vaccination intention and collective responsibility compared with text-based education
Whiteley et al [54], 2018													
Knowledge	RCT	United States	Adolescents and adults	22.4 (2.5)	21.3	Patient	AP	NI	32/29	SRH	Multiple sessions	IG 2.44 (1.2); CG 3.00 (1.0)	The IG showed improvements in HIV knowledge, but the difference was not significant
Attitude	NA	NA	NA	NA	NA	NA	NA	NA	NA	NA	NA	IG 17.94 (4.0); CG 18.21 (5.5)	The IG reported slightly higher self-efficacy for ART^ab^ use compared with the CG, but the difference was not significant
Zolfaghari et al [58], 2021													
Knowledge	RCT	Iran	Adolescents and adults	36.4 (4.7)	100	Nonpatient	AP	WN	46/47	OH	Multiple sessions	IG 11.3 (1.9); CG 10.5 (2.1)	The IG had higher knowledge scores than the CG at posttest and 3-month follow-up

a IG: intervention group.

b CG: control group.

c RCT: randomized controlled trial.

d CF: computer offline serious games delivered via PC, tablet, or DVD.

e FE: face-to-face education.

f OH: oral health.

g NA: not available.

h NI: no intervention.

i CD: chronic diseases.

j VI: video-based serious games.

k AC: active control (non–health-related video game).

l CO: computer or web-based online serious games.

m ID: infectious diseases.

n AP: mobile app serious games.

o OT: other serious games.

p SRH: sexual and reproductive health.

q NHL: nutrition and healthy lifestyle.

r TE: traditional classroom or lecture-based education.

s AR: augmented reality serious games.

t VE: educational videos without gamified elements.

u RB: robot-assisted serious games.

v MAR: medication and antimicrobial resistance.

w TA: treatment as usual.

x EM: educational materials (leaflets, booklets, or pamphlets).

y VR: virtual reality serious games.

z PDH: psychological and developmental health.

aa WN: web-based nongame education.

ab ART: antiretroviral therapy.

### Risk-of-Bias Results

Among the 40 included RCTs, methodological quality was generally moderate to high (Multimedia Appendix 7). For the 35 individually randomized trials, low risk of bias was most frequently observed in the randomization process (25/35, 71.4%), deviations from intended interventions (31/35, 88.6%), and measurement of outcomes (33/35, 94.3%). “Some concerns” were mainly identified in the selection of reported results (14/35, 40%) and overall bias judgment (24/35, 68.6%), mainly due to the absence of preregistered protocols or incomplete reporting of secondary outcomes. One trial was rated as high risk of bias in the domain of deviations from intended interventions because participants and facilitators were not blinded during gameplay [51], and another trial was judged as high risk for the same reason, with substantial researcher involvement potentially influencing participant responses [68]. No other study was rated as high risk in any domain. Among the 5 cluster-randomized trials, methodological quality was similarly high; all studies were rated as low risk for the randomization process and missing outcome data, with only minor concerns regarding the selection of the reported result. Taken together, 31.4% (11/35) of studies were judged as low risk, 68.6% (24/35) as having some concerns, and none as high risk.

### Results of the Meta-Analyses

Across the 40 included studies, 27 reported data on knowledge outcomes, and 16 on attitude outcomes [47-75], with 21 trials contributing to both outcome categories. Compared with controls, digital serious games significantly improved public health knowledge (SMD=0.66; 95% CI 0.32‐0.99; *P*<.001; *I*²=89.1%) and showed a moderate positive effect on health attitudes (SMD=0.50; 95% CI 0.27‐0.76; *P*<.001; *I*²=80.7%) (Figure 1). Considerable heterogeneity was observed across studies (knowledge: *Q*=239.23; *P*<.001; attitude: *Q*=77.75; *P*<.001). Funnel plots showed mild asymmetry for both outcomes (Multimedia Appendix 8). Egger’s regression test indicated potential small-study effects for knowledge (*P*=.006) but not for attitude (*P*=.05).

The overall certainty of evidence, assessed using the GRADE framework, was moderate for both knowledge and attitude outcomes. Although substantial heterogeneity was present, the direction of effects remained consistent across studies, and subgroup analyses explained much of the observed variation by intervention duration, population type, and health topic. Minor methodological concerns related to randomization, allocation concealment, and small-study effects contributed to downgrading from high to moderate certainty. Indirectness and imprecision did not materially affect the certainty ratings, as all included trials directly addressed the review question and yielded precise pooled estimates (Multimedia Appendix 9).

### Subgroup and Moderator Analyses

Subgroup analyses were undertaken to explore potential sources of heterogeneity across intervention duration, study region, patient status, health topic, publication year, population type, and sex. Across both knowledge and attitude outcomes, multisession interventions consistently yielded larger effects than single-session exposure (knowledge: *χ*²_1_=4.04; *P*=.04; attitude: *χ*²_1_=4.97; *P*=.03), indicating that repeated game participation reinforced learning and attitude internalization. Effect sizes were also greater among nonpatient populations than among patients (knowledge: *χ*²_1_=7.13; *P*=.008; attitude: *χ*²_1_=9.97; *P*=.002), suggesting that individuals without disease burden may be more receptive to health information. Considerable variation was observed across health topics (knowledge: *χ*²_6_=120.32; *P*<.001; attitude: *χ*²_6_=176.14; *P*<.001), with cancer- and CD-focused games achieving the highest impact, whereas effects were smaller for vaccination and oral health education. Regional differences were modest but favored studies conducted in Asia (*χ*²4=10.18; *P*=.04). Publication year, age group, and sex composition did not consistently influence effect estimates (Multimedia Appendix 10).

### Bayesian Network Meta-Analysis

**Figure 3.**
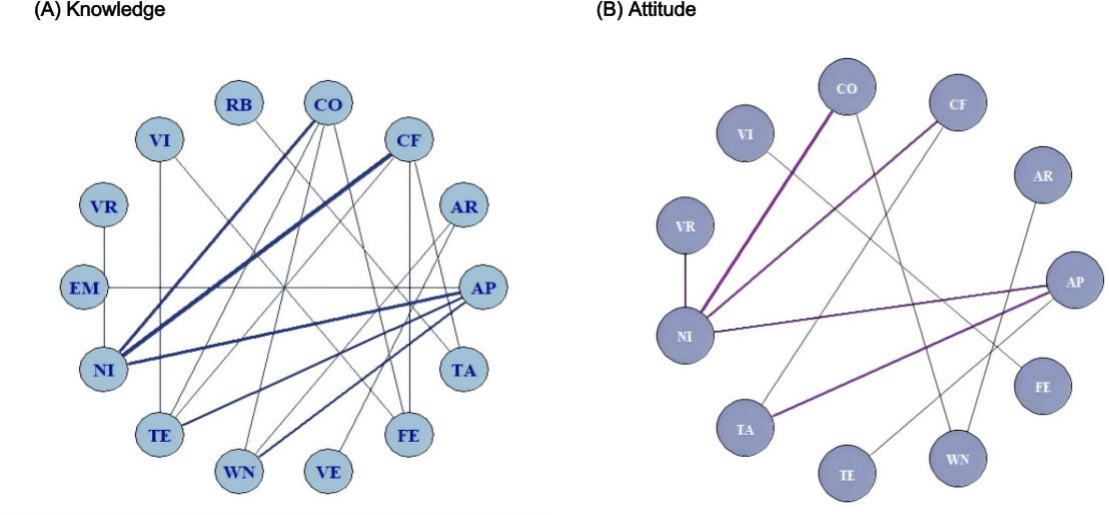
Network structures of digital serious game interventions included in the network meta-analysis for knowledge and attitude outcomes. Panels A and B illustrate the network structures of digital serious game interventions for knowledge and attitude outcomes, respectively. Each node represents an intervention, and each connecting line indicates a direct comparison between interventions in the included randomized controlled trials. The size of each node is proportional to the number of participants receiving that intervention, and the thickness of the connecting lines reflects the number of direct comparisons. AP: mobile app serious games; AR: augmented reality serious games; CF: computer offline serious games delivered via PC, tablet, or DVD; CO: computer or web-based online serious game; EM: educational materials (leaflets, booklets, or pamphlets); FE: face-to-face education; NI: no intervention; RB: robot-assisted serious games; TA: treatment as usual; TE: traditional classroom or lecture-based education; VE: educational videos without gamified elements; VI: video-based serious games; VR: virtual reality serious games; WN: web-based nongame education.

**Figure 4.**
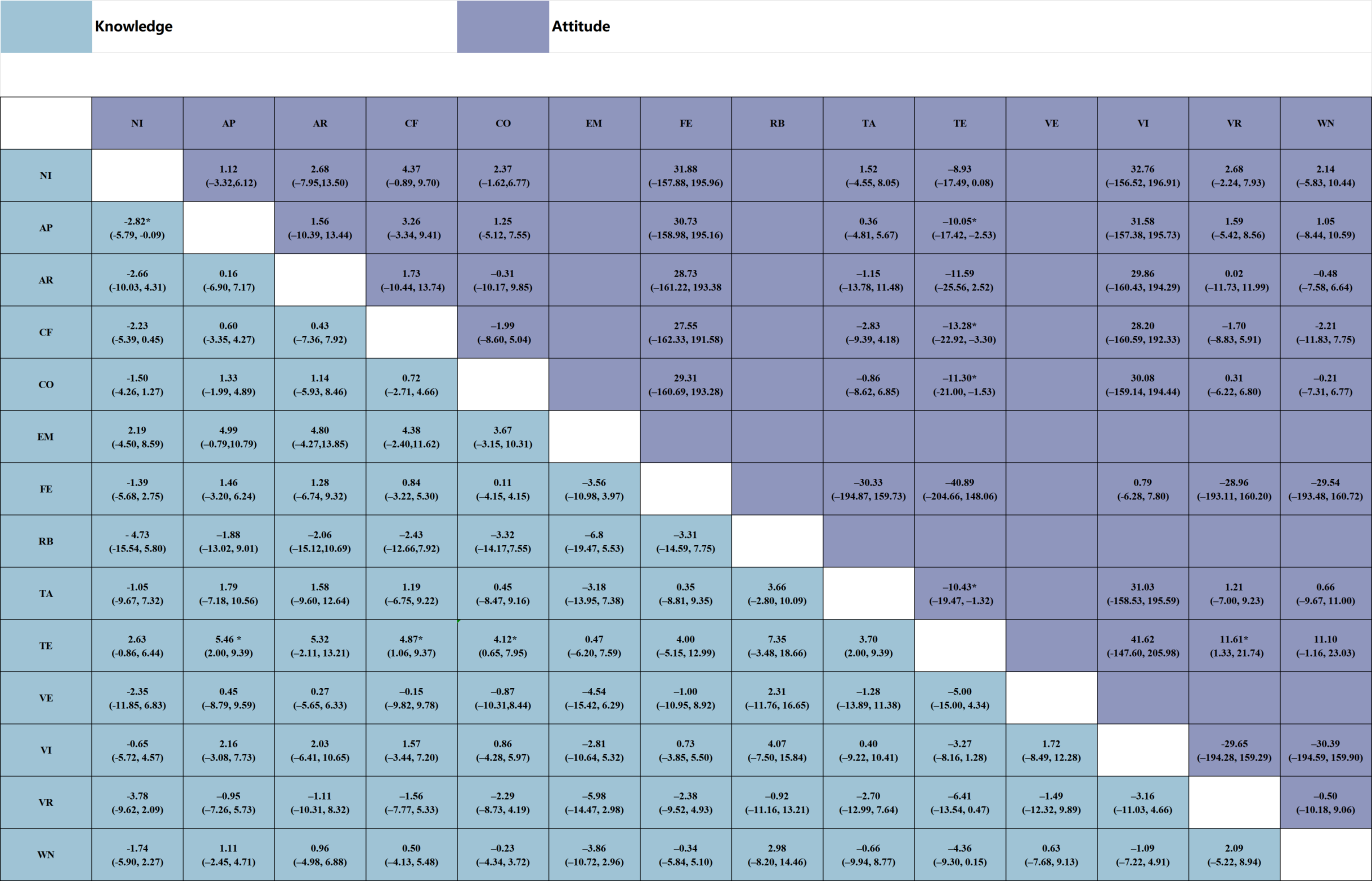
Heatmap of pairwise comparisons from the network meta-analysis of digital serious game interventions for knowledge and attitude outcomes. The heatmap summarizes pairwise mean differences with corresponding 95% credible intervals between interventions. Values below the diagonal represent knowledge outcomes, whereas values above the diagonal represent attitude outcomes. Positive values favor the column-defining intervention, whereas negative values favor the row-defining intervention. Darker shading represents larger absolute mean differences. Statistically significant comparisons are indicated by *(*P*<.05). AP: mobile app serious games; AR: augmented reality serious games; CF: computer offline serious games delivered via PC, tablet, or DVD; CO: computer or web-based online serious game; EM: educational materials (leaflets, booklets, or pamphlets); FE: face-to-face education; NI: no intervention; RB: robot-assisted serious games; TA: treatment as usual; TE: traditional classroom or lecture-based education; VE: educational videos without gamified elements; VI: video-based serious games; VR: virtual reality serious games; WN: web-based nongame education.

**Figure 5.**
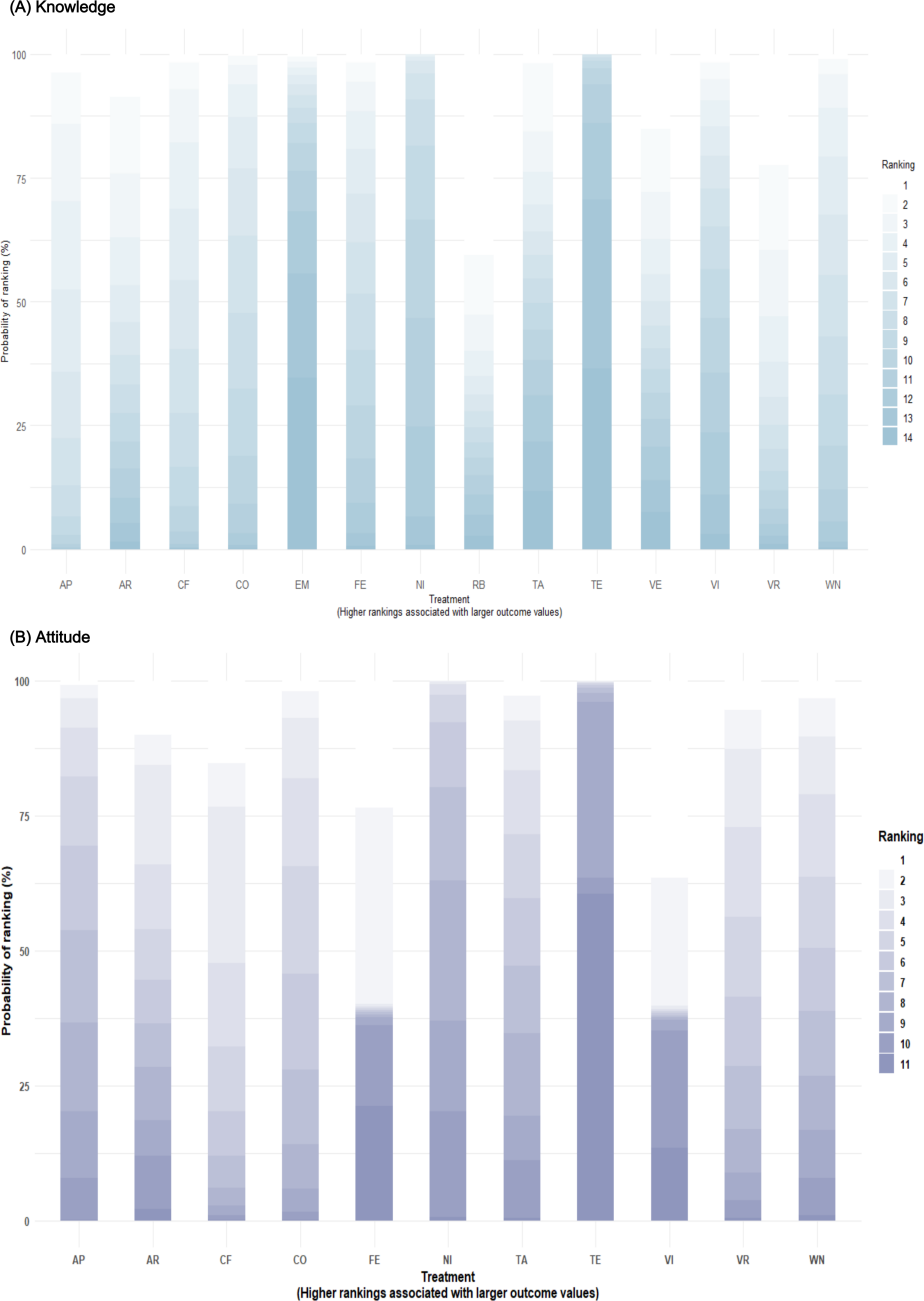
Bayesian ranking probabilities of digital serious game interventions for improving knowledge and attitude outcomes. Panels A and B present the Bayesian ranking probabilities for knowledge and attitude outcomes, respectively. Each stacked bar represents the probability that a given intervention ranks at each possible position among all interventions included in the network meta-analysis. Darker shading indicates higher ranking probabilities. Higher rankings indicate greater effectiveness. AP: mobile app serious games; AR: augmented reality serious games; CF: computer offline serious games delivered via PC, tablet, or DVD; CO: computer or web-based online serious game; EM: educational materials (leaflets, booklets, or pamphlets); FE: face-to-face education; NI: no intervention; RB: robot-assisted serious games; TA: treatment as usual; TE: traditional classroom or lecture-based education; VE: educational videos without gamified elements; VI: video-based serious games; VR: virtual reality serious games; WN: web-based nongame education.

The knowledge network comprised 14 interventions, including 7 types of digital serious games and 7 traditional or nongame comparators, forming 26 direct comparisons and 5 closed loops (Figure 3A). Between-study heterogeneity in the network meta-analysis was low (*τ*=2.75; 95% CrI 1.58‐4.69; τ²=7.57; network *I*²=8%). Digital serious games produced the greatest improvements in knowledge outcomes (Figure 4). Mobile app–based games showed significantly higher effects than traditional education (mean difference 5.46; 95% CrI 2.00‐9.39), treatment as usual (4.87; 95% CrI 1.06‐9.37), and no intervention (2.82; 95% CrI 0.09‐5.79). Computer-offline and web-based serious games also achieved superior gains compared with traditional education (4.87; 95% CrI 0.65‐7.95 and 4.12; 95% CrI 0.79‐6.05, respectively), whereas robot-assisted, virtual reality, and video-based games showed weaker comparative effects. Bayesian ranking analyses indicated that mobile app–based, computer-offline, and web-based serious games consistently ranked highest for improving knowledge outcomes (Figure 5A). Prediction intervals were calculated to reflect the expected range of treatment effects in future studies (Multimedia Appendix 11). No significant inconsistency between direct and indirect evidence was detected in node-splitting analyses (all *P*>.05; Multimedia Appendix 12), and the consistency and unrelated mean effects models showed nearly identical model fit (Deviance Information Criterion; DIC=109.899 vs 109.894; ΔDIC=0.005). Sensitivity analyses using alternative prior distributions produced nearly identical SUCRA values and treatment rankings, indicating robust results (Multimedia Appendix 13).

The attitude network comprised 11 interventions, including 6 types of digital serious games and 5 traditional or nongame comparators, forming 16 direct comparisons and 3 closed loops (Figure 3B). Between-study heterogeneity in the network meta-analysis was low (*τ*=3.19; 95% CrI 1.42‐6.53; *τ*²=10.20; network *I*²=3%). Digital serious games produced greater improvements in health attitudes compared with traditional or nongame education (Figure 4). Computer-offline, web-based, and virtual reality serious games showed the largest improvements in attitude outcomes compared with traditional education (13.28; 95% CrI 3.30‐22.92; 11.30; 95% CrI 1.53‐21.00; and 11.61; 95% CrI 1.33‐21.74, respectively), whereas video-based, face-to-face, and no-intervention conditions showed weaker or inconsistent effects. Bayesian ranking analyses indicated that computer-offline, web-based, and virtual reality serious games ranked highest for improving health attitudes (Figure 5B). Prediction intervals were calculated to reflect the expected range of treatment effects in future studies (Multimedia Appendix 11). No significant inconsistency between direct and indirect evidence was detected in node-splitting analyses (all *P*>.05; Multimedia Appendix 12), and the consistency and unrelated mean effects models showed similar model fit (DIC=58.47 vs 60.12; ΔDIC=1.65). Sensitivity analyses using alternative prior distributions produced nearly identical SUCRA values and treatment rankings, indicating that the results were robust (Multimedia Appendix 13).

## Discussion

### Principal Findings

In this systematic review and Bayesian network meta-analysis of 40 RCTs involving 8764 participants, digital serious games were associated with improvements in public health knowledge and attitudes compared with traditional or noninteractive education. Greater effects were observed with multisession interventions. Subgroup analyses indicated stronger responses among adolescents and nonpatient populations, particularly in studies conducted in Asia and in interventions addressing psychological or developmental health topics. Network meta-analysis further demonstrated differences across delivery formats: mobile app–based games ranked highest for knowledge outcomes, whereas computer-offline and web-based formats showed greater relative effectiveness for attitude change, while video-based and traditional education formats consistently ranked lower. By integrating pairwise and network meta-analysis within a cross-disease framework, this study enables comparative evaluation across formats and population contexts, addressing limitations of prior reviews restricted to single conditions or pairwise comparisons [87-89].

Despite substantial heterogeneity observed in the pairwise meta-analysis, sensitivity analyses confirmed the stability of pooled estimates, indicating that the variability primarily reflects contextual and population-level differences rather than methodological bias. Differences across health topics highlight the influence of content relevance and narrative structure in shaping learning outcomes. Interventions targeting psychological or developmental health often incorporate self-management scenarios and emotionally salient components that may enhance perceived relevance and retention [90]. The advantage of repeated exposure is consistent with reinforcement and memory consolidation processes, whereas single-session interventions may offer insufficient opportunities for feedback and integration. Larger improvements among nonpatient populations may relate to lower baseline knowledge and reduced ceiling effects [91]. The heterogeneity observed, therefore, reflects meaningful contextual differentiation rather than instability of effect estimates.

These findings should also be interpreted considering methodological factors, including the risk of bias and the certainty of evidence. Risk-of-bias assessment using the revised Cochrane risk-of-bias tool for randomized trial tool indicated that several studies had methodological limitations that may have influenced effect estimates. The overall certainty of evidence assessed using the GRADE framework was rated as moderate for knowledge outcomes and low-to-moderate for attitudes. In addition, while CIs represent the average effect across studies, prediction intervals reflect the potential variation in effects across different implementation settings, indicating that intervention effects may vary depending on population characteristics and context.

Beyond these methodological considerations, the findings also suggest several interpretive mechanisms underlying the educational effects of serious games. Although the network meta-analysis suggested relatively consistent comparative effects across formats, the pooled estimates and ranking probabilities should still be interpreted with caution because the included studies varied in populations, health topics, and intervention characteristics. From a structural perspective, the network findings further suggest that the educational impact of serious games may operate along complementary cognitive and affective pathways. Interventions incorporating adaptive feedback, progressive challenge, and opportunities for repeated engagement are more likely to activate sustained cognitive processing, thereby facilitating the consolidation and integration of information [92]. In contrast, formats characterized by low interactivity and fixed content delivery may limit learner control and cognitive activation [93]. With respect to attitudinal outcomes, narrative-driven and role-playing designs appear more conducive to attitude change through mechanisms of perspective-taking and emotional engagement [94], while immersive simulations may intensify affective involvement through first-person experiential framing. Together, these findings indicate that knowledge gains are primarily supported by cognitive reinforcement processes, whereas attitudinal change is more closely linked to emotional immersion and social resonance. The integration of both pathways may, therefore, strengthen the overall educational impact of serious games in public health contexts.

Importantly, the relative balance between cognitive structure and experiential immersion is not only a theoretical distinction but also a practical one. Designs that prioritize deep affective engagement often require greater technological resources and infrastructural investment, whereas cognitively structured, feedback-oriented formats may be more feasible for large-scale dissemination [95]. This interplay between experiential intensity and implementation feasibility becomes particularly salient in population-level public health education [96].

Consistent with this structural tension, the network analysis highlights trade-offs between experiential depth and scalability. Virtual-reality and robot-assisted formats may achieve high experiential fidelity yet face barriers related to cost and accessibility, while mobile and web-based interventions enable broader reach, albeit sometimes with reduced experiential richness [97]. These findings suggest that innovation should not focus solely on increasing technical sophistication but rather on developing adaptive architectures capable of preserving feedback, learner autonomy, and emotional resonance across diverse delivery contexts [98,99]. Achieving equilibrium between structural fidelity and affective relevance may be essential for translating short-term knowledge improvements into sustained behavioral and attitudinal change in population health education.

### Implications for Practice and Research

Evidence from this review suggests that digital serious games can extend the reach of public health education in settings where conventional programs face limitations in coverage or engagement. The consistent advantages of mobile and web-based formats over resource-intensive technologies indicate that scalability depends more on accessibility and design efficiency than on technical sophistication [22,100]. In practice, prioritizing adaptive, feedback-driven mobile platforms may yield greater population impact than investing in high-fidelity but low-reach systems, such as virtual reality or robotics [21,101]. At the same time, stronger effects observed among adolescents and women highlight both the potential for targeted implementation and the need to address equity gaps among patients and older adults who demonstrate lower engagement. Inclusive design, tailored difficulty adjustment, and integration within existing community or school-based programs may help reduce digital exclusion and sustain long-term participation [102,103].

Beyond implementation considerations, the current evidence base remains fragmented, with substantial variation in populations, intervention formats, and outcome measures. Most randomized trials are small and exploratory, often limited to school-aged or student samples. Future research should prioritize adequately powered trials involving adult and older populations and incorporate medium- and long-term follow-up to determine whether gains in knowledge and attitudes translate into sustained behavioral change [30].

A structured implementation framework is also needed to define minimal effective exposure, establish evaluation benchmarks, and clarify ethical standards for educational gaming [104,105]. Standardization represents a critical next step. Although thematic diversity in public health education is expected, a unified evaluation framework should be developed to assess usability, implementation quality, and core design attributes—such as interactivity, feedback mechanisms, immersion, and accessibility—using validated instruments [97,98]. Establishing shared data infrastructures that systematically document intervention characteristics, engagement metrics, and outcome measures would further enhance comparability and enable cumulative synthesis across studies. Strengthening these methodological and infrastructural foundations will be essential for advancing serious game research from isolated trials toward a coherent and reproducible scientific field.

### Limitations

The interpretation of this synthesis should take into account the methodological and conceptual variability among the included trials, which likely contributed to the substantial heterogeneity observed in the pairwise meta-analysis. Health education topics, measurement instruments, and feedback structures differed widely, complicating direct comparison of effect sizes across studies [30]. Although subgroup analyses identified several sources of heterogeneity, the diversity in outcome definitions and analytical strategies inevitably limited the precision of pooled estimates.

In several recent studies, particularly those published after 2020, technological advancements have led serious games, such as *Food Adventure Quest* and *Amoo,* to evolve beyond stand-alone formats, increasingly integrating complementary educational components, such as video segments and classroom instruction [62,85]. Although this convergence complicates the identification of the game’s independent effects, it reflects the growing trend toward multimodal approaches in health education. It indicates that serious games are becoming embedded components of broader digital learning ecosystems that combine interactive games, web-based modules, and instructor-led components.

Reporting transparency also varied across studies. Protocol preregistration and complete reporting of secondary outcomes were often absent, resulting in the overall methodological quality being rated as moderate, despite generally low risks of bias in randomization, intervention delivery, and outcome measurement [106]. These limitations indicate that the main threat to internal validity stems from incomplete documentation rather than flawed trial conduct, underscoring the need for preregistered protocols and comprehensive reporting standards in future evaluations of serious games.

### Conclusions

This systematic review and Bayesian network meta-analysis of RCTs provides a comprehensive evaluation of the educational impact of digital serious games in public health. Unlike earlier reviews that focused on individual interventions or specific health topics, this study compares multiple digital serious game formats within a unified analytical framework. Across 40 trials, mobile-, computer-, and web-based formats generally produced the greatest improvements in knowledge outcomes, while computer-, web-, and virtual reality–based formats showed stronger effects for attitude change. Multisession interventions sustained learning and attitudinal change more effectively than single-session exposure, highlighting the importance of reinforcement and continued engagement. The overall certainty of evidence was moderate, reflecting methodological heterogeneity across trials. These findings contribute comparative evidence to the field of digital health education and offer practical guidance for selecting scalable serious game interventions in real-world public health programs. Strengthening implementation strategies, standardizing outcome evaluation, and extending trials to underrepresented adult and older populations are important next steps.

## Supplementary material

10.2196/89281 Multimedia Appendix 1 Full electronic search strategies for all databases used in this systematic review, including PubMed, CINAHL, Embase, APA PsycINFO, Cochrane CENTRAL, Scopus, and Web of Science.

10.2196/89281 Multimedia Appendix 2 Detailed eligibility criteria for study inclusion and exclusion in this systematic review, including population, intervention, comparator, outcomes, study design, language, and publication type.

10.2196/89281 Multimedia Appendix 3 Subgroup classification and coding schema used for subgroup analyses in this systematic review.

10.2196/89281 Multimedia Appendix 4 R scripts used to conduct the pairwise meta-analysis and Bayesian network meta-analysis, including data preparation, model fitting, subgroup analyses, and sensitivity analyses.

10.2196/89281 Multimedia Appendix 5 Detailed characteristics of the included studies, including study design, sample size, population characteristics, intervention features, and outcome measures.

10.2196/89281 Multimedia Appendix 6 Design features and characteristics of the digital serious games included in this review, including developers, interaction mechanisms, and educational purposes.

10.2196/89281 Multimedia Appendix 7 Risk of bias assessment for included trials, including individually randomized trials and cluster randomized trials.

10.2196/89281 Multimedia Appendix 8 Funnel plots assessing potential publication bias for knowledge and attitude outcomes across the included studies.

10.2196/89281 Multimedia Appendix 9 Grading of Recommendations Assessment, Development and Evaluation summarizing the certainty of evidence for knowledge and attitude outcomes across the included studies.

10.2196/89281 Multimedia Appendix 10 Forest plots of subgroup analyses for knowledge and attitude outcomes across 7 moderators, including intervention duration, study region, and population characteristics.

10.2196/89281 Multimedia Appendix 11 Forest plots of the Bayesian network meta-analysis for knowledge and attitude outcomes showing pooled mean differences with 95% credible intervals.

10.2196/89281 Multimedia Appendix 12 Node-splitting analyses assessing inconsistency between direct and indirect evidence in the network meta-analysis for knowledge and attitude outcomes.

10.2196/89281 Multimedia Appendix 13 Comparison of surface under the cumulative ranking curve values and treatment rankings under uniform and half-normal prior distributions for knowledge and attitude outcomes.

10.2196/89281 Checklist 1 PRISMA reporting checklists used in this review, including the PRISMA 2020 checklist, PRISMA 2020 expanded checklist, PRISMA 2020 for abstracts checklist, and PRISMA-S checklist.

## Abbreviations

CD: chronic diseases
CrI: credible interval
GRADE: Grading of Recommendations Assessment, Development and Evaluation
PRISMA: Preferred Reporting Items for Systematic Reviews and Meta-Analyses
PRISMA-S: Preferred Reporting Items for Systematic Reviews and Meta-Analyses–Search extension
RCT: randomized controlled trial
SMD: standardized mean difference
SUCRA: surface under the cumulative ranking curve
