# Supplementary material for: Effectiveness of Digital Serious Games on Knowledge and Attitudes in Public Health Education: Systematic Review and Bayesian Network Meta-Analysis of Randomized Controlled Trials
Source: J Med Internet Res. 2026 Apr 24;28:e89281. doi: 10.2196/89281 (PMC13108840; doi:10.2196/89281)
Supplement: Multimedia Appendix 1 [file jmir-v28-e89281-s001.docx]

**Multimedia Appendix 2.** Database search strategies. **PubMed Search Strategy**

| **Date of search: May 31, 2025** | | |
| --- | --- | --- |
| **#** | **Search terms** | **Results** |
| **1** | "knowledge"[tiab] OR "awareness"[tiab] OR "belief*"[tiab] OR "perception*"[tiab] OR "intention*"[tiab] | 1,798,468 |
| **2** | "attitude*"[tiab] OR "health education"[MeSH Terms] OR "health literacy"[MeSH Terms] | 486016 |
| **3** | #1 OR #2 | 2116861 |
| **4** | "serious game*"[tiab] OR "video game*"[tiab] OR "computer game*"[tiab] OR "digital game*"[tiab] OR "educational game*"[tiab] | 9614 |
| **5** | "mobile game*"[tiab] OR "online game*"[tiab] OR "VR game*"[tiab] OR "AR game*"[tiab] OR "gamification"[tiab] OR "serious games"[MeSH Terms] | 3553 |
| **6** | #4 OR #5 | 12523 |
| **7** | randomized controlled trial[pt] OR controlled clinical trial[pt] OR randomized[tiab] OR randomised[tiab] OR "randomly allocated"[tiab] | 1247522 |
| **8** | \|  \| \| --- \|  \| ("2000/01/01"[Date - Publication]: "2025/05/31"[Date - Publication]) \| \| --- \| | 25,395,180 |
| **9** | #3 AND #6 AND #7 AND #8 | 546 |
| **10** | English[lang] |  |
| **11** | #9 AND #10 | 544 |
| **Date of search: Oct 1, 2025** | | |
| **12** | ("2025/06/01"[Date - Publication]: "2025/10/01"[Date - Publication]) | 689,993 |
| **13** | #3 AND #6 AND #7 AND #10 AND #12 | 37 |
| **Date of search: Feb 1, 2026** | | |
| 14 | ("Knowledge"[Mesh] OR "Attitude to Health"[Mesh] OR "Health Education"[Mesh] OR "Health Literacy"[Mesh] OR knowledge[tiab] OR awareness[tiab] OR belief*[tiab] OR perception*[tiab] OR attitude*[tiab] OR intention*[tiab]) AND ("Serious Games"[Mesh] OR "Video Games"[Mesh] OR serious game*[tiab] OR serious gaming[tiab] OR video game*[tiab] OR computer game*[tiab] OR digital game*[tiab] OR educational game*[tiab] OR mobile game*[tiab] OR online game*[tiab] OR VR game*[tiab] OR virtual reality game*[tiab] OR AR game*[tiab] OR augmented reality game*[tiab] OR gamification[tiab] OR game-based learning[tiab] OR digital simulation*[tiab]) AND (randomized controlled trial[pt] OR controlled clinical trial[pt] OR randomized[tiab] OR randomised[tiab] OR randomly[tiab] OR trial[tiab]) NOT (animals[mh] NOT humans[mh]) AND ("2000/01/01"[Date - Publication] : "2026/02/01"[Date - Publication]) AND English[lang] | 1053 |

**CINAHL Search Strategy**

| **Date of search: May 31, 2025** | | |
| --- | --- | --- |
| **#** | **Search terms** | **Results** |
| **1** | TI(knowledge OR awareness OR belief* OR perception* OR intention* OR attitude*) OR AB(knowledge OR awareness OR belief* OR perception* OR intention* OR attitude*) OR (MH "Health Education+") OR (MH "Health Literacy") |  |
| **2** | TI("serious game*" OR "video game*" OR "computer game*" OR "digital game*" OR "educational game*" OR "mobile game*" OR "online game*" OR "VR game*" OR "AR game*" OR gamification) OR AB("serious game*" OR "video game*" OR "computer game*" OR "digital game*" OR "educational game*" OR "mobile game*" OR "online game*" OR "VR game*" OR "AR game*" OR gamification) OR (MH "Serious Games") |  |
| **3** | randomized controlled trial OR controlled clinical trial OR randomi* OR "randomly allocated" |  |
| **4** | \|  \| \| --- \|  \| Limiters: Published Date from 20000101–20250531; English language \| \| --- \| |  |
| **5** | #1 AND #2 AND 3 AND #4 | 235 |
| **Date of search: Oct 1, 2025** | | |
| **10** | Limiters: Published Date from 20000101–20250531; English language |  |
| **11** | #3 AND #6 AND #7 AND #10 | 15 |
| **Date of search: Feb 1, 2026** | | |
| 12 | ((MH "Knowledge+") OR (MH "Attitude to Health") OR (MH "Health Education+") OR (MH "Health Literacy") OR TI(knowledge OR awareness OR belief* OR perception* OR intention* OR attitude*) OR AB(knowledge OR awareness OR belief* OR perception* OR intention* OR attitude*)) AND ((MH "Serious Games") OR (MH "Video Games") OR TI("serious game*" OR "serious gaming" OR "video game*" OR "computer game*" OR "digital game*" OR "educational game*" OR "mobile game*" OR "online game*" OR "VR game*" OR "virtual reality game*" OR "AR game*" OR "augmented reality game*" OR gamification OR "game-based learning" OR "digital simulation*") OR AB("serious game*" OR "serious gaming" OR "video game*" OR "computer game*" OR "digital game*" OR "educational game*" OR "mobile game*" OR "online game*" OR "VR game*" OR "virtual reality game*" OR "AR game*" OR "augmented reality game*" OR gamification OR "game-based learning" OR "digital simulation*")) AND (MH "Randomized Controlled Trials" OR MH "Clinical Trials+" OR TI(randomized OR randomised OR randomly OR trial OR "controlled trial") OR AB(randomized OR randomised OR randomly OR trial OR "controlled trial")) Limiters: Published Date from 20000101–20260201; English Language | 423 |

**Ovid Embase Search Strategy**

| **Date of search: May 31, 2025** | | |
| --- | --- | --- |
| **#** | **Search terms** | **Results** |
| **1** | 'knowledge':ab,ti OR 'awareness':ab,ti OR 'belief*':ab,ti OR 'perception*':ab,ti OR 'intention*':ab,ti | **2271276** |
| **2** | 'attitude*':ab,ti OR 'health education'/exp OR 'health literacy'/exp | 693199 |
| **3** | #1 OR #2 | 2,731,839 |
| **4** | 'serious game*':ab,ti OR 'video game*':ab,ti OR 'computer game*':ab,ti OR 'digital game*':ab,ti OR 'educational game*':ab,ti | 11,544 |
| **5** | 'mobile game*':ab,ti OR 'online game*':ab,ti OR 'vr game*':ab,ti OR 'ar game*':ab,ti OR 'gamification':ab,ti OR 'serious game'/exp | 3,738 |
| **6** | \|  \| \| --- \|   #4 OR 5 | 14,786 |
| **7** | 'randomized controlled trial'/exp OR 'controlled clinical trial'/exp OR random*:ab,ti OR 'randomly allocated':ab,ti | 2,818,946 |
| **8** | [2000-01-01]/sd NOT [2025-05-31]/sd |  |
| **9** | #3 AND #6 AND #7 AND #8 | 813 |
| **10** | #19 AND [english]/lim | 807 |
| **Date of search: Oct 1, 2025** | | |
| **11** | \| [2025-06-01]/sd NOT [2025-10-01]/sd \| \| --- \| |  |
| **12** | #9 AND #11 |  |
| **13** | #12 AND [english]/lim | 142 |
| **Date of search: Feb 1, 2026** | | |
| 14 | (('knowledge'/exp OR 'attitude to health'/exp OR 'health education'/exp OR 'health literacy'/exp OR knowledge:ab,ti OR awareness:ab,ti OR belief*:ab,ti OR perception*:ab,ti OR attitude*:ab,ti OR intention*:ab,ti) AND ('serious game'/exp OR 'video game'/exp OR serious game*:ab,ti OR serious gaming:ab,ti OR video game*:ab,ti OR computer game*:ab,ti OR digital game*:ab,ti OR educational game*:ab,ti OR mobile game*:ab,ti OR online game*:ab,ti OR vr game*:ab,ti OR virtual reality game*:ab,ti OR ar game*:ab,ti OR augmented reality game*:ab,ti OR gamification:ab,ti OR 'game based learning':ab,ti OR digital simulation*:ab,ti) AND ('randomized controlled trial'/exp OR 'controlled clinical trial'/exp OR random*:ab,ti OR randomly:ab,ti OR trial:ab,ti)) NOT ('animal'/exp NOT 'human'/exp) AND [2000-01-01]/sd AND [english]/lim | 1540 |

**APA PsycInfo Search Strategy**

| **Date of search: May 31, 2025** | | |
| --- | --- | --- |
| **#** | **Search terms** | **Results** |
| **1** | (knowledge or awareness or belief* or perception* or intention*).mp. [mp=title, abstract, heading word, table of contents, key concepts, original title, tests & measures, mesh word] | **1312767** |
| **2** | (attitude* or "health education" or "health literacy").mp. [mp=title, abstract, heading word, table of contents, key concepts, original title, tests & measures, mesh word] | 630186 |
| **3** | #1 OR #2 | 1652470 |
| **4** | ("serious game*" or "video game*" or "computer game*" or "digital game*" or "educational game*").mp. [mp=title, abstract, heading word, table of contents, key concepts, original title, tests & measures, mesh word] | 17408 |
| **5** | ("mobile game*" or "online game*" or "VR game*" or "AR game*" or "gamification").mp. [mp=title, abstract, heading word, table of contents, key concepts, original title, tests & measures, mesh word] | 3823 |
| **6** | \|  \| \| --- \|   (#4 or #5).mp. [mp=title, abstract, heading word, table of contents, key concepts, original title, tests & measures, mesh word] #4 OR #5 | 853917 |
| **7** | (randomized controlled trial or randomised or "randomly allocated" or "controlled clinical trial").mp. [mp=title, abstract, heading word, table of contents, key concepts, original title, tests & measures, mesh word] | 49604 |
| **8** | **#**3 and **#**6 and **#**7 | 4772 |
| **9** | limit 8 to (english language and yr="2000 - Current") | 4594 |
| **Date of search: Oct 1, 2025** | | |
| **11** | limit 8 to (english language and yr="2000 - Current ") | 4704 |
| **Date of search: Feb 1, 2026** | | |
| 12 | ((DE "Knowledge" OR DE "Attitudes" OR DE "Health Education" OR DE "Health Literacy" OR knowledge.mp. OR awareness.mp. OR belief*.mp. OR perception*.mp. OR attitude*.mp. OR intention*.mp.) AND (DE "Serious Games" OR DE "Video Games" OR serious game*.mp. OR serious gaming.mp. OR video game*.mp. OR computer game*.mp. OR digital game*.mp. OR educational game*.mp. OR mobile game*.mp. OR online game*.mp. OR VR game*.mp. OR virtual reality game*.mp. OR AR game*.mp. OR augmented reality game*.mp. OR gamification.mp. OR game-based learning.mp. OR digital simulation*.mp.) AND (DE "Treatment Effectiveness Evaluation" OR randomized controlled trial.mp. OR controlled clinical trial.mp. OR randomi?ed.mp. OR randomly.mp. OR trial.mp.)) AND limit to (English language AND yr="2000 - Current ") | 5410 |

**Cochrane Central Register of Controlled Trials Search Strategy**

| **Date of search: May 31, 2025** | | |
| --- | --- | --- |
| **#** | **Search terms** | **Results** |
| **1** | knowledge OR awareness OR belief* OR perception* OR intention* | 177550 |
| **2** | attitude* OR "health education" OR "health literacy" | 53135 |
| **3** | #1 OR #2 | 202998 |
| **4** | serious NEXT game* OR video NEXT game* OR computer NEXT game* OR digital NEXT game* OR educational NEXT game* OR mobile NEXT game* OR online NEXT game* OR VR NEXT game* OR AR NEXT game* OR gamification OR serious NEXT games | 4811 |
| **5** | ("randomized controlled trial" OR "controlled clinical trial" OR randomized OR randomised OR "randomly allocated") | 1405745 |
| **6** | #3 AND #4 AND #5 | 983 |
| **7** | Limit 6 to English language | 870 |
| **8** | **Limit 7 to yr=”** **Jan 1, 2000 - Current”** | 850 |
| **Date of search: Oct 1, 2025** | | |
| **11** | \| limit 7 to (english language and yr=" **June 1, 2025** – Current ") \| \| --- \| | 36 |
| **Date of search: Feb 1, 2026** | | |
| 12 | ((knowledge OR awareness OR belief* OR perception* OR attitude* OR intention* OR "health education" OR "health literacy") AND ("serious game*" OR "serious gaming" OR "video game*" OR "computer game*" OR "digital game*" OR "educational game*" OR "mobile game*" OR "online game*" OR "VR game*" OR "virtual reality game*" OR "AR game*" OR "augmented reality game*" OR gamification OR "game-based learning" OR "digital simulation*") AND (randomized OR randomised OR "controlled trial" OR "clinical trial" OR randomly OR trial)) with Publication Year from 2000 to 2026, in English | 921 |

**Scopus Search Strategy**

| **Date of search: May 31, 2025** | | |
| --- | --- | --- |
| **#** | **Search terms** | **Results** |
| **1** | TITLE-ABS-KEY (knowledge OR awareness OR belief* OR perception* OR intention* OR attitude* OR "health education" OR "health literacy") |  |
| **2** | TITLE-ABS-KEY ("serious game*" OR "video game*" OR "computer game*" OR "digital game*" OR "educational game*" OR "mobile game*" OR "online game*" OR "VR game*" OR "AR game*" OR gamification) |  |
| **3** | TITLE-ABS-KEY (randomized OR randomised OR "controlled trial" OR "clinical trial") |  |
| **4** | #1 AND #2 AND #3 |  |
| **5** | LIMIT-TO (LANGUAGE, "English") |  |
| **6** | \|  \| \| --- \|  \| LIMIT-TO (PUBYEAR, 2000- Current) \| \| --- \| | 1,014 |
| **Date of search: Oct 1, 2025** | | |
| **11** | LIMIT-TO (PUBYEAR, 2000- Current) | 1,147 |
| **Date of search: Feb 1, 2026** | | |
| 12 | (TITLE-ABS-KEY (knowledge OR awareness OR belief* OR perception* OR attitude* OR intention* OR "health education" OR "health literacy") AND TITLE-ABS-KEY ("serious game*" OR "serious gaming" OR "video game*" OR "computer game*" OR "digital game*" OR "educational game*" OR "mobile game*" OR "online game*" OR "VR game*" OR "virtual reality game*" OR "AR game*" OR "augmented reality game*" OR gamification OR "game-based learning" OR "digital simulation*") AND TITLE-ABS-KEY (randomized OR randomised OR randomly OR trial OR "controlled trial" OR "clinical trial")) AND LIMIT-TO (LANGUAGE, "English") AND LIMIT-TO (PUBYEAR, 2000- Current) | 1257 |

**Web of Science Search Strategy**

| **Date of search: May 31, 2025** | | |
| --- | --- | --- |
| **#** | **Search terms** | **Results** |
| **1** | TS=(knowledge OR awareness OR belief* OR perception* OR intention* OR attitude* OR "health education" OR "health literacy") |  |
| **2** | TS=("serious game*" OR "video game*" OR "computer game*" OR "digital game*" OR "educational game*" OR "mobile game*" OR "online game*" OR "VR game*" OR "AR game*" OR gamification) |  |
| **3** | TS=(randomized OR randomised OR "controlled trial" OR "clinical trial") |  |
| **4** | #1 AND #2 AND #3 |  |
| **5** | Refined by: Languages=(English) |  |
| **6** | \|  \| \| --- \|  \| Refined by: Publication Years=(2000–2025) \| \| --- \| | 742 |
| **Date of search: Oct 1, 2025** | | |
| **11** | \| Refined by: Publication Years=(2000–2025) \| \| --- \| | 752 |
| **Date of search: Feb 1, 2026** | | |
| 12 | TS=(knowledge OR awareness OR belief* OR perception* OR intention* OR attitude* OR "health education" OR "health literacy")  AND TS=("serious game*" OR "serious gaming" OR "video game*" OR "computer game*" OR "digital game*" OR "educational game*" OR "mobile game*" OR "online game*" OR "VR game*" OR "virtual reality game*" OR "AR game*" OR "augmented reality game*" OR gamification OR "game-based learning" OR "digital simulation*") AND TS=(randomized OR randomised OR randomly OR trial OR "controlled trial" OR "clinical trial") Refined by: Languages=(English) Refined by: Publication Years=(2000–2026) | 801 |

**References for Relevant Systematic Reviews & Meta-analyses**

| **Date of search: May 31, 2025** | | |
| --- | --- | --- |
| **#** | **Reference** | **Results** |
| **1** | Montagni I, Mabchour I, Tzourio C. Digital gamification to enhance vaccine knowledge and uptake: scoping review. *JMIR Serious Games* 2020; 8: e16983. | **7** |
| **2** | Charlier N, Zupancic N, Fieuws S, et al. Serious games for improving knowledge and self-management in young people with chronic conditions: a systematic review and meta-analysis. J Am Med Inform Assoc 2016; 23: 230–9 | 9 |
| **3** | Putri Y H S, Maryati I, Solehati T. Interventions to improve sexual and reproductive health-related knowledge and attitudes among adolescents: scoping review. Risk Manag Healthc Policy 2025; 18: 105–16. | 13 |
| **4** | Nørlev J, Sondrup K, Derosche C, et al. Game mechanisms in serious games that teach children with type 1 diabetes how to self-manage: a systematic scoping review. J Diabetes Sci Technol 2022; 16: 1253–69. | 18 |
| **Total** |  | 47 |

Note: No new relevant systematic reviews or meta-analyses on serious games for public health education were identified in the updated search conducted in February 2026.
