# Supplementary material for: Effectiveness of Digital Serious Games on Knowledge and Attitudes in Public Health Education: Systematic Review and Bayesian Network Meta-Analysis of Randomized Controlled Trials
Source: J Med Internet Res. 2026 Apr 24;28:e89281. doi: 10.2196/89281 (PMC13108840; doi:10.2196/89281)
Supplement: Multimedia Appendix 2 [file jmir-v28-e89281-s002.docx]

**Multimedia Appendix 3.** Eligibility Criteria for Study Inclusion

| **Category** | **Inclusion criteria** | **Exclusion criteria** |
| --- | --- | --- |
| **Population** | Members of the general public, including adolescents and adults without a medical background. Studies involving informal caregivers may be included if the intervention aims to improve public awareness or disease-related understanding. | Studies focusing solely on patients, healthcare professionals, or medical students. Studies with mixed populations but without stratified analysis. |
| **Intervention** | Digital serious games (computer, mobile, VR/AR, or web-based) explicitly designed to improve knowledge or attitudes related to diseases or health conditions. | Non-digital games (e.g., board or card games), or non-gamified digital tools such as text messaging, apps, or websites without interactive or game-based elements. |
| **Comparator** | Standard or conventional health education, usual care, or no intervention. | Comparators that involve similar digital serious games at different exposure rates or durations. |
| **Outcomes** | Studies reporting quantitative outcomes on disease-related knowledge and/or attitudes (pre–post or between-group change). | Studies not reporting knowledge or attitude outcomes, or reporting only behavioral or clinical indicators (e.g., vaccination uptake, BMI, skill performance). |
| **Time** | Studies with interventions conducted and published from January 1, 2000 to May 31, 2025. | Studies published before 2000. |
| **Study design** | Randomized controlled trials (RCTs), including cluster or pilot RCTs with clearly defined control groups. | Non-randomized or observational designs (e.g., quasi-experimental, pre–post without control, cross-sectional, qualitative, protocols, reviews, case series). |
| **Language** | Publications written in English. | Non-English language studies. |
| **Publication type** | Full-text, peer-reviewed journal articles reporting original intervention findings. | Abstracts, conference papers, reviews, commentaries, editorials, animal or simulation studies, unpublished or retracted articles. |
