# Supplementary material for: Effectiveness of Digital Serious Games on Knowledge and Attitudes in Public Health Education: Systematic Review and Bayesian Network Meta-Analysis of Randomized Controlled Trials
Source: J Med Internet Res. 2026 Apr 24;28:e89281. doi: 10.2196/89281 (PMC13108840; doi:10.2196/89281)
Supplement: Multimedia Appendix 3 [file jmir-v28-e89281-s003.docx]

**Multimedia Appendix 4.** Subgroup classification and coding schema

| **Subgroup variable** | **Definition and classification criteria** | **Description** |
| --- | --- | --- |
| **Intervention duration** | Studies were classified according to the exposure frequency of the serious game intervention. | Single-session interventions involved one-time exposure, while multi-session interventions included two or more exposures to assess cumulative learning or reinforcement effects. |
| **Study region** | Grouping was based on the geographical location of the study to capture cultural and contextual variation. | Asia (China, India, Malaysia, Iran, Singapore, Japan); Europe (UK, Italy, Netherlands, France, Germany, Greece); North America (USA, Canada, Mexico); Oceania (Australia, New Zealand); other regions not listed above. |
| **Patient status** | Participants were classified according to their health condition. | Patient group included individuals diagnosed with a specific disease or condition, whereas non-patient group comprised healthy individuals or the general public. |
| **Health topic** | Studies were categorised according to their primary educational focus, aligned with WHO health education domains. | Cancer; chronic diseases; vaccination; infectious diseases; infectious diseases/vaccination; medication and antimicrobial resistance; nutrition and healthy lifestyle; oral health; psychological and developmental health; sexual and reproductive health. |
| **Publication year** | Classification was based on the year of publication to examine temporal and technological trends in serious game research. | Divided into three periods: 2000–2009, 2010–2019, and 2020–2025. |
| **Population type** | Studies were grouped according to participants’ developmental stage and age range. | Children (<13 years), adolescents (13–17 years), and adults (≥18 years). Mixed groups (children & adolescents, adolescents & adults) were used when age categories overlapped. |
| **Sex composition** | Classification was based on the proportion of female participants reported in the study sample. | Groups included female <50%, female = 50%, female > 50%, or not reported. |
