# Supplementary material for: Effectiveness of Digital Serious Games on Knowledge and Attitudes in Public Health Education: Systematic Review and Bayesian Network Meta-Analysis of Randomized Controlled Trials
Source: J Med Internet Res. 2026 Apr 24;28:e89281. doi: 10.2196/89281 (PMC13108840; doi:10.2196/89281)
Supplement: Multimedia Appendix 4 [file jmir-v28-e89281-s004.docx]

**Multimedia Appendix 5.** R scripts for pairwise and network meta-analysis.

All statistical analyses were performed in R (version 4.5.2).

The following script summarises the full workflow used to conduct pairwise meta-analyses and Bayesian network meta-analyses of digital serious games for public health education, covering both knowledge and attitude outcomes. Analyses were implemented using the meta, metafor, BUGSnet, and ggplot2 packages. All data and code are reproducible and available upon reasonable request from the corresponding author.
**Data Preparation**
library(readxl)

library(dplyr)

library(tidyr)

library(meta)

library(BUGSnet)

library(ggplot2)
1. Load datadata_know <- read_excel("H:/review 2/meta-data-knowledge.xlsx")

data_att <- read_excel("H:/review 2/meta-data-attitude.xlsx")

# 2. Convert to wide format

to_wide <- function(df){

df %>%

pivot_wider(names_from = `Digital tool_META`,

values_from = c(Mean, SD, SampleSize),

names_sep = ".") %>%

rename(Mean.IG = Mean.IG, SD.IG = SD.IG, SampleSize.IG = SampleSize.IG,

Mean.CG = Mean.CG, SD.CG = SD.CG, SampleSize.CG = SampleSize.CG)

}

data_know_wide <- to_wide(data_know)

data_att_wide <- to_wide(data_att)

# 3. Primary analysis – Knowledge

m_know <- metacont(n.e = SampleSize.IG, mean.e = Mean.IG, sd.e = SD.IG,

n.c = SampleSize.CG, mean.c = Mean.CG, sd.c = SD.CG,

studlab = Study,

data = data_know_wide,

sm = "SMD", method.smd = "Hedges",

method.tau = "REML", method.random.ci = "HK")

summary(m_know)

forest(m_know)

# 4. Primary analysis – Attitude

m_att <- metacont(n.e = SampleSize.IG, mean.e = Mean.IG, sd.e = SD.IG,

n.c = SampleSize.CG, mean.c = Mean.CG, sd.c = SD.CG,

studlab = Study,

data = data_att_wide,

sm = "SMD", method.smd = "Hedges",

method.tau = "REML", method.random.ci = "HK")

summary(m_att)

forest(m_att)

# 5. Subgroup analysis function

run_subgroup <- function(meta_obj, varname){

update.meta(meta_obj, subgroup = !!as.name(varname))

}

# Knowledge – seven prespecified subgroups

m_know_pop <- run_subgroup(m_know, "Population")

m_know_stat <- run_subgroup(m_know, "Patient status")

m_know_cont <- run_subgroup(m_know, "Health education content")

m_know_dur <- run_subgroup(m_know, "Duration")

m_know_years <- run_subgroup(m_know, "Years")

m_know_reg <- run_subgroup(m_know, "Region")

m_know_sex <- run_subgroup(m_know, "Sex")

# Attitude – seven prespecified subgroups

m_att_pop <- run_subgroup(m_att, "Population")

m_att_stat <- run_subgroup(m_att, "Patient status")

m_att_cont <- run_subgroup(m_att, "Health education content")

m_att_dur <- run_subgroup(m_att, "Duration")

m_att_years <- run_subgroup(m_att, "Years")

m_att_reg <- run_subgroup(m_att, "Region")

m_att_sex <- run_subgroup(m_att, "Sex")

**# Part B. Bayesian Network Meta-Analysis**

# Step 1. Load packages & import data

library(gemtc)
library(netmeta)
library(readxl)
library(dplyr)
library(coda)

# Import dataset
data <- read_excel("meta-data.xlsx")

# Required variables:
# study | treatment | mean | sd | n

# Step 2. Network construction

pair <- pairwise(
treat = treatment,
mean = mean,
sd = sd,
n = n,
studlab = study,
data = data
)

net <- netmeta(
TE = pair$TE,
seTE = pair$seTE,
treat1 = pair$treat1,
treat2 = pair$treat2,
studlab = pair$studlab,
sm = "MD"
)

# Network plot
pdf("Network_plot.pdf", width = 6, height = 6)
netgraph(net)
dev.off()

# Step 3. Bayesian network meta-analysis

data_gemtc <- data

colnames(data_gemtc)[colnames(data_gemtc) == "sd"] <- "std.dev"
colnames(data_gemtc)[colnames(data_gemtc) == "n"] <- "sampleSize"

network <- mtc.network(data.ab = data_gemtc)

model <- mtc.model(
network,
type = "consistency",
likelihood = "normal",
link = "identity",
linearModel = "random"
)

results <- mtc.run(
model,
n.adapt = 5000,
n.iter = 20000
)

summary(results)

# Step 4. Convergence diagnostics

gelman_diag <- gelman.diag(results)
print(gelman_diag)

pdf("Gelman_plot.pdf")
gelman.plot(results)
dev.off()

pdf("Trace_plot.pdf")
plot(results)
dev.off()
# Step 5. Node-splitting inconsistency test

nodesplit <- mtc.nodesplit(network)

res_nodesplit <- mtc.run(nodesplit)

summary(nodesplit)

pdf("Nodesplit_plot.pdf")
plot(summary(nodesplit))
dev.off()

# Step 6. Treatment ranking (Rank probability & SUCRA)

rank_prob <- rank.probability(
results,
preferredDirection = 1
)

sucra_values <- sucra(rank_prob)

print(sucra_values)

pdf("Rankogram.pdf", width = 10, height = 8)
plot(rank_prob, beside = TRUE)
dev.off()

# Step 7. Relative treatment effects & prediction intervals

rel_eff <- relative.effect(
results,
t1 = results$model$network$treatments$id[1]
)

summary_rel <- summary(rel_eff)

write.csv(
as.data.frame(summary_rel$summaries),
"NMA_Effect_Sizes.csv"
)

# Step 8. Robustness analysis

### Global inconsistency test (Consistency vs UME model)

model_cons <- mtc.model(
network,
type = "consistency",
linearModel = "random"
)

res_cons <- mtc.run(
model_cons,
n.adapt = 5000,
n.iter = 20000
)

model_ume <- mtc.model(
network,
type = "ume",
linearModel = "random"
)

res_ume <- mtc.run(
model_ume,
n.adapt = 5000,
n.iter = 20000
)

dic_cons <- res_cons$dic$totres + res_cons$dic$pD
dic_ume <- res_ume$dic$totres + res_ume$dic$pD

delta_dic <- dic_cons - dic_ume

cat("Consistency model DIC:", dic_cons,"\n")
cat("UME model DIC:", dic_ume,"\n")
cat("Delta DIC:", delta_dic,"\n")

### Prior sensitivity analysis

model_sens <- mtc.model(
network,
type = "consistency",
linearModel = "random"
)

model_sens$hy.prior <- list("std.dev", "dnorm", 0, 1)

res_sens <- mtc.run(
model_sens,
n.adapt = 5000,
n.iter = 20000
)

rank_main <- rank.probability(results)
rank_sens <- rank.probability(res_sens)

sucra_main <- sucra(rank_main)
sucra_sens <- sucra(rank_sens)

sensitivity_table <- data.frame(
Treatment = names(sucra_main),
Main_SUCRA = round(as.numeric(sucra_main),4),
Sensitivity_SUCRA = round(as.numeric(sucra_sens),4)
)

write.csv(
sensitivity_table,
"Sensitivity_analysis.csv",
row.names = FALSE
)
**# Part C. Heterogeneity & Consistency Assessment**

# Knowledge

nma.nodesplit(nma_know) # Local consistency (node-splitting)

nma.fit(nma_know)$DIC # Global model fit

nma.fit(nma_know)$residuals # Residual diagnostics

# Attitude

nma.nodesplit(nma_att)

nma.fit(nma_att)$DIC

nma.fit(nma_att)$residuals

**# Part D. Sensitivity Analysis**

# Example: exclusion of high-risk studies

data_know_sens <- data_know %>% filter(!Study %in% c("High-risk study name"))

nma_data_sens <- data.prep(data_know_sens,

varname.t = "Digital tool",

varname.s = "Study",

varname.y = "Mean",

varname.sd = "SD",

varname.n = "SampleSize")

nma_sens <- nma.run(nma_data_sens, trt.effect = "SMD",

reference = "NI", n.adapt = 5000, n.iter = 20000)

nma.rank(nma_sens, SUCRA = TRUE)

***All analyses were conducted following the methods described in the main text. The dataset paths shown are placeholders and should be adjusted when reproducing the analysis.***
