# Supplementary material for: Effectiveness of Digital Serious Games on Knowledge and Attitudes in Public Health Education: Systematic Review and Bayesian Network Meta-Analysis of Randomized Controlled Trials
Source: J Med Internet Res. 2026 Apr 24;28:e89281. doi: 10.2196/89281 (PMC13108840; doi:10.2196/89281)
Supplement: Multimedia Appendix 5 [file jmir-v28-e89281-s005.docx]

**Multimedia Appendix 6.** Studies Excluded at Full-Text Review with Reasons

| **Reports Not Retrieved** | | | |
| --- | --- | --- | --- |
| **No.** | **Reference** | | **Reports not retrieved** |
| **1** | Montagni I, Mabchour I, Tzourio C. Digital gamification to enhance vaccine knowledge and uptake: scoping review. *JMIR Serious Games* 2020; 8: e16983. | | **Conference abstract** |
| **Study Identified from Databases but Excluded with Reasons** | | | |
| **No.** | **Reference** | | **Reports not retrieved** |
| **1** | Lee JJ, Tsang VWY, Chan MMK, O’Connor S, Lokmic-Tomkins Z, Ye F, et al.Virtual reality simulation-enhanced blood transfusion education for undergraduate nursing students: a randomised controlled trial. Nurse Educ Today 2023; 129: 105903. | | **Ineligible population** |
| **2** | Ryan G, Rafferty A, Murphy J, Higgins MF, Mangina E, McAuliffe FM.Virtual reality learning: a randomized controlled trial assessing medical student knowledge of fetal development. Int J Gynaecol Obstet 2023; 162: 292–9. | | **Ineligible population** |
| **3** | Zhang J, Xing J, Zheng M, Sheng J, Zhang K, Zhang B. Effectiveness of virtual simulation and jaw model for undergraduate periodontal teaching. BMC Med Educ 2021; 21: 616. | | **Ineligible population** |
| **4** | Carrión C, Fernández-Alemán JL, Toval A, Fernández-Luque L, Orellana-Rodríguez O. Virtual reality in cardiopulmonary resuscitation training: a randomized trial. Med Intensiva 2022; 46: 345–51. | | **Ineligible population** |
| **5** | Xie Z, Chen F, Zou L, Wang F, Yang L. Using virtual reality in the care of older adults with dementia: a randomized controlled trial. J Gerontol Nurs 2023; 49: 25–32. | | **Ineligible population** |
| **6** | Kane D, Ryan G, Mangina E, McAuliffe FM. A randomized controlled trial of a virtual reality learning environment in obstetric medical student teaching. Int J Med Inform 2022; 168: 104899. | | **Ineligible population** |
| **7** | Mitchell G, Leonard L, Carter G, Santin O, Brown Wilson C. Evaluation of a serious game on nursing student knowledge and uptake of influenza vaccination. PLoS One 2021; 16: e0245389 | | **Ineligible population** |
| **8** | Hu L, Zhang L, Yin R, Li Z, Shen J, Tan H, Wu J, Zhou W. NEOGAMES: a serious computer game that improves long-term knowledge retention of neonatal resuscitation in undergraduate medical students. Front Pediatr 2021; 9: 645776. | | **Ineligible population** |
| **9** | Lemos M, Wolfart S, Rittich AB. Assessment and evaluation of a serious game for teaching factual knowledge in dental education. BMC Med Educ 2023; 23: 521. | | **Ineligible population** |
| **10** | Phungoen P, Promto S, Chanthawatthanarak S, Maneepong S, Apiratwarakul K, Kotruchin P, et al. Precourse preparation using a serious smartphone game on advanced life support knowledge and skills: a randomized controlled trial. J Med Internet Res 2020; 22: e16987. | | **Ineligible population** |
| **11** | Razaghpoor A, Taheri-Ezbarami Z, Jafaraghaee F, Maroufizadeh S, Falakdami A. The effect of a serious game and problem-based learning on nursing students’ knowledge and clinical decision-making skill regarding transfusion medicine in pediatric nursing. J Pediatr Nurs 2024; 76: e1–8. | | **Ineligible population** |
| **12** | Sarvan S, Efe E. The effect of neonatal resuscitation training based on a serious game simulation method on nursing students’ knowledge, skills, satisfaction and self-confidence: a randomized controlled trial. Nurse Educ Today 2022; 111: 105298. | | **Ineligible population** |
| **13** | Ribeiro RT, Cotta RM, Torres HC, Leal DB, Dias A, Nogueira JAD, et al. InsuOnline, an electronic game for medical education on insulin therapy: a randomized controlled trial with primary care physicians. J Med Internet Res 2017; 19: e11. | | **Ineligible population** |
| **14** | Nasirzade A, Deldar K, Froutan R, Shakeri MT. Comparison of the effects of Burn Assessment Mission Game with feedback lecture on nursing students’ knowledge and skills in the burn patients’ assessment: a randomized clinical trial. BMC Med Inform Decis Mak 2024; 24: 157. | | **Ineligible population** |
| **15** | Wan Yusoff WSY, Abdul Zuki FA, Abu MN, Md Saad WM. Enhancing Trichomonas vaginalis identification in cytology students through gamification. J Cytol 2024; 41: 75–83. | | **Ineligible population** |
| **16** | Kerfoot BP, Baker H. An online spaced-education game for global continuing medical education: a randomized trial. Ann Surg 2012; 256: 33–8. | | **Ineligible population** |
| **17** | Lagro J, van de Pol MH, Laan A, Huijbregts-Verheyden FJ, Fluit LC, Olde Rikkert MG. A randomized controlled trial on teaching geriatric medical decision making and cost consciousness with the serious game GeriatriX. J Am Med Dir Assoc 2014; 15: 957.e1–6. | | **Ineligible population** |
| **18** | Calik A, Kapucu S. The effect of serious games for nursing students in clinical decision-making process: a pilot randomized controlled trial. Games Health J 2022; 11: 30–7. | | **Ineligible population** |
| **19** | Telner D, Bujas-Bobanovic M, Chan D, Chester B, Marlow B, Meuser J, Rothman A, Harvey B. Game-based versus traditional case-based learning: comparing effectiveness in stroke continuing medical education. Can Fam Physician 2010; 56: e345–51. | | **Ineligible population** |
| **20** | Del Blanco Á, Torrente J, Fernández-Manjón B, Ruiz P, Giner M. Using a videogame to facilitate nursing and medical students’ first visit to the operating theatre: a randomized controlled trial. Nurse Educ Today 2017; 55: 45–53. | | **Ineligible population** |
| **21** | Tan AJQ, Lee CCS, Lin PY, Cooper S, Lau LST, Chua WL, Liaw SY. Designing and evaluating the effectiveness of a serious game for safe administration of blood transfusion: a randomized controlled trial. Nurse Educ Today 2017; 55: 38–44. | | **Ineligible population** |
| **22** | Chang YS, Hu SH, Kuo SW, Chang KM, Kuo CL, Nguyen TV, Chuang YH. Effects of board game play on nursing students’ medication knowledge: a randomized controlled trial. Nurse Educ Pract 2022; 63: 103412. | | **Ineligible population** |
| **23** | Elzeky MEH, Elhabashy HMM, Ali WGM, Allam SME. Effect of gamified flipped classroom on improving nursing students’ skills competency and learning motivation: a randomized controlled trial. BMC Nurs 2022; 21: 316. | | **Ineligible population** |
| **24** | Ordu Y, Çalışkan N. The effects of virtual gaming simulation on nursing students’ diagnosis, goal setting, and diagnosis prioritization: a randomized controlled trial. Nurse Educ Pract 2023; 68: 103593. | | **Ineligible population** |
| **25** | Othman SY, Ghallab E, Eltaybani S, Mohamed AM. Effect of using gamification and augmented reality in mechanical ventilation unit of critical care nursing on nurse students’ knowledge, motivation, and self-efficacy: a randomized controlled trial. Nurse Educ Today 2024; 142: 106329. | | **Ineligible population** |
| **26** | Aktaş N, Baykara ZG, Öztürk D. The effect of education provided with the escape room game on nursing students’ learning of parenteral drug administration. Nurse Educ Pract 2024; 80: 104133. | | **Ineligible population** |
| **27** | Mohammadi A, Aazami S, Azizifar A. Nursing students learn vaccination using Kahoot gamification: an intervention study of knowledge, satisfaction, interest, and collaboration Nurs Res Pract 2025; 2025: 3518943 | | **Ineligible population** |
| **28** | Buajeeb W, Reynolds PA, Boontub H, Tangmanpuwadol Y, Sipiyaruk K. Comparison of the effectiveness of a serious game and prerecorded lecture in diagnosis and treatment planning of oral lesions for dental students Sci Rep 2024; 14: 30641 | | **Ineligible population** |
| **29** | Tubelo RA, Portella FF, Gelain MA, de Oliveira MMC, de Oliveira AEF, Dahmer A, Pinto MEB. Serious game is an effective learning method for primary health care education of medical students: a randomized controlled trial Int J Med Inform 2019; 130: 103944 | | **Ineligible population** |
| **30** | Shadan M, Ismail H, Naushad FHM. Diabe-teach: a randomized controlled trial of a gamified approach to enhance medical undergraduates’ knowledge and comprehension of diabetes mellitus BMC Med Educ 2025; 25: 17 | | **Ineligible population** |
| **31** | Nasirzade A, Deldar K, Froutan R, Shakeri MT. Comparison of the effects of burn assessment mission game with feedback lecture on nursing students’ knowledge and skills in the burn patients’ assessment: a randomized clinical trial BMC Med Inform Decis Mak 2024; 24: 157 | | **Ineligible population** |
| **32** | Phungoen P, Promto S, Chanthawatthanarak S, Maneepong S, Apiratwarakul K, Kotruchin P, Mitsungnern T. Precourse preparation using a serious smartphone game on advanced life support knowledge and skills: randomized controlled trial J Med Internet Res 2020; 22: e16987 | | **Ineligible population** |
| **33** | Schultz K, Klein M, Sucharew H, McDonald J, DeBlasio D, Cooperstein E, Poynter S, Huggins J, Real FJ. The impact of a gamified curriculum using Kahoot! on musculoskeletal knowledge and skill acquisition among pediatric residents Acad Pediatr 2022; 22: 1265–70 | | **Ineligible population** |
| **34** | Zolfaghari M, Shirmohammadi M, Shahhosseini H, Mokhtaran M, Mohebbi SZ. Development and evaluation of a gamified smartphone mobile health application for oral health promotion in early childhood: a randomized controlled trial. BMC Oral Health 2021; 21: 18. | | **Ineligible intervention** |
| **35** | Homer C, Susskind O, Alpert HR, Owusu M, Schneider L, Rappaport LA, Rubin DH. An evaluation of an innovative multimedia educational software program for asthma management: randomized controlled trial. Pediatrics 2000; 106: 210–15. | | **Ineligible intervention** |
| **36** | McPherson AC, Glazebrook C, Forster D, James C, Smyth A. A randomized, controlled trial of an interactive educational computer package for children with asthma. Pediatrics 2006; 117: 1046–54. | | **Ineligible intervention** |
| **37** | Inangil D, Dincer B, Kabuk A. Effectiveness of the use of animation and gamification in online distance education during the pandemic. Comput Inform Nurs 2022; 40: 335–40. | | **Ineligible intervention** |
| **38** | Martínez-García G, Ewing AC, Olugbade Y, DiClemente RJ, Kourtis AP. Crush: a randomized trial to evaluate the impact of a mobile health app on adolescent sexual health. J Adolesc Health 2023; 72: 287–94. | | **Ineligible intervention** |
| **39** | Wong RS, Tung KTS, Ho FKW, Wong WHS, Chow CB, Chan KL, et al. Effect of a mobile game-based intervention to enhance child safety: randomized controlled trial. J Med Internet Res 2024; 26: e51908. | | **Ineligible outcome** |
| **40** | Cates JR, Fuemmeler BF, Stockton LL, Diehl SJ, Crandell JL, Coyne-Beasley T. Evaluation of a serious video game to facilitate conversations about human papillomavirus vaccination for preteens: pilot randomized controlled trial. JMIR Serious Games 2020; 8: e16883. | | **Ineligible outcome** |
| **41** | Bellens A, Roelant E, Sabbe B, Peeters M, van Dam PA. A video-game-based cognitive training for breast cancer survivors with cognitive impairment: a prospective randomized pilot trial. Breast 2020; 53: 23–32. | | **Ineligible outcome** |
| **42** | Höchsmann C, Infanger D, Klenk C, Königstein K, Walz SP, Schmidt-Trucksäss A. Effectiveness of a behaviour change technique-based smartphone game to improve intrinsic motivation and physical activity adherence in patients with type 2 diabetes: a randomised controlled trial. JMIR Serious Games 2019; 7: e11444. | | **Ineligible outcome** |
| **43** | Kerfoot BP, Gagnon DR, McMahon GT, Orlander JD, Kurgansky KE, Conlin PR. A team-based online game improves blood glucose control in veterans with type 2 diabetes: a randomized controlled trial. Diabetes Care 2017; 40: 1218–25. | | **Ineligible outcome** |
| **44** | Janssen A, Boster A, Lee H, Patterson B, Prakash RS. The effects of video-game training on broad cognitive transfer in multiple sclerosis: a pilot randomized controlled trial. J Clin Exp Neuropsychol 2015; 37: 285–302. | | **Ineligible outcome** |
| **45** | Bartholomew LK, Gold RS, Parcel GS, Czyzewski DI, Sockrider MM, Fernandez M, Shegog R, Swank P. Watch, Discover, Think, and Act: evaluation of computer-assisted instruction to improve asthma self-management in inner-city children. Patient Educ Couns 2000; 39: 269–80. | | Ineligible study design |
| **46** | Blanson Henkemans OA, Bierman BP, Janssen J, Neerincx MA, Looije R, van der Bosch H, van der Giessen JA. Using a robot to personalise health education for children with diabetes type 1: a pilot study. Patient Educ Couns 2013; 92: 174–81. | | Ineligible study design |
| **47** | Stapinski LA, Reda B, Newton NC, Lawler S, Rodriguez D, Chapman C, Teesson M. Development and evaluation of ‘Pure Rush’: an online serious game for drug education. Drug Alcohol Rev 2018; 37 (Suppl 1): S420–8. | | Ineligible study design |
| **48** | Tamashiro LMC, Fonseca LMM. Development of a serious game for learning about safe sex and contraception in adolescence. Rev Lat Am Enfermagem 2024; 32: e4182. | | Ineligible study design |
| **49** | Kerfoot BP, Baker H. An online spaced-education game for global continuing medical education: a randomized trial. Ann Surg 2012; 256: 33–8. | | Ineligible study design |
| **Study Identified from Relevant Systematic Reviews and Meta-Analysis but Excluded with Reasons** | | | |
| **No.** | Identified from: (Author & year) | Reference | **Reports not retrieved** |
| **1** | Montagni et al., 2020 | Bertozzi E, Krilov L, Walker D. Successful game development partnerships between academics and physicians: Two case studies. Int J Gaming Comput Simulations. 2013; 5(3):39-53. doi: 10.4018/jgcms.2013070107 | Ineligible intervention |
| **2** | Charlier et al., 2016 | Kumar VS, Wentzell KJ, Mikkelsen T, Pentland A, Laffel LM. The DAILY (Daily Automated Intensive Log for Youth) trial: a wireless, portable system to improve adherence and glycemic control in youth with diabetes. Diabetes Technol Ther. 2004;6(4):445–453. | Ineligible intervention |
| **3** | Charlier et al., 2016 | McPherson AC, Glazebrook C, Forster D, James C, Smyth A. A randomized, controlled trial of an interactive educational computer package for children with asthma. Pediatrics. 2006;117(4):1046–1054. | Ineligible intervention |
| **4** | Putri et al., 2025 | Scull TM, Dodson CV, Geller JG, Reeder LC, Stump KN. A media literacy education approach to high school sexual health education: immediate effects of Media Aware on adolescents’ media, sexual health, and communication outcomes. J Youth Adolesc. 2022;51(4):708–723. doi:10.1007/s10964-021-01567-0 | Ineligible intervention |
| **5** | Putri et al., 2025 | Alekhya G, Parida SP, Giri PP, Begum J, Patra S, Sahu DP. Effectiveness of school-based sexual and reproductive health education among adolescent girls in urban areas of Odisha, India: a cluster randomized trial. Reprod Health. 2023;20(1):105. doi:10.1186/s12978-023-01643-7 | Ineligible intervention |
| **6** | Putri et al., 2025 | Martínez-García G, Ewing AC, Olugbade Y, DiClemente RJ, Kourtis AP. Crush: a randomized trial to evaluate the impact of a mobile health app on adolescent sexual health. J Adolesc Health. 2023;72(2):287–294. doi:10.1016/j.jadohealth.2022.09.019 | Ineligible intervention |
| **7** | Putri et al., 2025 | Wendt SJ, Nakamoto J, MacDougall P, Petrosino A. Impacts of Healthy U: a cluster-randomized control trial of a sexual health education app developed for justice-involved male youth. Child Youth Serv Rev. 2022;139:106555. | Ineligible intervention |
| **8** | Putri et al., 2025 | Perez-Lu JE, Guerrero F, Cárcamo CP, et al. The ARMADILLO text message intervention to improve the sexual and reproductive health knowledge of adolescents in Peru: results of a randomized controlled trial. PLoS One. 2022;17(2):e0262986. | Ineligible intervention |
| **9** | Putri et al., 2025 | Millanzi WC, Kibusi SM, Osaki KM. Effect of integrated reproductive health lesson materials in a problem-based pedagogy on soft skills for safe sexual behaviour among adolescents: a school-based randomized controlled trial in Tanzania. PLoS One. 2022;17(2):e0263431. | Ineligible intervention |
| **10** | Putri et al., 2025 | Guilamo-Ramos V, Benzekri A, Thimm-Kaiser M, et al. A triadic intervention for adolescent sexual health: a randomized clinical trial. Pediatrics. 2020;145(5):e20192808. doi:10.1542/peds.2019-2808 | Ineligible intervention |
| **11** | Putri et al., 2025 | Wirsiy FS, Atuhaire C, Ngonzi J, Cumber SN. A randomized controlled trial on mobile phone text messaging to improve sexo-reproductive health among adolescent girls in Cameroon. Contracept Reprod Med. 2022;7(1):12. doi:10.1186/s40834-022-00180-1 | Ineligible intervention |
| **12** | Putri et al., 2025 | Yakubu I, Garmaroudi G, Sadeghi R, Tol A, Yekaninejad MS, Yidana A. Assessing the impact of an educational intervention program on sexual abstinence based on the health belief model amongst adolescent girls in Northern Ghana: a cluster randomised control trial. Reprod Health. 2019;16(1):124. doi:10.1186/s12978-019-0784-8 | Ineligible intervention |
| **13** | Putri et al., 2025 | Tu YC, Lin YJ, Fan LW, Tsai TI, Wang HH. Effects of multimedia framed messages on human papillomavirus prevention among adolescents. West J Nurs Res. 2019;41(1):58–77. doi:10.1177/0193945918763873 | Ineligible intervention |
| **14** | Putri et al., 2025 | Pakarinen M, Kylmä J, Helminen M, Suominen T. Attitudes, knowledge and sexual behavior among Finnish adolescents before and after an intervention. Health Promot Int. 2020;35(4):821–830. doi:10.1093/heapro/daz074 | Ineligible intervention |
| **15** | Putri et al., 2025 | Boti Sidamo N, Hussen S, Shegaze Shimbre M, et al. Effectiveness of curriculum-based sexual and reproductive health education on healthy sexual behaviors among year one students at Arba Minch University: a quasi-experimental study. PLoS One. 2023;18(10):e0288582. doi:10.1371/journal.pone.0288582 | Ineligible intervention |
| **16** | Putri et al., 2025 | Thongkorn A, Chaimongkol N. Effectiveness of a school-based pregnancy prevention intervention for female teenagers and their boyfriends: a randomized controlled trial. J Adolesc Health. 2023;73(2):237–243. doi:10.1016/j.jadohealth.2023.03.002 | Ineligible intervention |
| **17** | Nørlev J., 2022 | Peters R, Oleari E, Sardu F, Neerincx MA. Usability of the PAL objectives dashboard for children’s diabetes self-management education. ICSLT 2019 Proceedings of the 5th International Conference on e-Society, e-Learning and e-Technologies. Vienna: ACM; 2019:22–28. | Ineligible intervention |
| **18** | **Montagni et al., 2020** | **Böhm R, Betsch C, Korn L.** Selfish-rational non-vaccination: Experimental evidence from an interactive vaccination game. J Econ Behav Organ. 2016; 131:183-195. | Ineligible outcome |
| **19** | Charlier et al., 2016 | Homer C, Susskind O, Alpert HR, et al. An evaluation of an innovative multimedia educational software program for asthma management: report of a randomized, controlled trial. Pediatrics. 2000;106(1 Pt 2):210–215. | Ineligible outcome |
| **20** | Charlier et al., 2016 | Huss K, Winkelstein M, Nanda J, Naumann PL, Sloand ED, Huss RW. Computer game for inner-city children does not improve asthma outcomes. J Pediatr Health Care. 2003;17(2):72–78. | Ineligible outcome |
| **21** | Nørlev J., 2022 | **Ebrahimpour F, Najafi M, Sadeghi N.** The design and development of a computer game on insulin injection. Electron Physician. 2014;6(2):845–855. | Ineligible outcome |
| **22** | Nørlev J., 2022 | **Glasemann M, Kanstrup AM, Ryberg T.** Making chocolate-covered broccoli: designing a mobile learning game about food for young people with diabetes. DIS ‘10 Proceedings of the 8th ACM Conference on Designing Interactive Systems. Aarhus, Denmark: ACM; 2010:262–271. | Ineligible outcome |
| **23** | Nørlev J., 2022 | **Brox E, Hirche J, Evertsen G, Yliräisänen-Seppänen P, Bomark P.** User centric social diabetes game design for children. MindTrek ‘12 Proceedings of the 16th International Academic MindTrek Conference. Tampere, Finland: ACM; 2012:291–293. | Ineligible outcome |
| **24** | Nørlev J., 2022 | **Moosa AM, Al-Maadeed N, AlJa’am JM.** A simple health-based game for children. ICCA 2018: International Conference on Computer and Applications. Beirut: IEEE; 2018:309–312. | Ineligible outcome |
| **25** | Montagni et al., 2020 | Eley CV, Young VL, Hayes CV, Verlander NQ, McNulty CAM. Young people’s knowledge of antibiotics and vaccinations and increasing this knowledge through gaming: Mixed-methods study using e-Bug. JMIR Serious Games. 2019; 7(1):e10915. | Ineligible study design |
| **26** | Montagni et al., 2020 | Cates JR, Fuemmeler BF, Diehl SJ, Stockton LL, Porter J, Ihekweazu C, et al. Developing a serious videogame for preteens to motivate HPV vaccination decision making: Land of Secret Gardens. Games Health J. 2018; 7(1):51-66. doi: 10.1089/g4h.2017.0002 | Ineligible study design |
| **27** | Montagni et al., 2020 | Darville G, Anderson-Lewis C, Stellefson M, Lee Y, MacInnes J, Pigg RM, et al. Customization of avatars in a HPV digital gaming intervention for college-age males: An experimental study. Simulation & Gaming. 2018; 49(5):515-537. | Ineligible study design |
| **28** | Montagni et al., 2020 | Ruiz-López T, Sen S, Jakobsen E, Tropé A, Castle PE, Hansen BT, et al. FightHPV: Design and evaluation of a mobile game to raise awareness about human papillomavirus and nudge people to take action against cervical cancer. JMIR Serious Games. 2019; 7(2):e8540. doi: 10.2196/games.8540 | Ineligible study design |
| **29** | Charlier et al., 2016 | Bartholomew LK, Gold RS, Parcel GS, et al. Watch, Discover, Think, and Act: evaluation of computer-assisted instruction to improve asthma self-management in inner-city children. Patient Educ Couns. 2000;39(2–3):269–280. | Ineligible study design |
| **30** | Nørlev J., 2022 | Calle-Bustos A, Juan MC, García-García I, Abad F. An augmented reality game to support therapeutic education for children with diabetes. PLoS One. 2017;12(9):e0184645. | Ineligible study design |
| **31** | Nørlev J., 2022 | Thompson D, Baranowski T, Buday R, Arch B. Conceptual model for the design of a serious video game promoting self-management among youth with type 1 diabetes. J Diabetes Sci Technol. 2010;4(3):744–749. | Ineligible study design |
| **32** | Nørlev J., 2022 | Lieberman DA. Management of chronic pediatric diseases with interactive health games: theory and research findings. J Ambul Care Manag. 2001;24(1):26–38. | Ineligible study design |
| **33** | Nørlev J., 2022 | Rønningen IC, Årsand E, Hartvigsen G. Exploring in-game reward mechanisms in Diaquarium: a serious game for children with type 1 diabetes. In: Rojas I, Ortuño F, eds. IWBBIO 2018: Bioinformatics and Biomedical Engineering. Lecture Notes in Computer Science. Vol 10814. Springer; 2018:443–455. | Ineligible study design |
| **34** | Nørlev J., 2022 | Baghaei N, Nandigam D, Casey J, Direito A, Maddison R. Diabetic Mario: designing and evaluating mobile games for diabetes education. Games Health J. 2016;5(4):270–278. | Ineligible study design |
| **35** | Nørlev J., 2022 | Pesare E, Roselli T, Rossano V. Intelligent agent and virtual game to support education in e-health. DMS 2015: 21st International Conference on Distributed Multimedia Systems. Vancouver, Canada: KSI Research; 2015. | Ineligible study design |
| **36** | Nørlev J., 2022 | Lauritzen J, Årsand E, Horsch A, et al. Social media and games as self-management tools for children and adolescents with type 1 diabetes mellitus. In: Conchon E, Correia C, Fred A, Gamboa H, eds. HEALTHINF 2012: International Conference on Health Informatics. Algarve, Portugal: SciTePress; 2012:459–466. | Ineligible study design |
| **37** | Nørlev J., 2022 | Bomark P, Evertsen G, Brox E, Hirche J, Yliräisänen-Seppänen P. A prototype social learning platform for children with diabetes type 1. MindTrek ‘12 Proceedings of the 16th International Academic MindTrek Conference. Tampere, Finland: ACM; 2012:211–213. | Ineligible study design |
| **38** | Nørlev J., 2022 | D’Aprile G, Ligorio MB, Severino AU. How serious games for health enhance empowerment-related patient education: the impact of gender. Technol Knowl Learn. 2019;24(3):325–340. | Ineligible study design |
| **39** | Nørlev J., 2022 | Sparapani VC, Fels S, Kamal N, Nascimento LC. Conceptual framework for designing video games for children with type 1 diabetes. Rev Lat Am Enfermagem. 2019;27:e3090. | Ineligible study design |
| **40** | Nørlev J., 2022 | Kamel Boulos MN, Gammon S, Dixon MC, et al. Digital games for type 1 and type 2 diabetes: underpinning theory with three illustrative examples. JMIR Serious Games. 2015;3(1):e3. | Ineligible study design |
| **41** | Montagni et al., 2020 | Fadda M, Galimberti E, Fiordelli M, Romanò L, Zanetti A, Schulz PJ. Effectiveness of a smartphone app to increase parents’ knowledge and empowerment in the MMR vaccination decision: A randomized controlled trial. Hum Vaccin Immunother. 2017; 13(11):2512-2521. doi: 10.1080/21645515.2017.1360456 | duplicated |
| **42** | Charlier et al., 2016 | Shegog R, Bartholomew LK, Parcel GS, Sockrider MM, Massey M, Abramson SL. Impact of a computer-assisted education program on factors related to asthma self-management behavior. J Am Med Inform Assoc. 2001;8(1):49–61. | duplicated |
| **43** | Charlier et al., 2016 | Kato PM, Cole SW, Bradlyn AS, Pollock BH. A video game improves behavioral outcomes in adolescents and young adults with cancer: a randomized trial. Pediatrics. 2008;122(2):e305–e317. | duplicated |
| **44** | Charlier et al., 2016 | Rubin DH, Leventhal JM, Sadock RT, et al. Educational intervention by computer in childhood asthma—a randomized clinical trial testing the use of a new teaching intervention in childhood asthma. Pediatrics. 1986;77(1):1–10. | Published before 2000 |
| **45** | Charlier et al., 2016 | Brown SJ, Lieberman DA, Germeny BA, Fan YC, Wilson DM, Pasta DJ. Educational video game for juvenile diabetes: results of a controlled trial. Med Inform (Lond). 1997;22(1):77–89. | Published before 2000 |
| **46** | Nørlev J., 2022 | **Lieberman DA.** Interactive video games for health promotion: effects on knowledge, self-efficacy, social support, and health. In: Street RL, Gold WR, Manning T, eds. Health Promotion and Interactive Technology: Theoretical Applications and Future Directions. Mahwah, NJ: Lawrence Erlbaum Associates; 1997:103–120. | Published before 2000 |
| **47** | Nørlev J., 2022 | **Brown SJ, Lieberman DA, Germeny BA, Fan YC, Wilson DM, Pasta DJ.** Educational video game for juvenile diabetes: results of a controlled trial. Med Inform. 1997;22(1):77–89. | Published before 2000 |
