# Supplementary material for: Effectiveness of Digital Serious Games on Knowledge and Attitudes in Public Health Education: Systematic Review and Bayesian Network Meta-Analysis of Randomized Controlled Trials
Source: J Med Internet Res. 2026 Apr 24;28:e89281. doi: 10.2196/89281 (PMC13108840; doi:10.2196/89281)
Supplement: Multimedia Appendix 6 [file jmir-v28-e89281-s006.docx]

**Multimedia Appendix 7.** Design and Characteristics of Included Digital Serious Games

| **Study ID** | **Game Name** | **Developer / Institution** | **Interaction Features** | **Purpose** |
| --- | --- | --- | --- | --- |
| **Aljafari et al., 2017** | *Dental Detective* | King’s College London Dental Institute | Interactive pictorial quiz with immediate feedback; replayable; parent–child co-play. | Improve children’s dietary and oral health knowledge. |
| **Aljafari et al., 2022** |  | University of Jordan & King’s College London | Interactive oral-health education game adapted to Arabic culture, featuring brushing, diet, and dentist missions with reward feedback. | Improve dietary knowledge and oral health awareness in Jordanian primary school children. |
| **Fadda et al., 2017** | *MorbiQuiz* | University of Lugano; University of Milan | Gamified daily quiz, video narratives, progress tracking | To improve parents’ knowledge and empowerment in MMR vaccination decisions |
| **Haruna et al., 2018** | My Future Starts Today | Universiti Putra Malaysia | 3D storytelling game with avatars, role-play, and quizzes delivered in classroom sessions. | Improve adolescents’ sexual health knowledge and attitudes. |
| **Henkemans et al., 2017** | Diabetes Education Game | TNO Research Group & University of Twente | Humanoid robot (NAO) provided verbal feedback and encouragement during gameplay across 3 sessions. | Improve diabetes self-management knowledge in children with type 1 diabetes. |
| **Kumar RS et al., 2022** | *Visual Interactive Game* | Sree Balaji Dental College, India | Computer-based animated oral hygiene game with interactive questions. | Improve adolescents’ oral hygiene knowledge and practices. |
| **Koohmareh et al., 2021** | *Amoo* | Ahvaz Jundishapur University of Medical Sciences | Two-episode food selection game teaching calorie and glycemic-index concepts with intelligent feedback (15 min daily × 6 weeks). | Improve dietary knowledge and glycemic-index awareness in adults with type 2 diabetes. |
| **Pouls et al., 2022** | *Medi & Seintje* | Game Solutions Lab & Sint Maartenskliniek (with AbbVie Inc.) | Puzzle-based game (crossword, sudoku, word search, tangram) with behavioural tasks modifying implicit attitudes toward medication. | Improve adherence to DMARDs in patients with rheumatoid arthritis. |
| **Tang et al., 2022** | *AIDS Fighter: Health Defense* | AIDS Healthcare Foundation China Office & China CDC | Role-playing, shooting and quiz-based HIV/AIDS education game with narrative “health defence” missions. | Improve adolescents’ HIV/AIDS knowledge and attitudes. |
| **Whiteley et al., 2018** | *BattleViro* | University of Connecticut (CHIP) | 3D role-playing adherence game simulating HIV management, combining behavioural feedback and medication tracking. | Improve HIV-related knowledge and health attitudes among youth with HIV. |
| **Carcioppolo et al., 2022** | *Melanoma Education Game* | University of Miami | Short interactive game featuring melanoma image identification tasks and feedback-based learning. | Improve adults’ knowledge and attitudes toward melanoma identification and prevention. |
| **Espinosa-Curiel et al., 2022** | *HelperFriend* | Centro de Investigación Científica y de Educación Superior de Ensenada (CICESE), Mexico | Video-based serious game integrating animated storytelling with motion-controlled activities via Kinect; includes modules on physical activity, healthy eating, and socio-emotional wellness; uses visual feedback and rewards to reinforce correct behaviors. | To improve children’s knowledge, attitudes, and behavioral intentions regarding healthy lifestyle habits. |
| **Ghadam et al., 2022** | *Digital game-based nutrition education intervention* | Shiraz University of Medical Sciences, Iran | Interactive level-based game comprising seven stages; each stage contained nutrition-related content on anemia prevention, released biweekly with reward feedback and quiz elements. | To enhance adolescents’ knowledge, attitudes, and practices regarding iron-rich nutrition and anemia prevention. |
| **Huang et al., 2025** | *Gamified medication adherence program* | Zhejiang University School of Pharmacy, China | Gamified adherence system with points, rankings, educational reminders, and real-time inhaler feedback. | To improve COPD patients’ medication adherence and disease-related knowledge. |
| **Joubert et al., 2016** | L’Affaire Birman (Mr. Birman’s File) | University Hospital of Caen & Zippyware Company, France | 3D interactive adventure simulating flexible insulin therapy; glycemic simulator with feedback on carb counting and insulin dosing; situational problem-solving tasks. | To improve knowledge of carbohydrate quantification and insulin adjustment among children with Type 1 diabetes. |
| **Kato et al., 2008** | Re-Mission | HopeLab Foundation, Palo Alto, USA | 3D shooter-style game where players control a nanobot to destroy cancer cells and manage treatment side effects; provides narrative feedback and performance tracking. | To improve cancer-related knowledge, self-efficacy, and adherence among adolescents and young adults with cancer. |
| **Koniou et al., 2025** | Virtual Reality Autism Awareness Simulation | Université de Montréal, Canada | Immersive 8-min perspective-taking simulation replicating sensory overload and communication barriers faced by autistic individuals; followed by an educational video. | To raise awareness, improve autism-related knowledge and attitudes, and enhance empathy and openness toward autistic individuals. |
| **Kumar et al., 2004** | *DiaBetNet* | MIT Media Lab & Dimagi Inc., USA | Diabetes management game integrating glucose, insulin, and carbohydrate data; data uploaded wirelessly to server. | To improve diabetes knowledge and glucose monitoring in youth with diabetes. |
| **Liu et al., 2024** | *AR Serious Game for Optical Science* | School of Design, Shanghai Jiao Tong University | 3D augmented reality interaction using Unity and Vuforia on iPad; includes animation, voice-over, and interactive experiments. | To improve children’s understanding of optical science concepts and learning motivation. |
| **Mack et al., 2020** | *Kids Obesity Prevention (KOP)* | University Hospital Tübingen & University of Tübingen (Germany) | Motion-controlled 3D PC + tablet tasks on nutrition, physical activity and stress coping; applies Kinect and touch interaction. | To improve children’s knowledge of nutrition and stress coping for obesity prevention. |
| **Maddison et al., 2022** | *Ari and Friends* / *Diabetic Jumper* | National Institute for Health Innovation (University of Auckland) & Massey University | 2D side-scrolling and jumping games with embedded quiz questions on diet and exercise; 15 min per session, 4 weeks total. | To evaluate feasibility and preliminary effects of mobile serious games on diabetes and healthy lifestyle knowledge in at-risk youth. |
| **Maganty et al., 2018** | Tapamole | Mayo Clinic Arizona | Online visual selection game identifying melanoma images from benign lesions; 25 MM + 60 benign images; playable on PC/tablet/smartphone; single session. | To assess the effectiveness and enjoyment of a web-based game in improving public recognition and confidence regarding melanoma features. |
| **Nazmi et al., 2025** | D-Gamify Puberty Health Platform | Babol University of Medical Sciences & Danosh Company (Iran) | Web-based gamified learning platform with points, leaderboards and badges; weekly interactive quizzes and key-point tasks over 4 weeks. | To enhance pubertal health knowledge and practice among adolescent girls through a 4-week online gamified education program. |
| **Nowak et al., 2020** | *VR Vaccine Education Experience* | Stanford Virtual Human Interaction Lab (VHIL) | 360° immersive simulation showing influenza spread and herd immunity; participants observe infection chain in community context via headset. | To evaluate whether an immersive VR simulation improves vaccination beliefs and intentions compared with standard video education. |
| **Bartholomew et al., 2000** | *Watch, Discover, Think, and Act (WDTA)* | University of Texas-Houston Health Science Center | Clinic-based stand-alone educational program with interactive animations and quizzes teaching asthma triggers and inhaler management. | To evaluate effects of a computer-assisted education program on asthma self-management knowledge and attitudes in children. |
| **Shegog et al., 2001** |  | Baylor College of Medicine & UT Health Science Center Houston | Simulation-based asthma education game with four-step self-regulation model, real-world scenarios, and animated peer-coach feedback. | Improve asthma self-management knowledge, self-efficacy, and attribution among children. |
| **Tan et al., 2022** | *Dengue Patrol* | Singapore Ministry of Health & NEA | Web-based interactive dengue prevention game where players identify mosquito breeding sites and complete vector control missions. | To evaluate the effect of a web-based serious game on public knowledge, attitudes, and practices toward dengue prevention. |
| **Beaujean et al., 2016** | *Tick Bite Game* | Dutch National Institute for Public Health and the Environment (RIVM) | Players explore virtual outdoor environments, identify ticks, and make prevention decisions (e.g., clothing choice, checking body after being outdoors) | To improve children’s knowledge and preventive behaviors toward tick bites and Lyme disease |
| **Huang et al., 2024** | *Bug Busters* | National University of Singapore, Yong Loo Lin School of Medicine | Interactive decision-making scenarios simulating antibiotic use and infection control in daily life; feedback provided after each scenario | To enhance public knowledge and awareness of appropriate antibiotic use and antimicrobial resistance prevention |
| **Beale et al., 2007** | *Re-Mission* | HopeLab, Redwood City, California, USA | Third-person 3D action game where players control a nanobot avatar inside virtual cancer patients, using chemotherapy, antibiotics, and radiation weapons to destroy cancer cells and promote self-care behaviors. | To improve adolescents’ and young adults’ cancer knowledge and encourage adherence to treatment through psychoeducational gameplay. |
| **Khalil et al., 2016** |  |  | Players control a nanobot that destroys cancer cells and infections inside a virtual body; gameplay difficulty dynamically adjusts to evoke perceived susceptibility and severity. | To enhance young adults’ awareness of cancer risk and promote information-seeking and protective health behaviors. |
| **Bloomfield et al., 2025** | *Meningococcal Immunisation Awareness, Prevention and Protection app (MIApp)* | Edith Cowan University, Amanda Young Foundation, Western Australia Department of Health, and Lotterywest | Self-directed interactive narrative; players investigate infection source through quizzes, simulations, and object collection; guided by an in-game character (“Buddy”) | To educate adolescents about invasive meningococcal disease transmission, symptoms, and vaccination as an equivalent alternative to face-to-face teaching |
| **Boomer et al., 2024** | *PlayTest!* | Play2PREVENT Lab, Yale Center for Health & Learning Games; Yale School of Medicine; McGill University; Schell Games; Digitalmill | Interactive narrative where players progress through high school scenarios, create avatars, make social decisions, and complete mini-games (e.g., “Know Sense,” “Priority Sense”) using motivational interviewing, social learning, and self-efficacy mechanics | To promote HIV testing and counseling (HTC) among adolescents by improving attitudes, intentions, knowledge, and self-efficacy toward HIV testing through theory-based gameplay |
| **Fiellin et al., 2017** | *PlayForward: Elm City Stories* | Yale Center for Health & Learning Games; play2PREVENT Lab; Digitalmill | 3D interactive story-driven adventure where players create avatars, navigate realistic social and sexual-health scenarios, and experience consequences of decisions | To reduce sexual-risk behaviours and enhance HIV/STI prevention knowledge and self-efficacy among minority adolescents |
| **Raj et al., 2025** | *Pragati Path (Progress Path)* | Indian Institute of Public Health; UNICEF India; Ministry of Health and Family Welfare | Interactive reproductive-health education game featuring quizzes, animations, and scenario-based problem solving; users complete multi-level sessions at their own pace | To enhance reproductive and sexual health knowledge, attitudes, and decision-making self-efficacy among adolescent girls in India through gamified digital learning aligned with national adolescent health programs |
| **Wang et al., 2025** | *Food Adventure Quest* | Zhejiang University; National Institute for Nutrition and Health (China CDC) | Alternate reality simulation game combining quiz-based missions, storytelling, and dietary behavior feedback; children complete multi-level nutrition challenges and receive visualized scores | To enhance nutrition knowledge and promote healthy eating behaviors among Chinese primary school students through interactive gameplay and repeated learning sessions |
| **Vandeweerdt et al., 2022** | Community Immunity VR Simulation | University of Copenhagen; ETH Zürich; IT University of Copenhagen | Immersive 3D gamified simulation where participants act as infected or uninfected avatars, navigating virtual environments with varying vaccination rates (20% vs 70%) to experience herd immunity dynamics via visual and haptic feedback | To reduce COVID-19 vaccine hesitancy by improving understanding of community immunity and collective responsibility through immersive experiential learning |
