# Supplementary material for: Effectiveness of Digital Serious Games on Knowledge and Attitudes in Public Health Education: Systematic Review and Bayesian Network Meta-Analysis of Randomized Controlled Trials
Source: J Med Internet Res. 2026 Apr 24;28:e89281. doi: 10.2196/89281 (PMC13108840; doi:10.2196/89281)

**Multimedia Appendix 8.** Risk of bias assessment for each outcome
**Supplementary Figure S1.** Risk of bias for individually randomised trials.


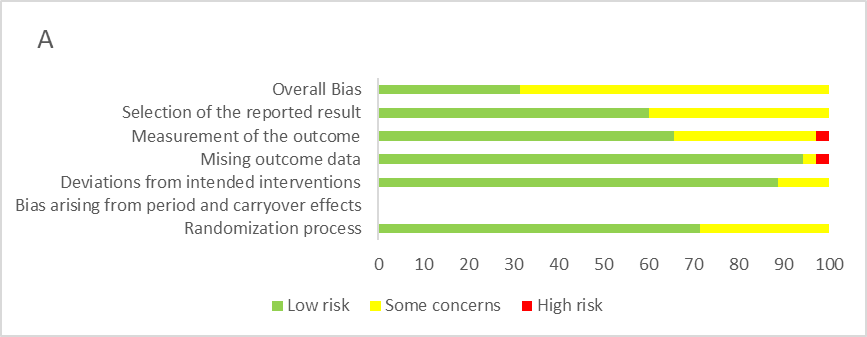


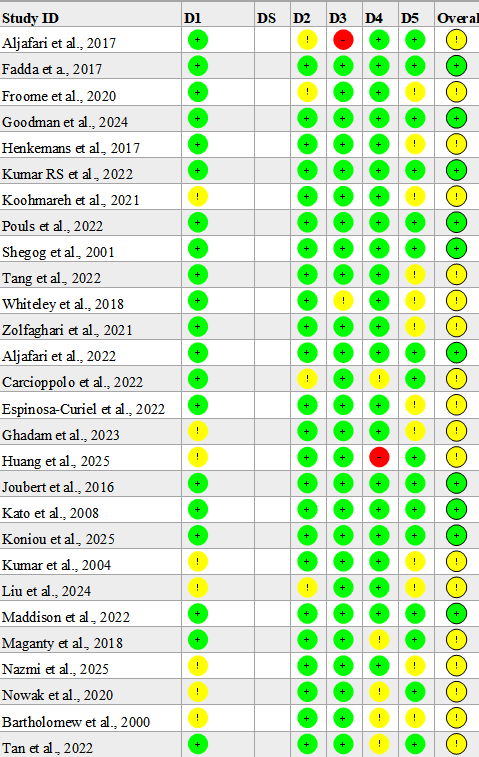


**Supplementary Figure S2.** Risk of bias for cluster randomised trials.


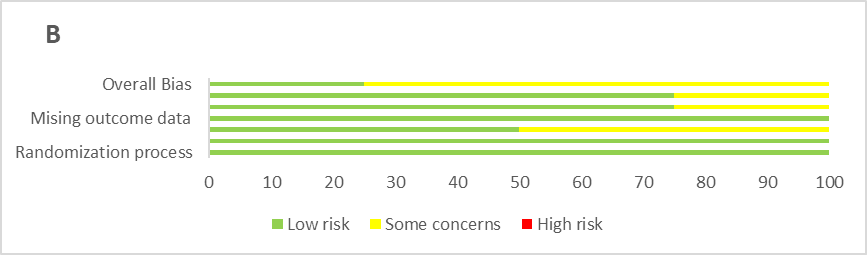


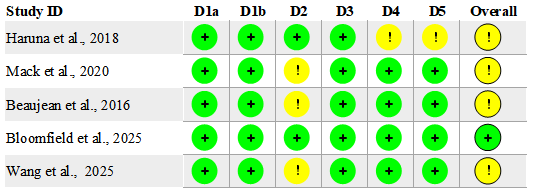

Supplement: Multimedia Appendix 7 [file jmir-v28-e89281-s007.docx]
