# Supplementary material for: Effectiveness of Digital Serious Games on Knowledge and Attitudes in Public Health Education: Systematic Review and Bayesian Network Meta-Analysis of Randomized Controlled Trials
Source: J Med Internet Res. 2026 Apr 24;28:e89281. doi: 10.2196/89281 (PMC13108840; doi:10.2196/89281)
Supplement: Multimedia Appendix 8 [file jmir-v28-e89281-s008.docx]

**Multimedia Appendix 9.** Funnel plots assessing publication bias across the included studies

**Figure S3. Funnel plot assessing publication bias in knowledge outcomes**
The vertical dashed line represents the pooled standardised mean difference (SMD) under the random-effects model; dotted lines indicate 95% confidence limits.
**
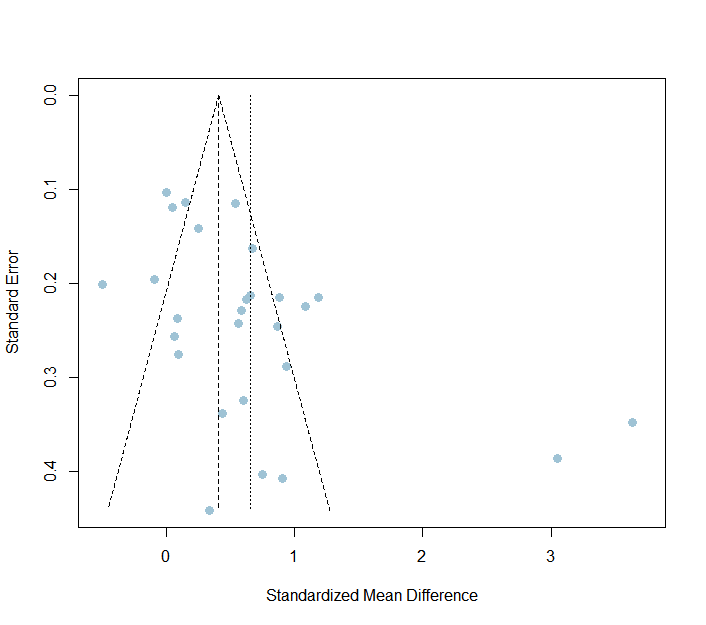
**

**Figure S4. . Funnel plot assessing publication bias in attitude outcomes**The vertical dashed line represents the pooled SMD under the random-effects model; dotted lines indicate 95% confidence limits.

**
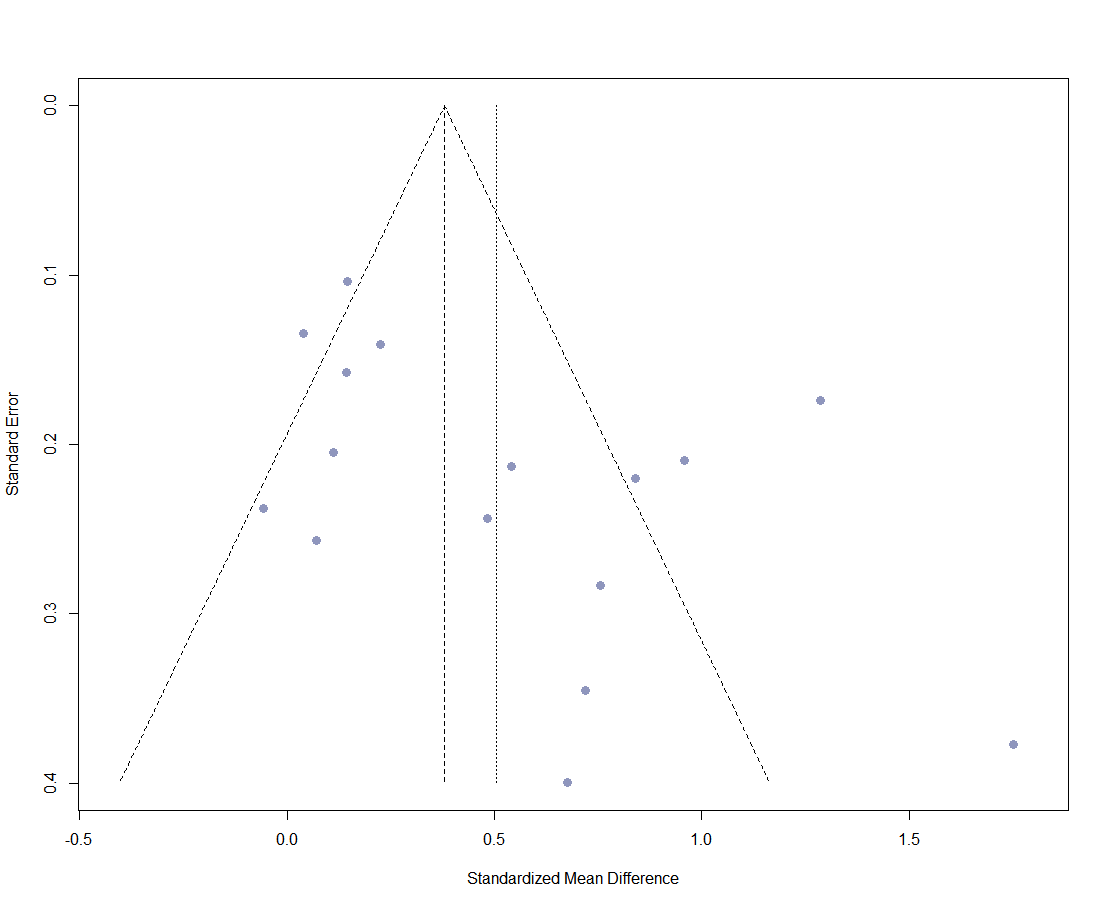
**
