# Supplementary material for: Effectiveness of Digital Serious Games on Knowledge and Attitudes in Public Health Education: Systematic Review and Bayesian Network Meta-Analysis of Randomized Controlled Trials
Source: J Med Internet Res. 2026 Apr 24;28:e89281. doi: 10.2196/89281 (PMC13108840; doi:10.2196/89281)
Supplement: Multimedia Appendix 9 [file jmir-v28-e89281-s009.docx]

**Multimedia Appendix 10**. GRADE Assessment for Knowledge and Attitude Outcomes

**Population:** Members of the general public, including adolescents and adults without a medical background. Studies involving informal caregivers were also included if the intervention aimed to enhance public understanding of disease-related knowledge or attitudes.

**Setting:** Studies published in English with no restrictions on country or intervention delivery mode. **Intervention:** Digital serious games delivered through computer, mobile, web-based, or VR/AR platforms, explicitly designed to improve knowledge or attitudes related to diseases or health conditions

**Comparison:** Conventional or web-based health education, usual care, or non-game control conditions.

**Outcomes:** Quantitative measures of change in disease-related knowledge and/or health attitudes between intervention and control groups.

| **Outcomes** | **No.** **of studies** | **Participants** | **Effect estimate  (SMD, 95% CI)** | **Overall quality of evidence†** |
| --- | --- | --- | --- | --- |
| **Knowledge** | **27** | **3093  (INT 1580; CON 1513)** | **0.66 (0.32–0.99)** | **⊕⊕⊕◯ Moderate‡** |
| **Attitude** | **16** | **1858  (INT 932; CON 926)** | **0.50 (0.27–0.76)** | **⊕⊕⊕◯ Moderate§** |

† Certainty of evidence grades:

⊕⊕⊕⊕ High – Further research is unlikely to change the confidence in the estimate of effect.

⊕⊕⊕◯ Moderate – Further research is likely to have an important impact on the confidence in the estimate of effect and may change the estimate.

⊕⊕◯◯ Low – Further research is very likely to have an important impact on the confidence in the effect estimate and is likely to change the estimate.

⊕◯◯◯ Very low – Any estimate of effect is very uncertain.

‡ The overall certainty of evidence for knowledge outcomes was downgraded by one level due to methodological concerns in randomisation and allocation concealment and by one level for potential publication bias indicated by Egger’s test (p = 0.0059). Despite high heterogeneity (I² = 89.1%), subgroup analyses explained most of the variation by intervention duration, population type, and health topic; therefore, inconsistency was not further downgraded.

§ The overall certainty of evidence for attitude outcomes was downgraded by one level for minor methodological limitations and another for small-study effects (Egger’s p = 0.0518). Although substantial heterogeneity was observed (I² = 80.7%), the direction of effects was consistent, and subgroup analyses accounted for the observed variation.
