# Supplementary material for: Effectiveness of Digital Serious Games on Knowledge and Attitudes in Public Health Education: Systematic Review and Bayesian Network Meta-Analysis of Randomized Controlled Trials
Source: J Med Internet Res. 2026 Apr 24;28:e89281. doi: 10.2196/89281 (PMC13108840; doi:10.2196/89281)
Supplement: Multimedia Appendix 10 [file jmir-v28-e89281-s010.docx]

**Multimedia Appendix 11.** Subgroup analyses of knowledge and attitude outcomes across seven moderators: intervention duration, study region, patient status, health topic, publication year, population type, and sex composition.

**Figure S5.** Forest plot of subgroup analysis by intervention duration for knowledge outcomes, comparing single-session and multi-session interventions.

*
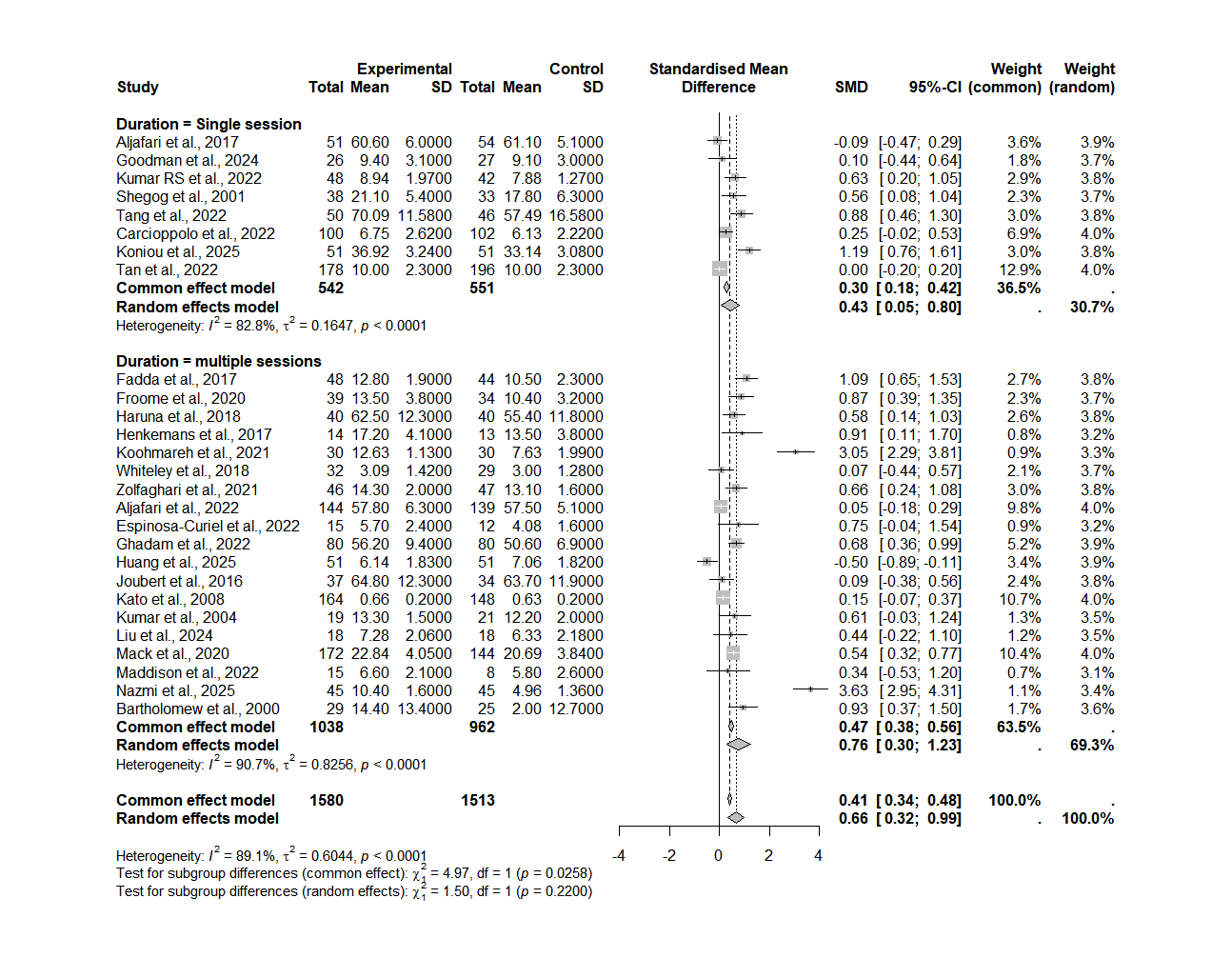
*

**Figure S6.** Forest plot of subgroup analysis by study region for knowledge outcomes, comparing effects among studies conducted in Asia, Europe, and North America. *
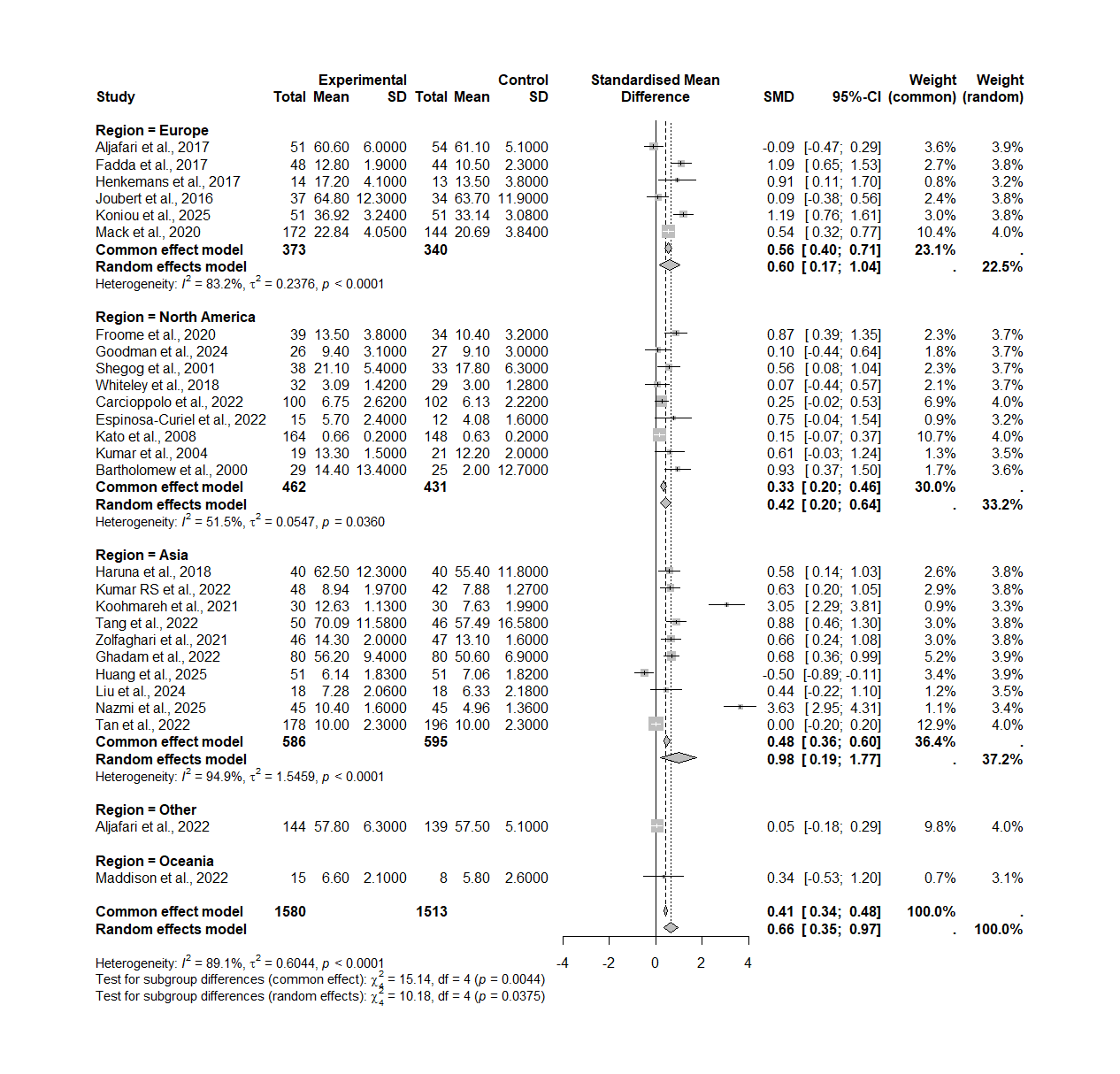
*

**Figure S7.** Forest plot of subgroup analysis by patient status for knowledge outcomes, comparing knowledge improvement between patient and non-patient populations.

*
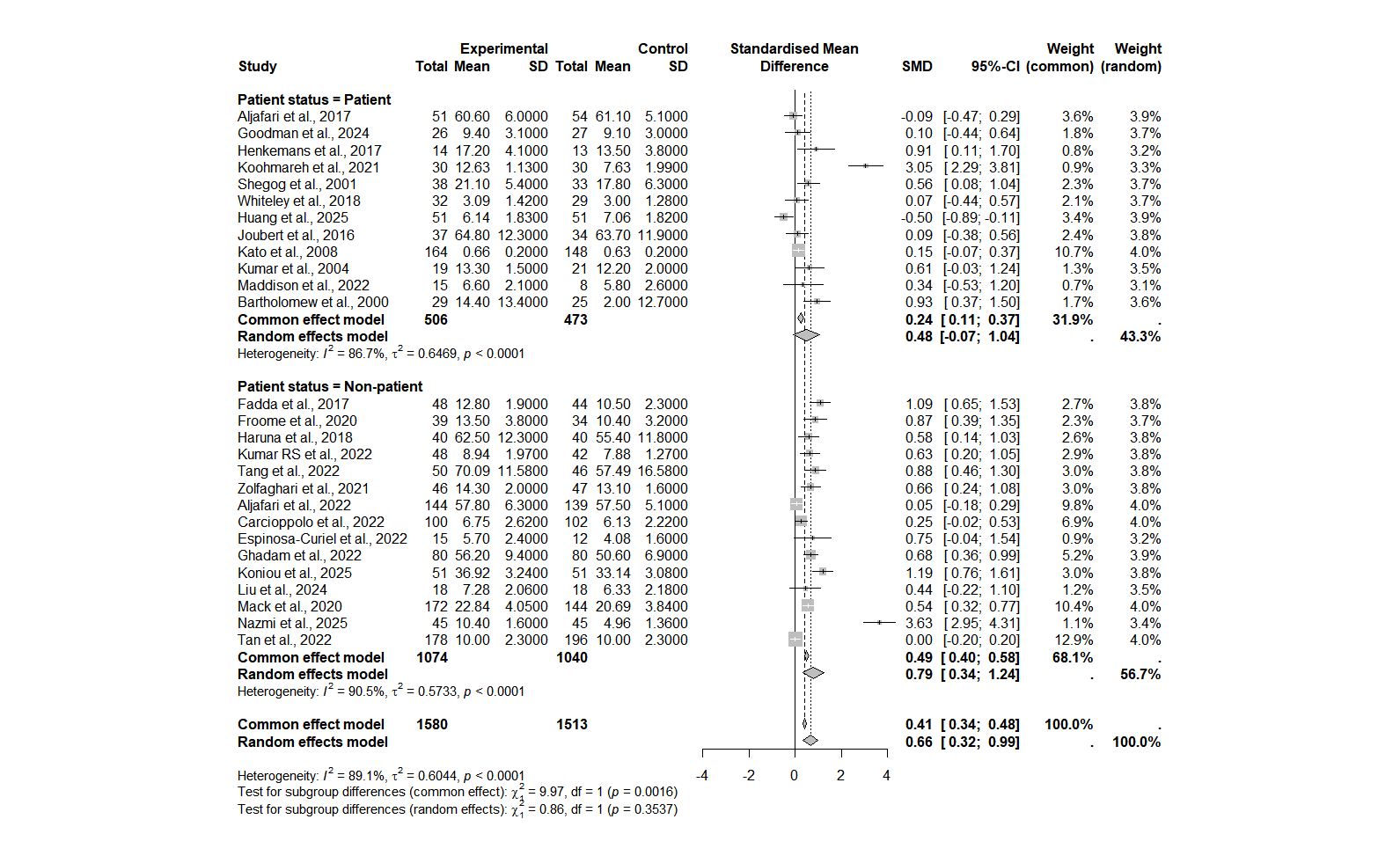
*

**Figure S8.** Forest plot of subgroup analysis by health topic for knowledge outcomes, showing effects across cancer, chronic disease, vaccination, and oral health education.

*
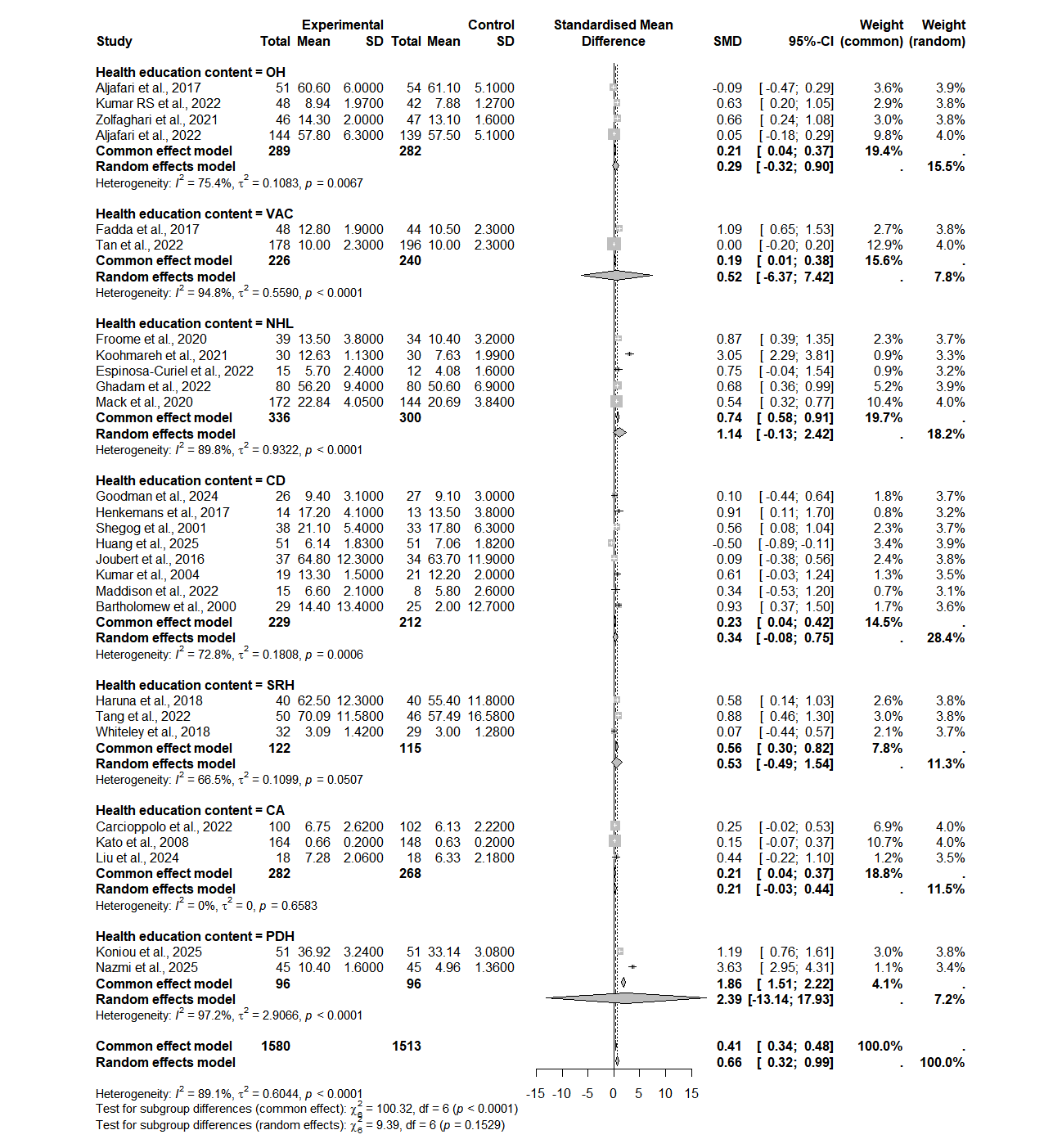
*

**Figure S9.** Forest plot of subgroup analysis by publication year for knowledge outcomes, illustrating temporal trends in knowledge improvement across studies.

*
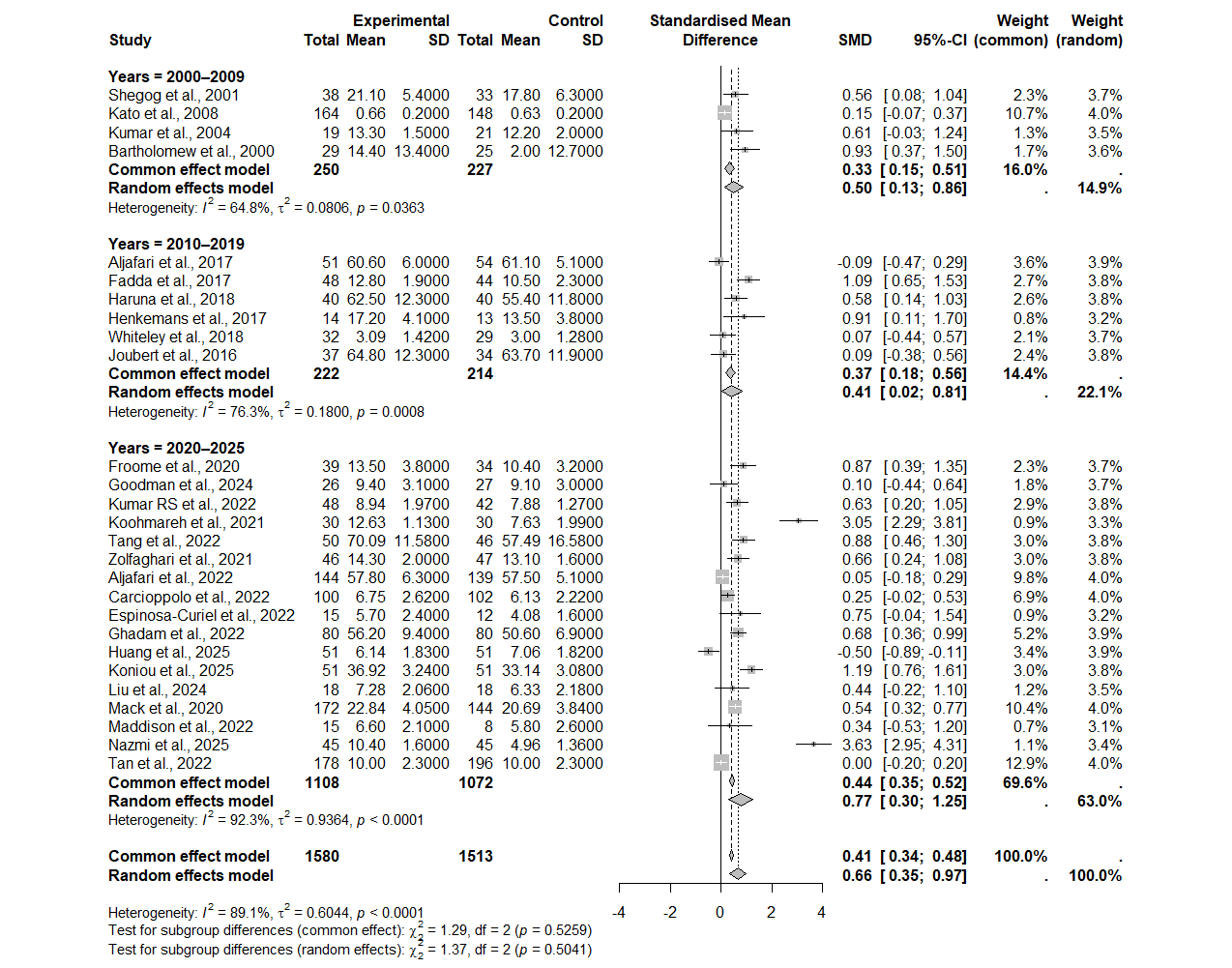
*

**Figure S10.** Forest plot of subgroup analysis by population type for knowledge outcomes, comparing effects among children, adolescents, and adults, including combined samples of children and adolescents and adolescents and adults.

*
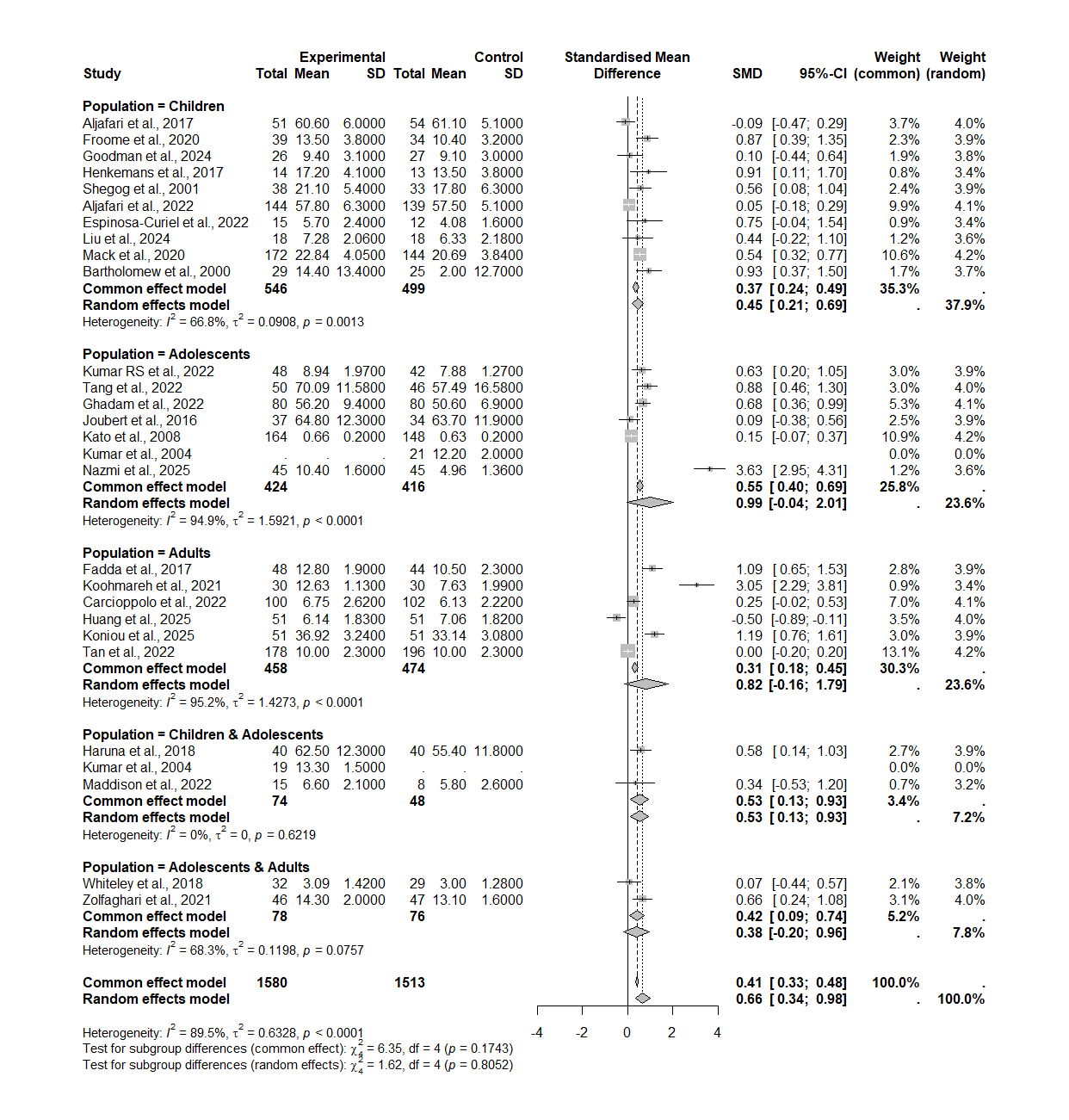
*

**Figure S11.** Forest plot of subgroup analysis by sex composition for knowledge outcomes, comparing studies with higher versus lower proportions of female participants.

*
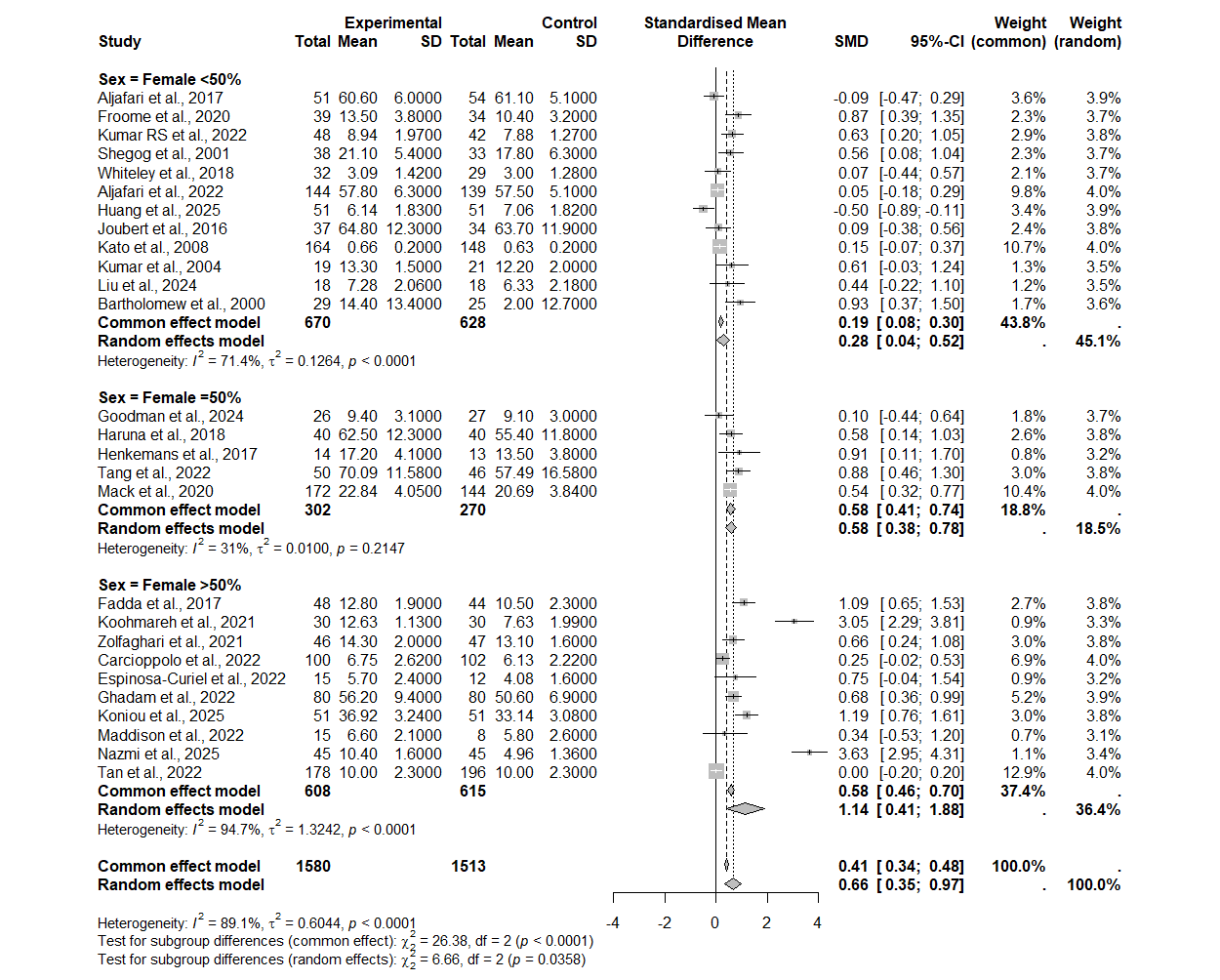
*

**Figure S12.** Forest plot of subgroup analysis by intervention duration for attitude outcomes, comparing single-session and multi-session interventions.

*
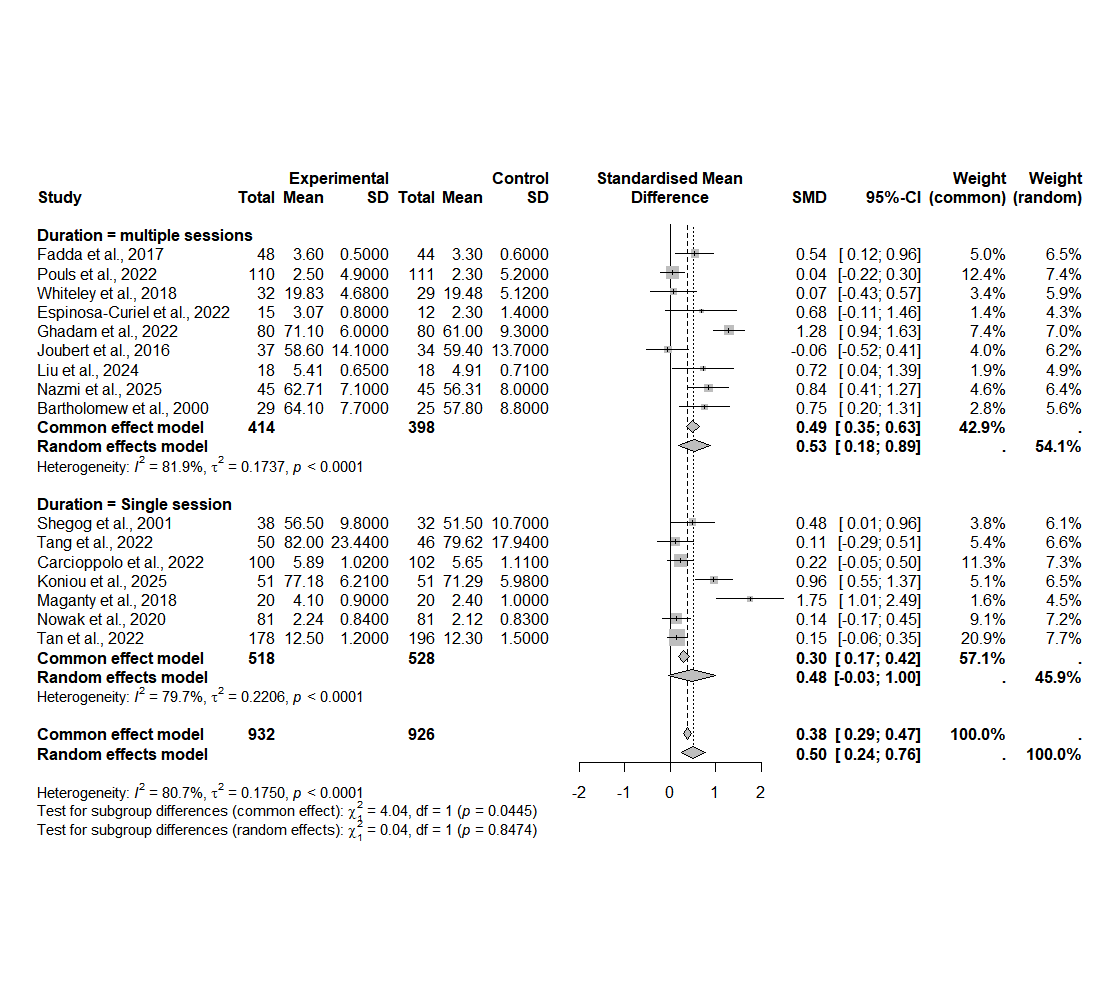
*

**Figure S13.** Forest plot of subgroup analysis by study region for attitude outcomes, comparing effects among studies conducted in Asia, Europe, and North America.

*
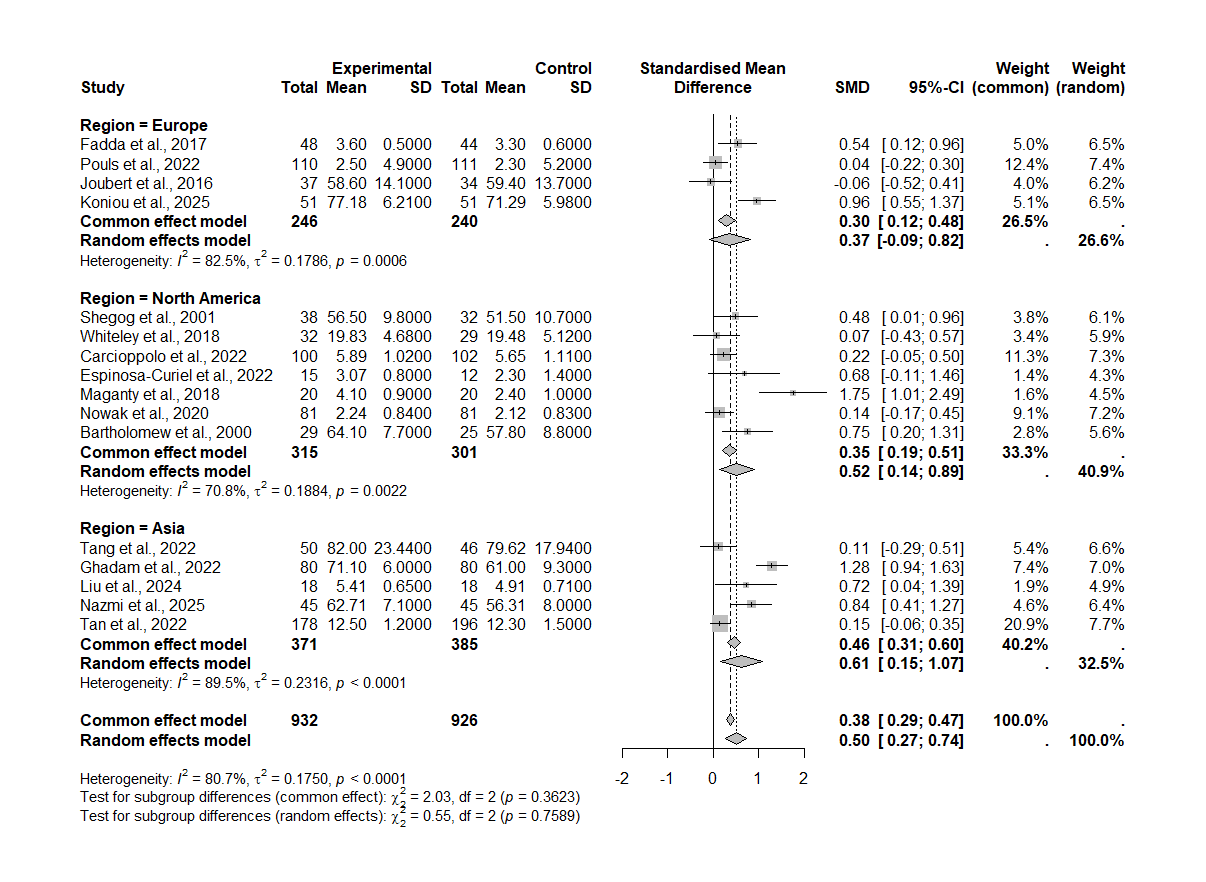
*

**Figure S14.** Forest plot of subgroup analysis by patient status for attitude outcomes, comparing attitude changes between patient and non-patient populations.

*
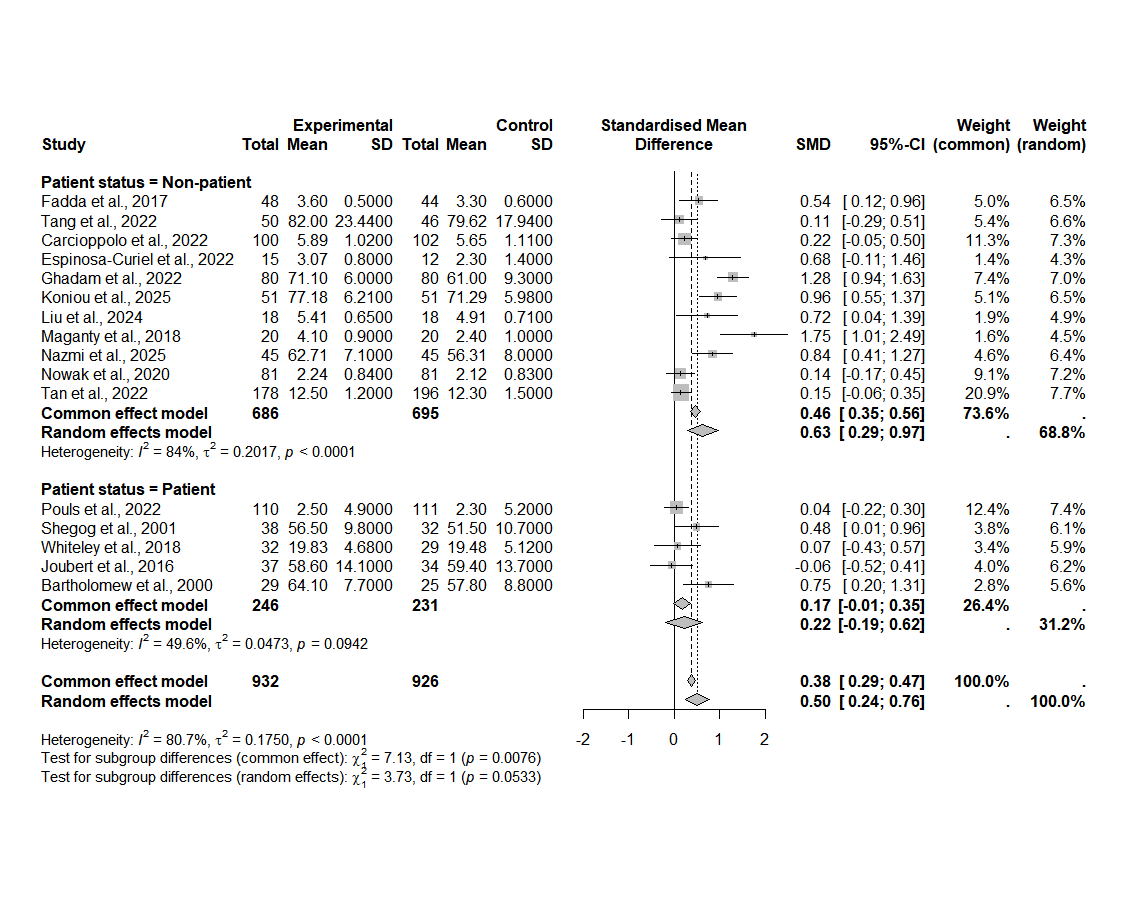
*

**Figure S15.** Forest plot of subgroup analysis by health topic for attitude outcomes, showing effects across cancer, chronic disease, vaccination, and oral health education


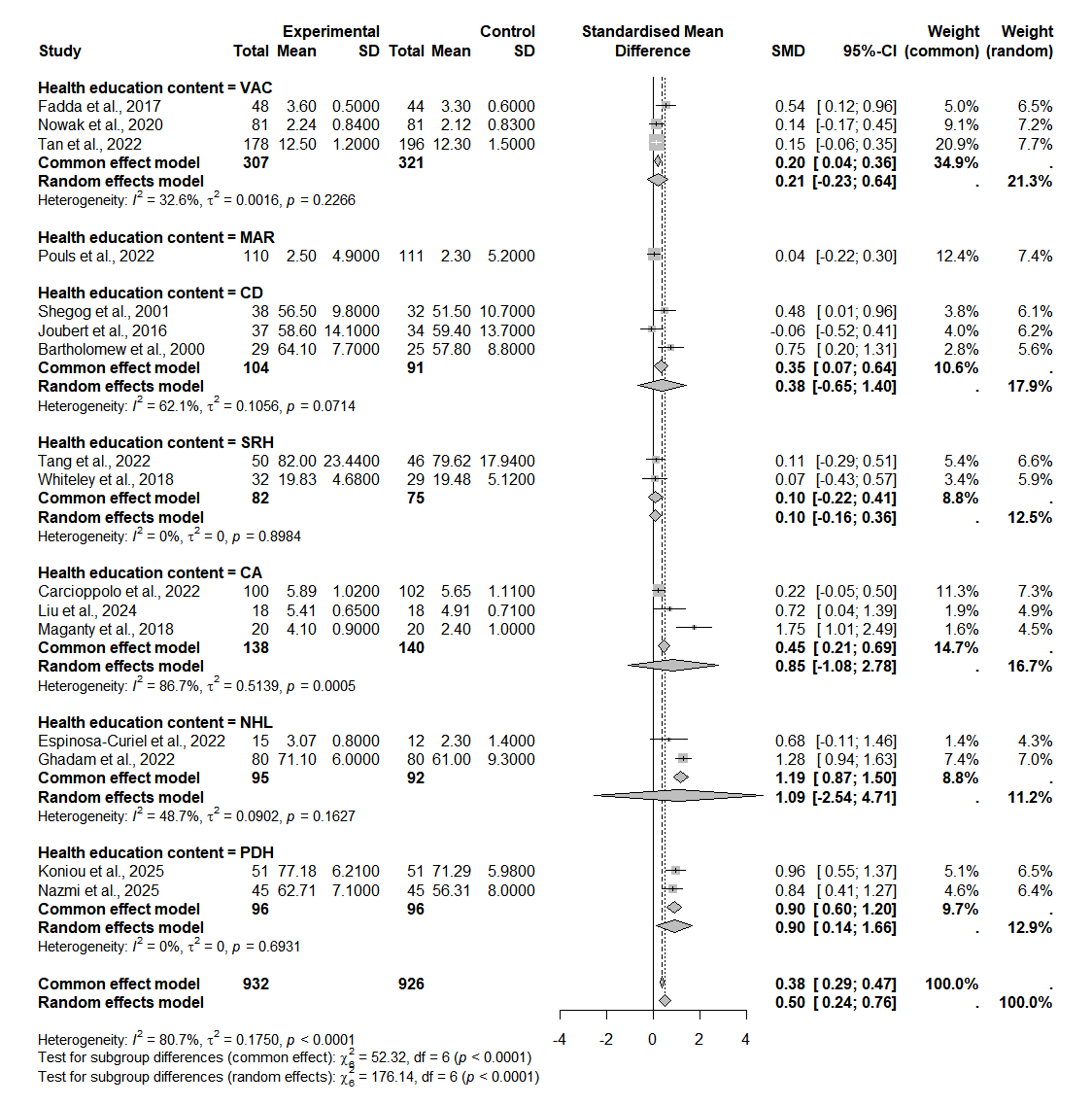


**Figure S16.** Forest plot of subgroup analysis by publication year for attitude outcomes, illustrating temporal trends in attitude improvement across studies.

*
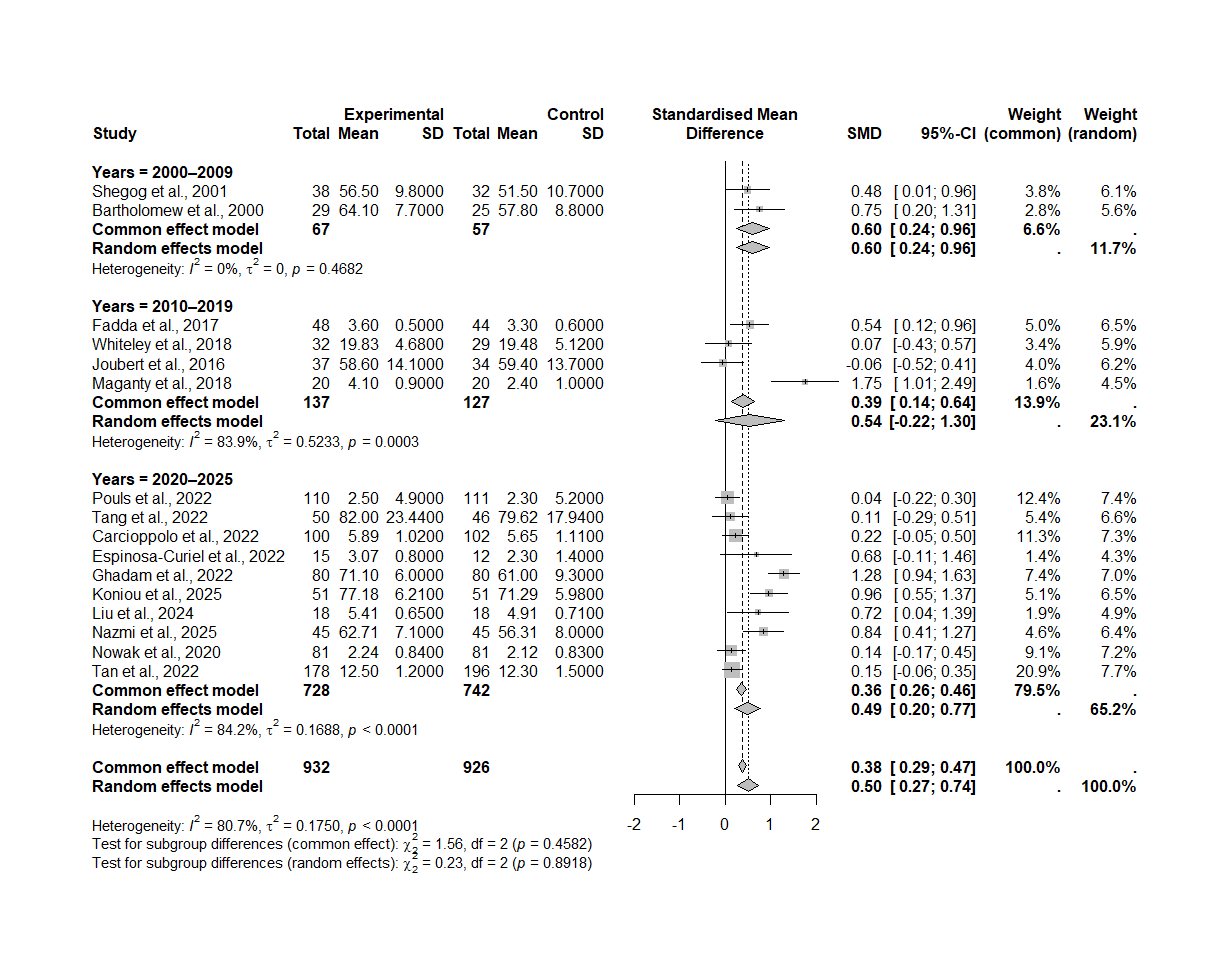
*

**Figure S17.** Forest plot of subgroup analysis by population type for attitude outcomes, comparing effects among children, adolescents, and adult.

*
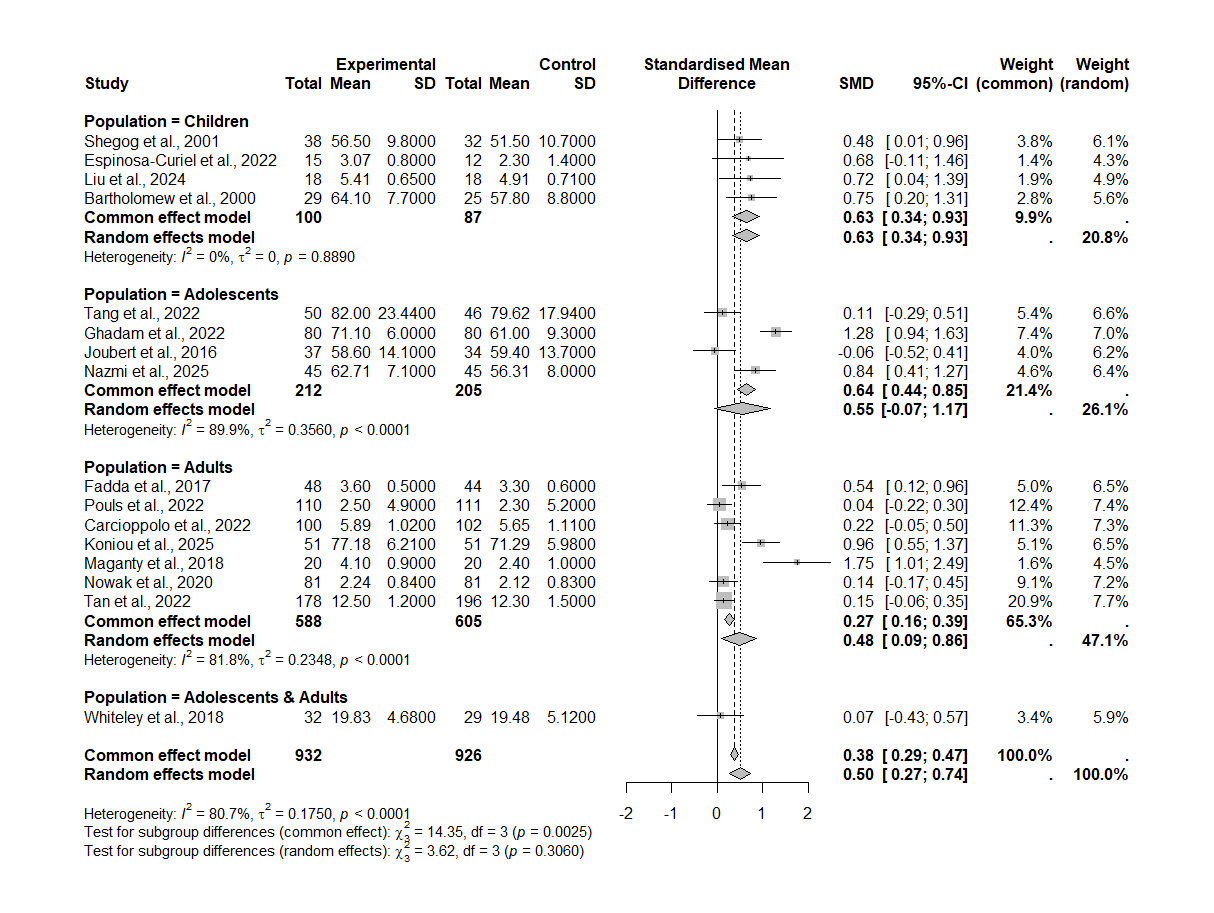
*

**Figure S18.** Forest plot of subgroup analysis by sex composition for attitude outcomes, comparing studies with higher versus lower proportions of female participants.

*
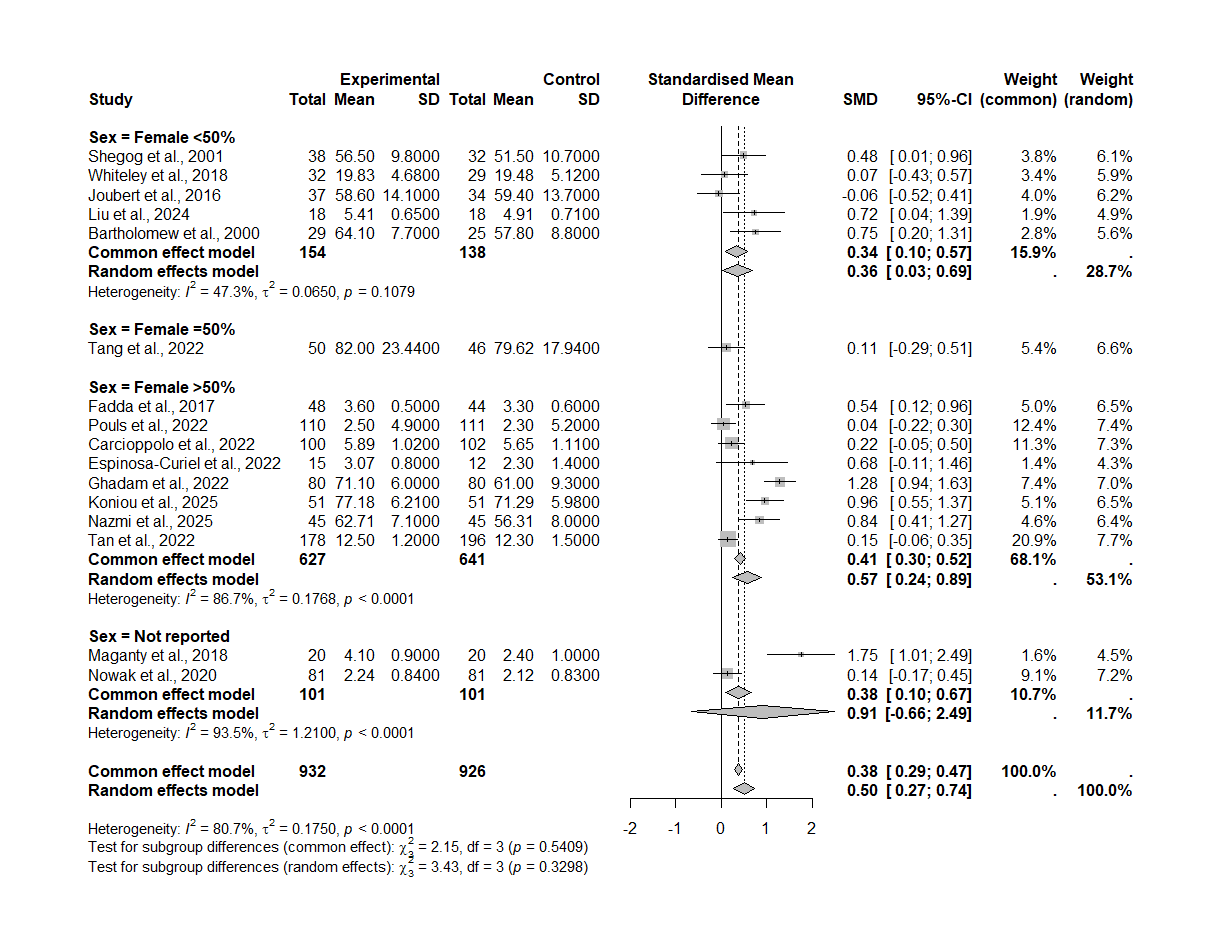
*
