# Supplementary material for: Effectiveness of Digital Serious Games on Knowledge and Attitudes in Public Health Education: Systematic Review and Bayesian Network Meta-Analysis of Randomized Controlled Trials
Source: J Med Internet Res. 2026 Apr 24;28:e89281. doi: 10.2196/89281 (PMC13108840; doi:10.2196/89281)

Multimedia Appendix 12a. Forest plot of the Bayesian network meta-analysis for knowledge outcomes, showing pooled mean differences with 95% credible intervals (CrI) and prediction intervals (PrI).

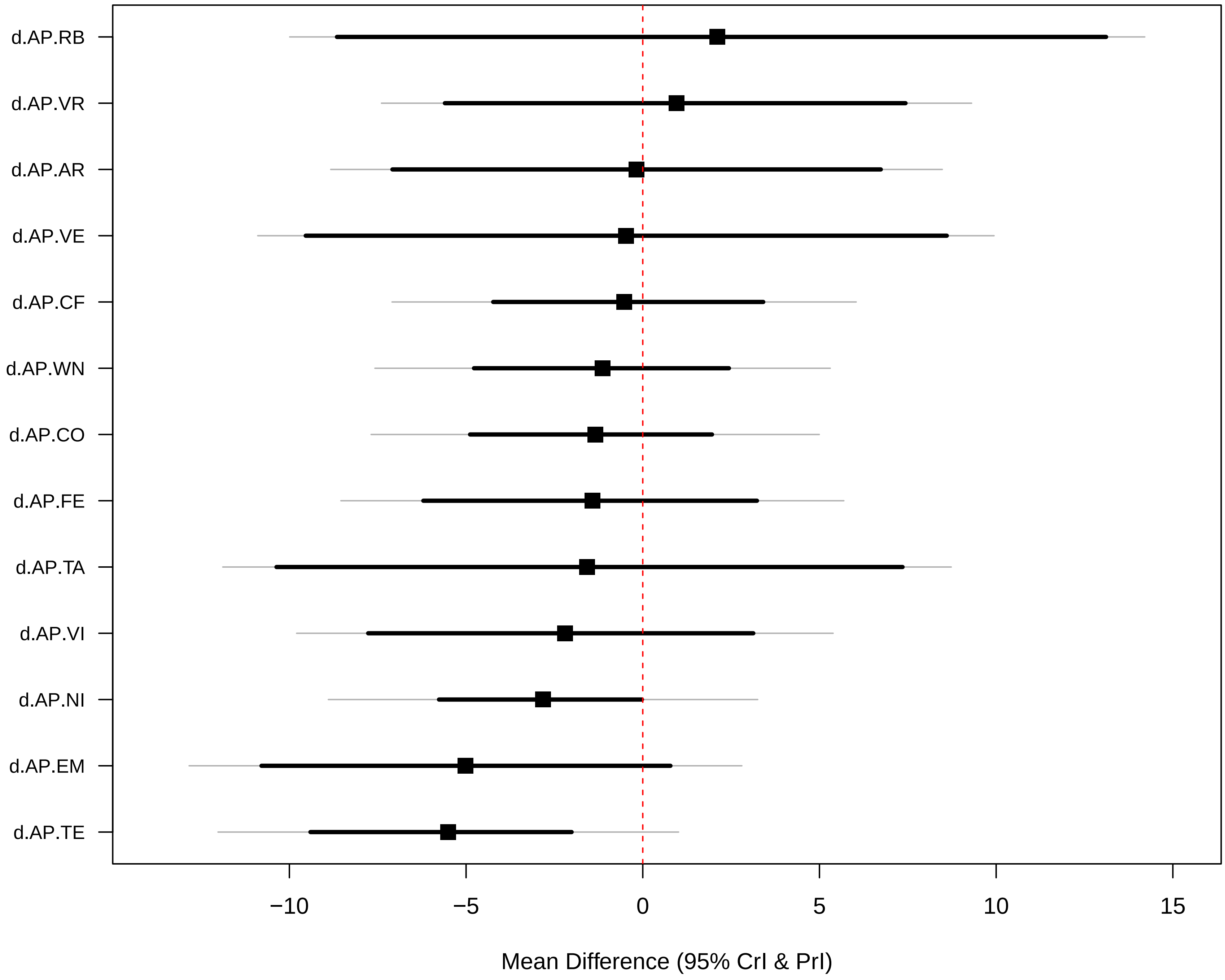

Appendix 12b. Forest plot of the Bayesian network meta-analysis for attitude outcomes, showing pooled mean differences with 95% credible intervals (CrI) and prediction intervals (PrI).

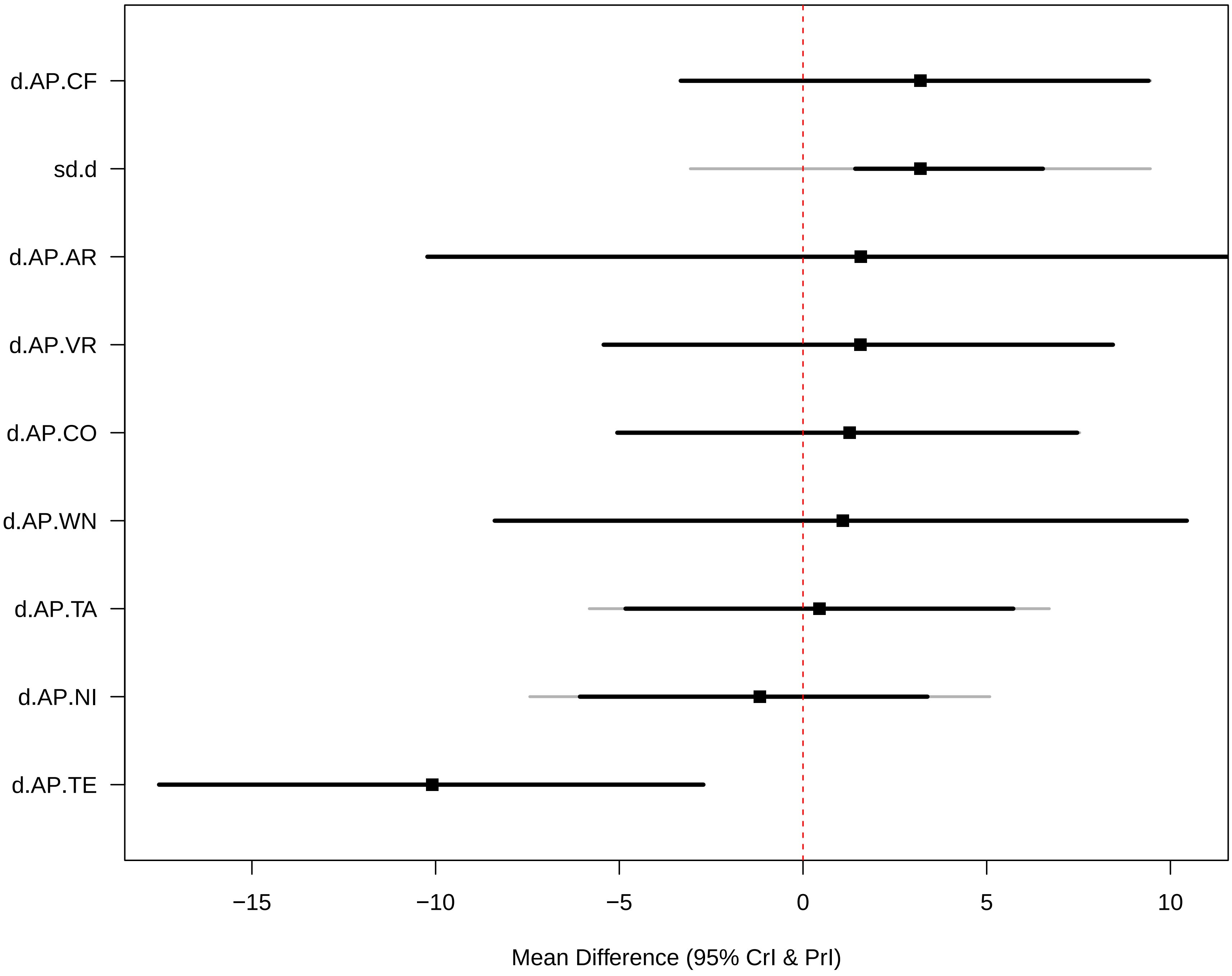

Supplement: Multimedia Appendix 11 [file jmir-v28-e89281-s011.pdf]
