# Supplementary material for: Effectiveness of Digital Serious Games on Knowledge and Attitudes in Public Health Education: Systematic Review and Bayesian Network Meta-Analysis of Randomized Controlled Trials
Source: J Med Internet Res. 2026 Apr 24;28:e89281. doi: 10.2196/89281 (PMC13108840; doi:10.2196/89281)

Multimedia Appendix 13a. Node-splitting analysis for the knowledge network, assessing inconsistency between direct and indirect evidence across treatment comparisons.

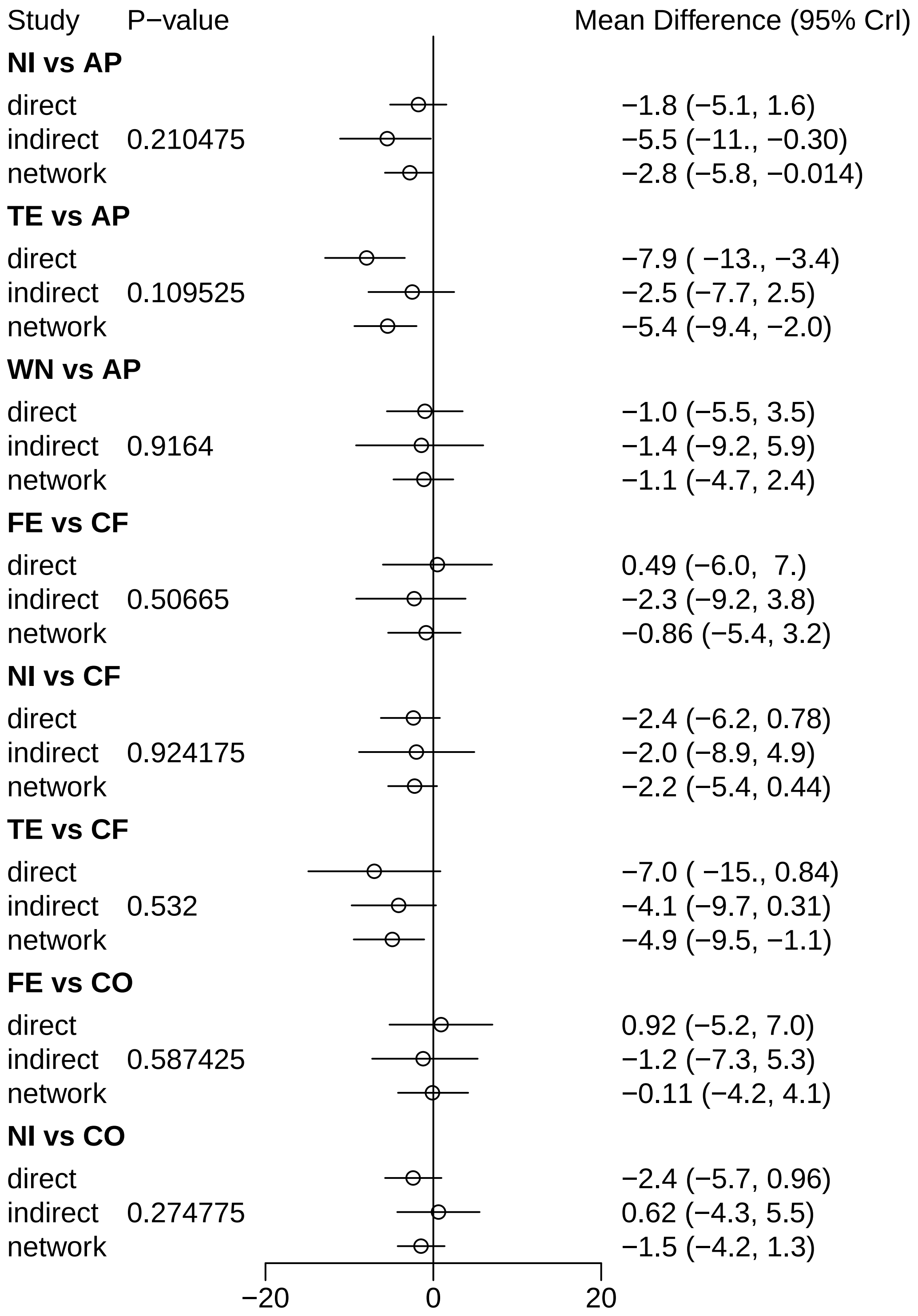

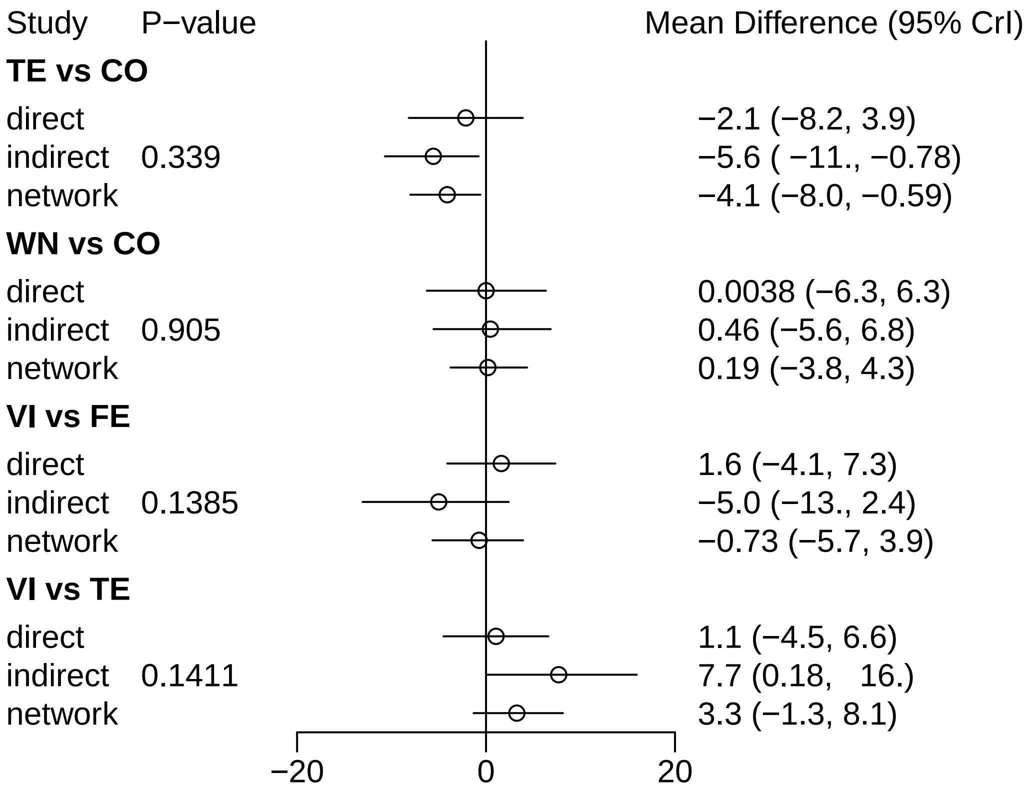
Study
P−value
Mean Difference (95% CrI)
TE vs CO
direct
−2.1 (−8.2, 3.9)
indirect
0.339
−5.6 (−11., −0.78)
network
−4.1 (−8.0, −0.59)
WN vs CO
direct
0.0038 (−6.3, 6.3)
indirect
0.905
0.46 (−5.6, 6.8)
network
0.19 (−3.8, 4.3)
VI vs FE
direct
1.6 (−4.1, 7.3)
indirect
0.1385
−5.0 (−13., 2.4)
network
−0.73 (−5.7, 3.9)
VI vs TE
direct
1.1 (−4.5, 6.6)
indirect
0.1411
7.7 (0.18, 16.)
network
3.3 (−1.3, 8.1)
−20
0
20

Appendix 13b. Node-splitting analysis for the attitude network, assessing inconsistency between direct and indirect evidence across treatment comparisons.

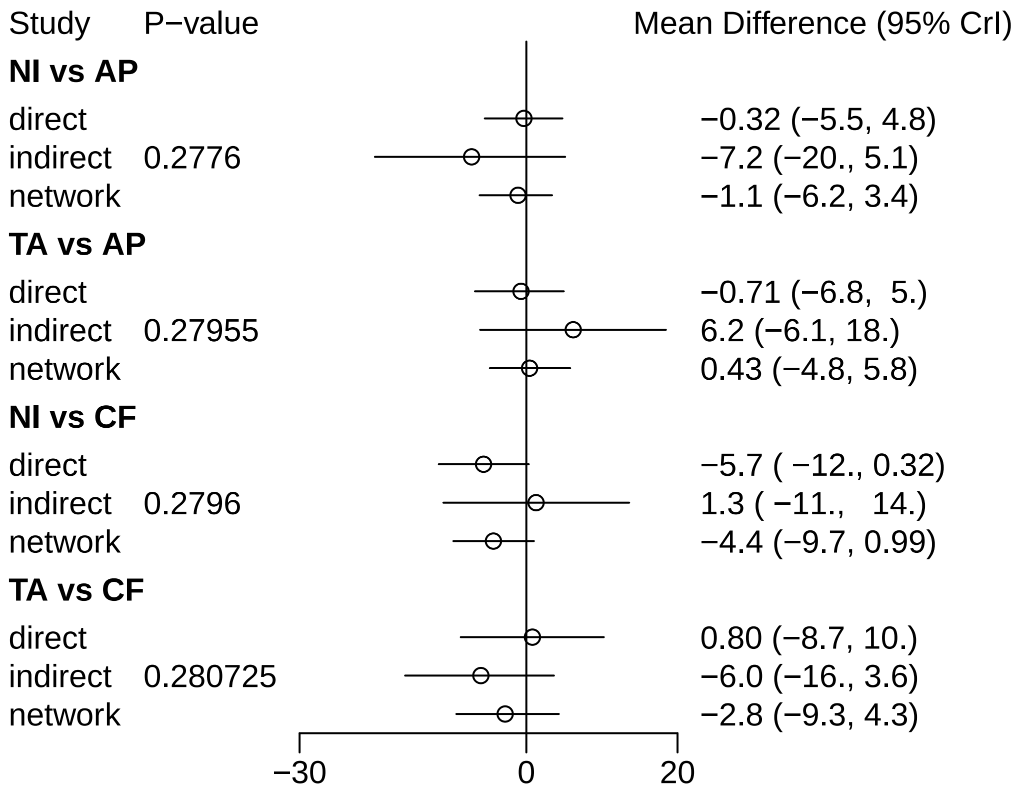

Supplement: Multimedia Appendix 12 [file jmir-v28-e89281-s012.pdf]
