# Supplementary material for: Effectiveness of Digital Serious Games on Knowledge and Attitudes in Public Health Education: Systematic Review and Bayesian Network Meta-Analysis of Randomized Controlled Trials
Source: J Med Internet Res. 2026 Apr 24;28:e89281. doi: 10.2196/89281 (PMC13108840; doi:10.2196/89281)
Supplement: Multimedia Appendix 13 [file jmir-v28-e89281-s013.docx]

**Multimedia Appendix 14a.** Knowledge outcome: comparison of SUCRA values and treatment rankings under uniform and half-normal prior distributions

| **Treatment** | **Main**  **(Uniform prior)** | **Sensitivity**  **(Half-normal prior)** | **Rank**  **(Main)** | **Rank**  **(Sensitivity)** |
| --- | --- | --- | --- | --- |
| AP | 0.752 | 0.766 | 1 | 2 |
| CO | 0.738 | 0.781 | 2 | 1 |
| WN | 0.706 | 0.724 | 3 | 3 |
| AR | 0.642 | 0.658 | 4 | 4 |
| CF | 0.623 | 0.607 | 5 | 5 |
| VE | 0.582 | 0.596 | 6 | 6 |
| RB | 0.548 | 0.541 | 7 | 7 |
| VR | 0.525 | 0.533 | 8 | 8 |
| FE | 0.503 | 0.499 | 9 | 9 |
| TA | 0.468 | 0.447 | 10 | 10 |
| VI | 0.418 | 0.413 | 11 | 11 |
| NI | 0.304 | 0.286 | 12 | 12 |
| EM | 0.182 | 0.128 | 13 | 13 |
| TE | 0.094 | 0.081 | 14 | 14 |

**Appendix 14b.** Attitude outcome: comparison of SUCRA values and treatment rankings under uniform and half-normal prior distributions

| **Treatment** | **Main**  **(Uniform prior)** | **Sensitivity**  **(Half-normal prior)** | **Rank**  **(Main)** | **Rank**  **(Sensitivity)** |
| --- | --- | --- | --- | --- |
| CO | 0.7421 | 0.7586 | 1 | 1 |
| WN | 0.7314 | 0.7448 | 2 | 2 |
| VR | 0.7042 | 0.7219 | 3 | 3 |
| AP | 0.6287 | 0.6431 | 4 | 4 |
| AR | 0.6126 | 0.6069 | 5 | 5 |
| CF | 0.5748 | 0.5897 | 6 | 6 |
| TA | 0.5419 | 0.5362 | 7 | 7 |
| VI | 0.5197 | 0.5306 | 8 | 8 |
| NI | 0.4983 | 0.4925 | 9 | 9 |
| RB | 0.4121 | 0.4056 | 10 | 10 |
| TE | 0.3042 | 0.2894 | 11 | 11 |
